# Supplementary material for: Strengthening contraceptive counselling services to empower clients and meet their needs: protocol for a two-stage, multiphase complex intervention in Pakistan and Nigeria
Source: BMJ Open. 2024 Jun 5;14(6):e081967. doi: 10.1136/bmjopen-2023-081967 (PMC11163613; doi:10.1136/bmjopen-2023-081967)
Supplement: Author's manuscript [file bmjopen-2023-081967.draft_revisions.pdf]

BMJ Open is committed to open peer review. As part of this commitment we make the peer review history of every article we publish publicly available.

When an article is published we post the peer reviewers' comments and the authors' responses online. We also post the versions of the paper that were used during peer review. These are the versions that the peer review comments apply to.

The versions of the paper that follow are the versions that were submitted during the peer review process. They are not the versions of record or the final published versions. They should not be cited or distributed as the published version of this manuscript.

BMJ Open is an open access journal and the full, final, typeset and author-corrected version of record of the manuscript is available on our site with no access controls, subscription charges or pay-per-view fees (http://bmjopen.bmj.com).

If you have any questions on BMJ Open's open peer review process please email info.bmjopen@bmj.com

BMJ Open

Strengthening contraceptive counselling services to empower clients and meet their needs: protocol for a 2-stage, multi-phase complex intervention in Pakistan and Nigeria.

Journal:	*BMJ Open*
Manuscript ID	bmjopen-2023-081967
Article Type:	Protocol
Date Submitted by the Author:	10-Nov-2023
Complete List of Authors:	Tran, Nguyen Toan; University of Technology Sydney,<br>Ali, Moazzam; World Health Organization, Department of Reproductive Health and Research<br>Azmat, Syed Khurram ; Jinnah Sindh Medical University, AAPNA - Institute of Public Health; Marie Stopes Society Pakistan, Technical Services<br>Seuc, Armando; World Health Organization, Department of Reproductive Health and Research<br>Olaolorun, Funmilola; University of Ibadan College of Medicine, Community Medicine<br>Awan, Muhammad Ali ; AAPNA Institute of Public Health (AIPH), Jinnah Sindh, Medical University, Rafiqui H.J, Iqbal Shaheed Rd, Karachi, Sindh 75510, Pakistan<br>Morhason-Bello, Imran; University of Ibadan College of Medicine, Department of Obstetrics and Gynaecology, Faculty of Clinical Sciences, College of Medicine/University College Hospital, University of Ibadan<br>Thom, Ellen Mpangananji ; World Health Organization Country Office in Islammabad Pakistan<br>Martin , Josepth ; World Health Organization Country Office in Abuja, Nigeria<br>Abubakar, Hanifah Datti ; Mohammed Abdullahi Wase Teaching Hospital, Nasarawa<br>Uzma, Qudsia ; World Health Organization Country Office in Islamabad Pakistan<br>Kiarie, James; World Health Organization, Department of Reproductive Health and Research
Keywords:	Clinical Trial, Decision Making, Health Services, Public health < INFECTIOUS DISEASES

SCHOLARONE™
Manuscripts

Strengthening contraceptive counselling services to empower clients and meet their needs: protocol for a 2-stage, multi-phase complex intervention in Pakistan and Nigeria.

Tran Nguyen Toan[1,2,3] nguyentoan.tran@uts.edu.au

Moazzam Ali[1*] alimoa@who.int

Syed Khurram Azmat[4] khurramazmat@gmail.com

Armando Seuc[1] armandoseuc@infomed.sld.cu

Funmilola M. Olaolorun PI[5] fmolaolorun@gmail.com

Muhammad Ali Awan[4] alighous@gmail.com

Imran Morhason-Bello[6] imranmorhasonbello@gmail.com

Ellen Mpangananji Thom[7] thome@who.int

Josepth Martin[8] mjoseph@who.int

Hanifah Datti Abubakar[9] hanniedee83@gmail.com

Qudsia Uzma[7] uzmaq@who.int

James Kiarie[1] kiariej@who.int

*Corresponding author: alimoa@who.int

[1] Department of Reproductive Health Research, World Health Organization, Avenue Appia 20, 1211 Genève 27, Switzerland.

[2] Faculty of Medicine, University of Geneva, Rue Michel-Servet 1, 1206 Genève, Switzerland

[3]Australian Centre for Public and Population Health Research, Faculty of Health, University of Technology, PO Box 123, Sydney, NSW 2007, Australia

[4] AAPNA Institute of Public Health (AIPH), Jinnah Sindh, Medical University, Rafiqui H.J, Iqbal Shaheed Rd, Karachi, Sindh 75510, Pakistan

[5] Department of Community Medicine, College of Medicine, University of Ibadan, Nigeria

[6]Department of Obstetrics and Gynaecology, College of Medicine, University of Ibadan, Nigeria

For peer review only - http://bmjopen.bmj.com/site/about/guidelines.xhtml

[7] World Health Organization Country Office in Islamabad Pakistan

[8] World Health Organization Country Office in Abuja, Nigeria

[9] Mohammed Abdullahi Wase Teaching Hospital, Nasarawa, Kano, Nigeria

Abstract

Background: High-quality contraceptive counseling can accelerate global efforts to reduce the unmet need for and suboptimal use of modern contraceptives. This study aims to identify a package of interventions designed to strengthen in Pakistan and Nigeria and determine their effectiveness in increasing client level decision-making, autonomy and meeting of contraceptive needs.

Methods: A multi-site, two-stage and five-phase intervention design will start with a pre-formative, formative, design, experimental and reflective phase. The Pre-formative phase will map potential study sites and establish the sampling frame. The two-part formative phase will first use participatory approaches to identify the perspectives of clients, including young couples and providers, to ensure research contextualization and address each interest group's needs and priorities followed by clinical observations of client-provider encounters to document routine care. The design workshop in the third phase will result in the development of a package of contraceptive counseling interventions. In the fourth experimental phase, a multi-intervention, three-arm, single-blinded, parallel cluster randomized-controlled trial will compare routine care (arm 1) with the contraceptive counseling package (arm 2) and the same package combined with wider method availability (arm 3). The reflective phase aims to identify implementation barriers and enablers. The primary outcomes are clients' level of decision-making autonomy and met need for modern contraceptives.

Discussion: Applying participatory action research principles in designing, testing, and scaling up effective, affordable, and sustainable counseling interventions could help optimize clients' decision-making autonomy and meet their needs for modern contraceptives in low-resource settings. Recognizing socio-cultural and health service complexities surrounding contraception, including client-provider power dynamics, study assumes that engaging key stakeholders, including adolescents, women, men, service providers, and policymakers would be more effective. A set of low-technology interventions will likely affect, at individual level and in a sustainable way, knowledge, attitudes, and behaviors of women and couples towards contraceptive counseling and provision.

Trial registration: Registered in ClinicalTrials.gov (NCT06081842, 31 October 2023).

For peer review only

Keywords

Health services research, counseling, contraception, family planning, empowerment, autonomy, qualitative research, cluster randomized controlled trial.

Strengths and limitations of this study

- First research using a qualitative study design followed by randomized controlled trial to assess the effectiveness of family planning counselling package. Fidelity intervention will be monitored to explore its effects on service users' outcomes.
- The inclusion of stakeholders at the start of research will enhance understanding of service provision dynamics and support efforts towards identifying strategies to enhance positive male involvement and engagement in women's sexual reproductive health and rights.
- Use reflective phase to identify operational barriers and enablers related to the package implementation using qualitative research to understand how to optimize the intervention.
- We will investigate the effect on proximal outcomes only, therefore have a limited insight into the effect on distal outcomes such as healthcare use.

Background

Overlooking women's and couples' need for quality contraceptive counseling constitutes a missed opportunities in family planning programs and services to ensure that every woman, man, and adolescent can enjoy the health, social, and economic benefits of modern contraception. Access to family planning is a human right enshrined in the United Nations' Sustainable Development Goal 3 on health and wellbeing and, specifically, its target 3.7: *ensure universal access to sexual and reproductive health-care services, including for family planning* [1]. Around 164 million women of reproductive age, have an unmet need for modern contraceptive methods despite increased contraceptive use over the past decades [2]. Satisfying the unmet need for contraception in low- and middle-income countries and offering all pregnant women and their newborns the standard care recommended by the World Health Organization (WHO) would result in dramatic reductions of unintended pregnancies by 68%, unsafe abortions by 72%, maternal deaths by 62%, and neonatal deaths by 69% [3]. Adequate contraceptive coverage among users can be improved by addressing suboptimal use and high discontinuation rates [4].

Effective contraceptive counseling can help individuals choose a method that meets their needs and preferences, manage any side effects, and continue using their preferred method or switch to an alternative one [5]. Contraceptive counseling could improve the effective use of modern contraception and reduce unmet need according to a systematic review that synthesized the evidence on the comparative effectiveness of different contraceptive counseling strategies [6]. The quality of contraceptive counseling in low-resource settings is often low. In Senegal, for example, only 18% of providers counseled their clients on the three-item Method Information Index (method use, side-effects, and timing of follow-up visits) [7]. Interventions targeting women

For peer review only - http://bmjopen.bmj.com/site/about/guidelines.xhtml

requesting or initiating a chosen method, including structured counseling on side-effects, tend to show positive effects on contraceptive continuation [8-11]. Counseling interventions targeting family planning service users [12-16], abortion service users [17-19], postpartum women [20-25], and those reached by community-based interventions [26-28] have inconsistent effects on contraceptive behavior and satisfaction outcomes. In contrast, increased contraceptive use often follows interventions introducing systematic contraceptive counseling among women attending non-family planning outpatient services [29-31]. However, these interventions often include expanded contraceptive method provision, and the observed changes cannot be attributed to counseling alone. High-quality evidence is generally limited, and no clear consensus exists on how best to deliver contraceptive counseling to meet clients' contraceptive needs and enhance their satisfaction.

Building on this systematic review, the World Health Organization (WHO) organized a geographically diverse think tank in May 2019 in Geneva, Switzerland, to identify research and guidance gaps and other opportunities to improve the quality of contraceptive counseling [32]. Equally important was the crafting of a definition on contraceptive counseling, which was adopted by think tank members [33]: *Contraceptive counseling is defined as the exchange of information on contraceptive methods based on an assessment of the client's needs, preferences and lifestyle to support decision-making as per the client's intentions. This includes the selection, discontinuation or switching of a contraceptive method. The key principles are based on coercion-free and informed choice; neutral, understandable and evidence-based information; collaborative and confidential decision-making process; ensuring respectful care, dignity and choice.* The definition took into consideration the core dimensions articulated in the literature and mirrors the principles that were deliberated by the think tank: a two-way discussion and decision-making process between the client and provider, sharing objective and user-friendly information, and fundamentally grounded in the rights to autonomy, dignity, privacy, respect, and participation, among others.

Furthermore, the think tank members agreed on a prioritized set of guidance, knowledge, and implementation research gaps [32]. Such gaps include, for example, understanding or identifying clients' experiences with and expectations of counseling in a variety of global settings; the types of settings different populations, particularly adolescents, unmarried women, migrants, gender non-conforming people, and religious/ethnic minorities, receive most of their contraceptive counseling, and the quality of counseling across these settings; the fluidity/ambivalence of pregnancy intentions, risk perception (pregnancy and sexually transmitted infections), and women's understanding of their bodies; the independent effect of counseling from method provision; the core elements of high-quality of contraceptive counseling and how these differ by context and what provider characteristics affect counseling quality; the minimum skills and competencies for contraceptive counselors; the contents of a comprehensive package of best practices for FP counseling for different populations.

Study overall objective and hypothesis

Owing to these gaps combined with the socio-cultural challenges related to accessing contraception, we propose a multi-site, two-stage and 5-phase study with two main objectives. The first objective is to identify and test the effectiveness of a package of contraceptive counseling interventions on improving clients' informed decision-making autonomy as well as

For peer review only - http://bmjopen.bmj.com/site/about/guidelines.xhtml

meeting their need for family planning. The second objective is to separate the effect of counseling from method provision on the same outcomes.

The research rests on the working hypotheses that the package will strengthen existing contraceptive counseling services; account for the views of key actors, including clients and providers; be in line with national health policies and WHO global standards; and take into consideration the scarcity of public resources.

Study countries: Pakistan and Nigeria

Pakistan

The study will be implemented in Pakistan in partnership with the Ministry of National Health Services, Regulations and Coordination.. The overall demographic, family planning, and reproductive health indicators based on the 2017-18 Demographic and Health Survey [34] note that rural settings fared worse than urban settings. The total fertility rate was 3.6 children per woman, modern contraceptive prevalance rate was 25% , overall unmet need for family planning was 17% and demand for family planning satisfied by a modern method 49%. In terms of family planning knowledge, more than 98% of urban and rural women had heard about any type of modern methods—before marriage, only 15% of women did. Regarding men's attitudes toward contraceptive use, 27% believed that family planning use is a woman's business, while 16% thought it may encourage women to be promiscuous.

Nigeria

The study will also be implemented in Nigeria by College of Medicine, University of Ibadan. The study will be conducted in two study sites: Lagos (southern part of Nigeria) and Kano (northern part of Nigeria) states. These states were selected for the following reasons: (1) Data from the Performance Monitoring for Action (PMA) program are available for both states from annual surveys over the past three years, including information on the method information index plus and other family planning indicators (pmadata.org); (2) The team has a good network in both locations and will be able to readily collaborate with the State Ministries of Health; (3) Safety and security are relatively good with no serious threats in recent times.

The latest demographic, family planning, and reproductive health indicators based on the 2018 Demographic and Health Survey in Nigeria in 2019 showed that in general, rural settings fared worse than urban settings. The total fertility rate was 5.3 children per woman; overall unmet need for family planning was 18.9%; demand for family planning satisfied by a modern method 33.9% and modern contraceptive prevalance rate was 12%. In terms of family planning knowledge, 93.9% of currently married women and 98.3% of sexually active unmarried women had heard about any type of modern contraceptive method.

Methods

Framework and perspectives

For peer review only

For peer review only - http://bmjopen.bmj.com/site/about/guidelines.xhtml

Theory of change

To ensure that the contraceptive counseling intervention package that will emerge from this trial strengthens existing family planning services and is effective, safe, sustainable, scalable, and culturally appropriate, this health services research is informed by the theory of change framework as well as perspectives on human rights and the health system.

The staged and phased approach adopted in this study, with the different specific objectives, levels of activities, and intermediate and final results (see Figure 1), is underpinned by the theory of change [37].

FIGURE 1 HERE

Health system perspective

First and related to the research model outlined in Figure 1, the study will rely on the WHO Health System Framework with its six system building blocks and link research and programmatic considerations for contraceptive counseling [38]. The six system building blocks are leadership/governance, health care financing, health workforce, medical products and technologies, information and research, and service delivery [38].

Human rights perspective

The research will use the WHO Framework for Ensuring Human Rights in the Provision of Contraceptive Information and Services [39]. The human rights framework comprises nine standards that interweave with the dimensions of quality of care: non-discrimination, availability, accessibility, acceptability, quality, informed-decision making, protection of privacy and confidentiality, participation, and accountability. The research team would ensure a person-oriented approach where no women or teenage girls are discriminated against (for example, in the provision of family planning information and services) because of their age, marital status, HIV status, minority status, or other medical or social factors.

With regard to the participation standard, the research will ensure active and informed participation of individuals in decision-making that affects them, including on matters related to their health [40]. Due to the complex social, cultural, and religious challenges and opportunities surrounding contraceptive use, our research design will assume that the following participatory action research (or participatory research) principles will be essential to meet the study objectives as well as gain insights into the implementation process. [41].

Multiple-intervention strategy

As a consequence of the adopted health system perspective and to ensure the implementation of a strategy that contributes to strengthened services, the research team departed from the idea of a single-intervention study. The team will consider the possibility of designing and testing not a single but several interventions to address effectively specific contraceptive

For peer review only - http://bmjopen.bmj.com/site/about/guidelines.xhtml

counseling barriers found in different building blocks. The contraceptive counseling interventions would then be aggregated into a package.

Study phases

The research entails a complex multi-site, two-stage and 5-phase (Figure 1): STAGE 1 is formative and will comprise of a pre-formative (phase 1) and a formative (phase 2). While the STAGE 2 is experimental and will include intervention design (phase 3), experimental (phase 4), and reflective (phase 5). The proposed methods of each phase are not intrinsically complex—the overall intervention is complex as it features a *number and difficulty of behaviors required by those delivering or receiving the intervention* and involves a *number of groups or organizational levels targeted by the intervention*, among others [42]. The design follows the recommendations from the Medical Research Council (2008/2010) to develop a complex intervention, determine its feasibility, and facilitate its implementation and evaluation [42]. Therefore, the phases are interconnected and have specific objectives, methodologies, and results.

STAGE 1: The specific objective of the pre-formative phase is to establish a sampling frame of potential study sites by mapping primary health centers according to pre-established criteria (both public and private facilities). Health centers are eligible if they (1) provide family planning services; (2) offer at least between three and four modern contraceptive methods, including a barrier method such as condom, and a short-term method such as pills or injectables, a long-term method such as implants intra-uterine device, and referrals for permanent methods to clients; (3) have no stock-outs of contraceptives during the preceding three months; (4) have on average at least 25 family planning encounters per week or 100 family planning encounters per months; (5) are not recipients of technical support for family planning (capacity building, supplies) from other organizations at the time of recruitment and commit not to receive any such support during the whole study; and (6) are willing to participate.. Based on this sampling frame, four health centers (public and private sector) will be allocated to the formative phase while the others will be randomized to each arm of the experimental phase. The specific objectives of the ***formative phase*** are to (1) document the in-clinic contraceptive counseling routine using observation checklists and (2) seek through qualitative research the perspectives of clients, including young people, providers, and other stakeholders to ensure adequate contextualization of the research and address needs and priorities of each interest group regarding contraceptive counseling barriers and enablers.

STAGE 2: The specific objective of the ***intervention design phase*** is to co-design, based on the results of the formative phase and through a participatory and consensus-building approach engaging clients, providers, and other main actors, a package of contraceptive counseling to be implemented during the experimental phase. To be integrated into the final package, interventions will need to consider current human resources and clinical capacity, costs, acceptability, feasibility, scalability, and other field considerations.

The specific objective of the ***experimental phase*** is to determine, through a cluster randomized controlled trial controlled (RCT) design, the effect of the contraceptive counseling package on clients' level of decision-making autonomy and met need for family planning. The three-armed cluster RCT comprise (1) a control arm with routine contraceptive counseling and care and a range of family planning methods that are routinely available at field level; (2) an

For peer review only - http://bmjopen.bmj.com/site/about/guidelines.xhtml

experimental arm implementing the contraceptive counseling package but still with a range of family planning methods that are routinely available at field level; and (3) another experimental arm with the contraceptive counseling package but with an expanded range of family planning methods that are recommended by national policy and guidance.

The specific objective of the ***reflective phase*** is to identify operational barriers and enablers related to the package implementation using qualitative research. Once the package of interventions has been identified—and resources permitting, we might consider conducting a cost-effectiveness analysis to determine the counseling package cost-effectiveness.

The qualitative research and cluster RCT are detailed in the following sections.

Qualitative research

General design

For the qualitative components of the study (in the formative and reflective phases), we will conduct in-depth interviews (IDIs) in Pakistan and Nigeria with members of the community (women , adolescent males and females, partners, people with influence), health staff (service providers, managers, Community Health Workers - CHWs), development partners, donor agencies, and national policymakers. The formative phase will involve four (4) conveniently sampled health centers (public and private) and their respective coverage areas in each country. These sites will be selected from the sampling frame determined in the pre-formative phase.

For the identification of contraceptive counselling barriers and enablers, separate IDIs of six to ten participants will be conducted for the following categories of individuals (1) women who have used family planning services at the health center in the previous 12 months–: married and unmarried adolescent females (≤ 19 years), married and unmarried women 19-39 years, married and unmarried women ≥ 40 years [The study will include Women of Reproductive Age (WRA) - irrespective of their marital status and age group (including migrants refugee people, ethnic and religious minorities, women or men with disabilities, women or men with HIV etc. are inclusive] ; (2) married and unmarried men who are partners or husbands of women who have used family planning services at the health center in the previous 12 months, and married and unmarried adolescent males (<19 years); and (3) family planning service providers. IDIs will also take place with clinic managers, supervisors, CHWs, policy makers, and other influential individuals/opinion leaders who have observed, supervised, or monitored family planning services. We anticipate that interviews will last between 45-60 mins and will take place in a safe, neutral space of the participant's choosing (e.g. town hall, office space, on porch outside participant's home, etc.). Both the recruiter and the participant will decide and agree on the location of the interview and this will be conveyed to the research team. Alternatively, the team will also identify pay-per-hour spaces as an option to present to the participant. The interview guides will cover the following themes related to contraceptive counselling: knowledge, attitudes, and practices; socio-cultural and health systems barriers and catalysts; and quality of care, including autonomy, informed decision-making, and other human rights considerations.

The qualitative research in the ***reflective phase*** will occur in three health centers in each of the three study arms (nine centers in total). The centers will be purposely sampled to include the lowest, average, and highest performing ones in terms of measured outcomes to enable the

For peer review only - http://bmjopen.bmj.com/site/about/guidelines.xhtml

broadest possible understanding. In line with the theory of change approach, the qualitative research will consist of an implementation evaluation to analyze barriers and enablers related to the delivery of contraceptive counseling and family planning services, assess intervention fidelity (in the experimental groups), and explore approaches for scaling up. Separate IDIs are planned for service providers and clients (≤ 19 years, 20-39 years, ≥ 40 years). IDIs are planned for clinic managers. The interview guides will cover the following themes related to contraceptive counseling: operational barriers and enablers; quality of care, including decision-making autonomy and other human rights perspectives; and scaling-up considerations.

Selection criteria and recruitment approach: A standard process of selection and consent-taking will be undertaken at each study site comprising potential study participants for each of the above categories of individuals (including vulnerable populations). A recruitment approach will follow, and the main actors who will facilitate the recruitment process of the potential target respondents for this research are a) selected service providers, b) Community Health Workers – working in the catchment area of the selected service providers, c) community-based opinion leaders, i.e., male, and female schoolteachers, social activist/ NGO workers. The country PI/Co-PI will train all of them on the study's main objectives, purpose, issues in ethics and the importance of consent.

Qualitative data management and analysis

At the country level, the study will engage a team of reseachers with relevant experience in qualitative methods and in working in local languages.

The interviews will occur in a location guaranteeing privacy. We anticipate that interviews will last between 45-60 mins and will take place in a safe, neutral space of the participant's choosing (e.g. town hall, office space, on porch outside participant's home, etc.). The interviews will be audiotaped after obtaining informed conent from participants. Research assistants with experience in transcription will transcribe and translate the audio records into English. Accuracy checks will be done by comparing transcripts with audio files.

Qualitative research management Softwares such as QSR NVivo 12 or Atlas.ti , , will be used to perform a thematic analysis by establishing a basic codebook describing all the nodes and using it to code data. The codebook will be enriched with new emerging nodes during the coding process. Data will be coded by research assistants competent in NVivo. To ensure the quality of data coding, inter-rater reliability will be tested by computing the Cohen's Kappa coefficient with a Kappa coefficient of 0.8 and above as an acceptable concordance threshold.

The formative phase results will be presented and discussed during the intervention design workshop with key members of the research team, selected service providers, and clients with a view to informing the bottom-up design of a contextually sensitive package of contraceptive counseling interventions. The views of each workshop participant will be considered when examining the potential effectiveness of possible interventions, acceptability to women and providers, feasibility, the potential for integration into the overall family planning programs, sustainability, and scalability.

For peer review only - http://bmjopen.bmj.com/site/about/guidelines.xhtml

Cluster randomized controlled trial

The research will be a multi-intervention, three-arm, single-blinded, parallel, cluster RCT done in selected primary health centers (clusters) in Pakistan and in Nigeria (see **Figure 2** for the cluster RCT flow diagram, and **Figure 3** for the schedule of enrolment, interventions, and assessments (SPIRIT template). Centers will be randomly allocated to the three study arms in matched ratios (1:1:1) on the basis of the number of monthly family planning encounters, the number of available contraceptive types, the ratio of health workers per population in the clinic coverage zone, the location in urban or rural settings, and selected district-level variables that may have an influence on the study outcomes, including the unmet need for family planning, the level of literacy among women, and household income terciles. Only data analysts will be masked to trial-arm allocation. Service providers skilled in family planning services will implement the clinical components of the contraceptive counseling package.

FIGURE 2 HERE

FIGURE 3 HERE

Outcome measures

Quantitative indicators will be captured in case-report forms (CRFs) developed and piloted at the country level before finalization. The proposed primary outcomes to be assessed after each visit, at mid-point (6 months after enrollment), and at the study end (12 months after enrollment) are oriented toward clients and consist of levels of clients' decision-making autonomy and met need for family planning.

To determine their level of decision-making autonomy, clients will be asked to answer questions about the different domains contained in the counseling definition (see Background). These domains comprise client-provider collaboration, client's needs and preferences, the quality of information, freedom from coercion, confidentiality, and dignity. The Person-Centered Contraceptive Counseling scale (PCCC), developed and tested in publicly-funded clinics in California will also be used [43]. The four PCCC questions fall under the domains of respect, eliciting—and giving serious consideration to—client's preferences, and information quality.

The study proposes nonetheless the following secondary outcome, while acknowledging its limitations: modern contraceptive prevalence after the first visit and at 6 and 12 months after enrollment.

Eligibility criteria for clusters (health centers) and participants (women)

For peer review only - http://bmjopen.bmj.com/site/about/guidelines.xhtml

The study will be conducted in selected primary health centers. The eligibility criteria of health centers were described above under the objective of the pre-formative phase. A woman will be eligible to participate in the study if (1) she comes to the family planning clinic with the intention to (i) use contraception for the first time in her life (new user), or (ii) switch from a contraceptive method to another one (switching user), or (iii) resume a method after not using any in the prior three months (lapse user), or (iv) discontinue a modern method (discontinuing user); (2) she is not coming for the resupply of a currently used method, such as pills or injectables; (3) she has the intention to continue her follow-up at the health center during the 12-month study follow-up; (4) she does not participate in another study; and (5) she provides informed consent. Criteria (2) will ensure that clients who are satisfied with their methods and come to the clinic for resupply (such as pills or injectables)—and, therefore, are unlikely to necessitate contraceptive counseling—will not be enrolled. Conversely, criteria (1) focuses on women who need to see a contraceptive provider to initiate, switch methods, discontinue, or resume a method.

Interventions

The health centers of the first experimental group will implement the intervention package, and those of the second experimental group will implement the intervention package in addition to an expanded range of family planning methods that are recommended by national guidance. The control group will implement usual care. Depending on the nature of the final package, some interventions may be implemented before participant recruitment, such as the capacity building of providers.

Randomization

In each country, the 36 sites will be matched on a 1:1:1 ratio according to the above-described criteria. Within each of the 12 trios, study sites will be randomly assigned to each of the three study arms. This randomization is performed 12 times (once for each trio). No restriction in the randomization process is required and masking cannot be done except for data analysts. All consecutive and eligible participants will be included in the clusters until the sample size is achieved.

Data source and collection

Data on key outcome measures as well as quantitative process indicators will be collected on paper-based/electronic CRFs. The WHO team in Geneva will develop the CRFs with inputs from the country research teams. We will test advanced drafts with an appropriate sample of service providers and mock clients from the formative phase sites before finalizing the forms. Each health center of the cluster RCT will have a research assistant who will be trained to adhere to the study manual and standard operating procedures for data management which are common to both study countries and have been developed by WHO. The information reported on the CRFs will be checked for accuracy and completeness several times at different levels by field coordinators and data managers.

Data management

For peer review only - http://bmjopen.bmj.com/site/about/guidelines.xhtml

As for other multicenter studies conducted and sponsored by the UNDP/UNFPA/UNICEF/WHO/World Bank Special Programme of Research, Development and Research Training in Human Reproduction (HRP) at WHO in Geneva, data entry and data quality monitoring will be carried out using a web-based data management system. It will be developed by HRP's Biostatistics and Data Management team using OpenClinica (version 3.16), an electronic data capture software for clinical research that includes programmed edit checks for data quality, completeness, and consistency reduces delays in data queries and problem resolution and allows the dataset to be easily available. The OpenClinica system is a highly secure, open-source, and trusted web-based software solution that is fully compliant with Good Clinical Practice (GCP) and regulatory guidelines and HRP/WHO Standard Operating Procedures for clinical trial management.

The country research teams in Pakistan and Nigeria will enter the data locally and have it reviewed by the WHO team in Geneva. We will train the country teams in data entry and data management through OpenClinica system. Country research teams will be responsible for verifying the data, uploading it onto shared files, and entering the data into the OpenClinica system. Double data entry will be done to minimize errors. The system can produce automatic data queries (missing values, outliers, inconsistencies, and other errors). Data quality will be monitored by the WHO team. Questions regarding data inconsistencies or missing values will be submitted to sites via standardized forms and solved on a continuous basis. To ensure data integrity and participant confidentiality, data transmission will be encrypted. Only approved users will be given access to the data, which will be password protected.

Statistical analysis

We will prepare a comprehensive statistical analysis plan. Participants will form the unit of analysis and the clustered nature of the data will be accounted for. All analyses will be by intention to treat. For continuous variables, we will compute means, standard deviations, minimum and maximum values, and for categorical variables, we will compute frequencies and percentages. We will look at the distribution of variables and see if there are any outliers as part of data quality control and descriptive analysis. Descriptive statistics will be tabulated for individual clusters and aggregated across clusters. We will use IBM SPSS Statistics 25, R 3.6.2, STATA 16.1, or SAS 9.4 statistical packages for the analysis. The findings will be presented using the CONSORT Statement checklist from 2010 and its extension for cluster studies [44].

Ethics approvals

We followed the guidelines in The Ottawa Statement on the Ethical Design and Conduct of Cluster Randomized Trials while designing this experiment [45]. The Research Project Review Panel (RP2) of the HRP peer-reviewed the protocol, and RP2 and the WHO Research Ethics Review Committee, Geneva, Switzerland (Pakistan: ERC 006232 and Nigeria ERC: 006523), approved it and so did the IRB of Research and Development Solutions (IRB No:00010843) in Pakistan. While the Ministry of Health, Kano State of Nigeria (NHREC 17/03/2018) and Lagos Research Ethics Committee in Nigeria (LREC/06/10/2180) provided ethical approvals for the Nigeria study .

For peer review only - http://bmjopen.bmj.com/site/about/guidelines.xhtml

This research is grounded in human rights principles, specifically participation in the form of participatory action research. The existing literature shows promise that our proposed approach may be successful. For example, a synthesis of the evidence on community-based participatory research in different settings indicates that collaboration among community partners, researchers, and organizations led to community-level action that enhanced health and wellbeing while minimizing health disparities [46]. In the process, it also strengthened the capacity of the community in terms of research and leadership skills. Another review found that participatory action research could ensure that research is appropriate culturally and logistically; enhance recruitment capacity; generate professional capacity and competence in stakeholder groups; result in productive conflicts followed by useful negotiation; increase the quality of outputs and outcomes over time; increase the sustainability of project goals beyond funded time frames and during gaps in external funding; and create system changes and new unanticipated projects and activities [47].

For peer review only

For peer review only - http://bmjopen.bmj.com/site/about/guidelines.xhtml

Discussion

Ensuring access to contraception is fundamental to human rights and contributes to improved health outcomes, as recognised in Sustainable Development Goal 3.7 (universal access to sexual and reproductive healthcare services).

Quality contraceptive information and services reinforce people's freedom to determine the number and spacing of their children and offer a range of potential benefits encompassing women's empowerment, economic development, education and improved health outcomes, including maternal and child health.

Contraceptive counselling can help clients choose a method meeting their needs and preferences, manage side effects, and support method continuation or switching. High-quality counselling therefore has a high potential to strengthen efforts to reduce unmet need for contraception.

The results and insights gleaned from this study will provide a better understanding of the counselling strategies to improve contraceptive use, increased satisfaction and decrease discontuination of the contraceptive methods leading to achievement of global targets in reprorucitve health.

Declarations

Ethics approval and consent to participate

The research protocol was approved by the Research Project Review Panel (RP2) of the UNDP/UNFPA/UNICEF/WHO/World Bank Special Programme of Research, Development and Research Training in Human Reproduction (HRP) at WHO in Geneva, and the WHO Research Ethics Review Committee, Geneva, Switzerland. The study was approved by the ethics review committee in Pakistan and Nigeria. All adult participants will provide written consent. Non-adult participants will give their informed assent and their respective parents or guardians their informed consent.

Consent for publication:

Not applicable.

Availability of data and materials

Not applicable.

Competing interests

The authors declare that they have no competing interests.

For peer review only - http://bmjopen.bmj.com/site/about/guidelines.xhtml

Patient and public involvement Patients and/or the public were not involved in the design, or conduct, or reporting, or dissemination plans of this research.

Funding

The project site in Pakistan has been supported by the UNDP-UNICEF-WHO-World Bank Special Programme of Research, Development and Research Training in Human Reproduction (HRP); a co-sponsored programme executed by the World Health Organisation (WHO). The project in Nigeria generously received funding from United States Agency for International Development (USAID grant No. GHA-G-00-09-00003).

Authors' contributions

TNT, MA, and AS designed the initial study concept, the study protocol, and study instruments. SKA,MA, MAA and FMO provided revisions to multiple versions of the study protocol based on the feedback from WHO-ERC. SKA, MA, FMO, MAA, EMT, JM, QU, JK, HDA and IMB contributed to the final study protocol and study instruments. TNT wrote the first draft of the manuscript with the contributions of MA, AS, and KJ. While, SKA and MA developed the final draft of the manuscript. All authors read and approved the final manuscript.

Acknowledgements

The authors are grateful to Dr. Onikepe Owolabi of the Guttmacher Institute and Dr. Nathalie Roos of the Karolinska Institute for their technical support and insights.

ORCID:

Moazzam Ali http://orcid.org/0000-0001-6949-8976

References

1. Howden-Chapman P, Siri J, Chisholm E, Chapman R, Doll CN, Capon A: **SDG 3: Ensure healthy lives and promote wellbeing for all at all ages**. *A guide to SDG interactions: from science to implementation Paris, France: International Council for Science* 2017:81-126.
2. United Nations Department of Economic and Social Affairs, Population Division (2022). World Family Planning 2022: Meeting the changing needs for family planning: Contraceptive use by age and method.UN DESA/POP/2022/TR/NO. 4
3. Sully E, Biddlecom A, Darroch J, Ashford L, Lince-Deroche N, Riley T: **Adding It Up: Investing in Sexual and Reproductive Health, 2020**. *New York, NY: Guttmacher Institute*.

For peer review only - http://bmjopen.bmj.com/site/about/guidelines.xhtml

4. Polis CB, Bradley SE, Bankole A, Onda T, Croft T, Singh S: **Contraceptive failure rates in the developing world: an analysis of demographic and health survey data in 43 countries**. *New York: Guttmacher Institute* 2016.

5. Dehlendorf C, Krajewski C, Borrero S: **Contraceptive counseling: best practices to ensure quality communication and enable effective contraceptive use**. *Clinical obstetrics and gynecology* 2014, **57**(4):659.

6. Cavallaro FL, Benova L, Owolabi OO, Ali M: **A systematic review of the effectiveness of counselling strategies for modern contraceptive methods: what works and what doesn't?** *BMJ Sexual & Reproductive Health* 2019.

7. Assaf S, Wang W, Mallick L: **Provider Counseling and Knowledge Transfer in Health Facilities of Haiti, Malawi, and Senegal. DHS Analytical Studies No. 60**. *Rockville, Maryland* 2016.

8. De Cetina TEC, Canto P, Luna MO: **Effect of counseling to improve compliance in Mexican women receiving depot-medroxyprogesterone acetate**. *Contraception* 2001, **63**(3):143-146.

9. Modesto W, Bahamondes MV, Bahamondes L: **A randomized clinical trial of the effect of intensive versus non-intensive counselling on discontinuation rates due to bleeding disturbances of three long-acting reversible contraceptives**. *Hum Reprod* 2014, **29**(7):1393-1399.

10. Patel D, Koenig M, Patel A, Rani M: **The impact of improved service quality on IUD continuation: evidence from rural India**. *JOURNAL OF FAMILY WELFARE* 2003, **49**:1-9.

11. Amatya R, Akhter H, McMahan J, Williamson N, Gates D, Ahmed Y: **The effect of husband counseling on NORPLANT® contraceptive acceptability in Bangladesh**. *Contraception* 1994, **50**(3):263-273.

12. Jain AK, RamaRao S, Kim J, Costello M: **Evaluation of an intervention to improve quality of care in family planning programme in the Philippines**. *Journal of biosocial science* 2012, **44**(1):27.

13. Chin-Quee DS, Janowitz B, Otterness C: **Counseling tools alone do not improve method continuation: further evidence from the decision-making tool for family planning clients and providers in Nicaragua**. *Contraception* 2007, **76**(5):377-382.

14. Sanogo D, RamaRao S, Jones H, N'diaye P, M'bow B, Diop CB: **Improving quality of care and use of contraceptives in Senegal**. *African journal of reproductive health* 2003:57-73.

15. Sapkota S, Rajbhandary R, Lohani S: **The impact of balanced counseling on contraceptive method choice and determinants of long acting and reversible contraceptive continuation in Nepal**. *Maternal and child health journal* 2017, **21**(9):1713-1723.

16. Kim YM, Putjuk F, Basuki E, Kols A: **Increasing patient participation in reproductive health consultations: an evaluation of "Smart Patient" coaching in Indonesia**. *Patient education and counseling* 2003, **50**(2):113-122.

17. Zhu JL, Zhang W-H, Cheng Y, Xu J, Xu X, Gibson D, Støvring H, Claeys P, Temmerman M: **Impact of post-abortion family planning services on contraceptive use and abortion rate among young women in China: a cluster randomised trial**. *The European Journal of Contraception & Reproductive Health Care* 2009, **14**(1):46-54.

18. Ferreira ALCG, Souza AI, Pessoa RE, Braga C: **The effectiveness of contraceptive counseling for women in the postabortion period: an intervention study**. *Contraception* 2011, **84**(4):377-383.

19. Smith C, Ngo TD, Gold J, Edwards P, Vannak U, Sokhey L, Machiyama K, Slaymaker E, Warnock R, McCarthy O: **Effect of a mobile phone-based intervention on post-abortion contraception: a randomized controlled trial in Cambodia**. *Bulletin of the World Health Organization* 2015, **93**:842-850A.

20. Kaewkiattikun K: **Effects of immediate postpartum contraceptive counseling on long-acting reversible contraceptive use in adolescents**. *Adolescent health, medicine and therapeutics* 2017, **8**:115.

For peer review only - http://bmjopen.bmj.com/site/about/guidelines.xhtml

21. Bolam A, Manandhar DS, Shrestha P, Ellis M, Anthony MdL: **The effects of postnatal health education for mothers on infant care and family planning practices in Nepal: a randomised controlled trial**. *Bmj* 1998, **316**(7134):805-811.
22. Adanikin AI, Onwudiegwu U, Loto OM: **Influence of multiple antenatal counselling sessions on modern contraceptive uptake in Nigeria**. *Eur J Contracept Reprod Health Care* 2013, **18**(5):381-387.
23. Vural F, Vural B, Cakiroglu Y: **The effect of combined antenatal and postnatal counselling on postpartum modern contraceptive use: prospective case-control study in Kocaeli, Turkey**. *Journal of clinical and diagnostic research: JCDR* 2016, **10**(4):QC04.
24. Fatima P, Antora AH, Dewan F, Nash S, Sethi M: **Impact of contraceptive counselling training among counsellors participating in the FIGO postpartum intrauterine device initiative in Bangladesh**. *International Journal of Gynecology & Obstetrics* 2018, **143**:49-55.
25. Ndegwa SW, Gichuhi J, Qureshi Z, Lubano K: **The effect of two levels of counselling on acceptance, uptake and early outcomes of post-placental intra-uterine contraceptive device**. *East African medical journal* 2014, **91**(12):449-456.
26. Terefe A, Larson CP: **Modern contraception use in Ethiopia: does involving husbands make a difference?** *American Journal of Public Health* 1993, **83**(11):1567-1571.
27. El-Khoury M, Thornton R, Chatterji M, Kamhawi S, Sloane P, Halassa M: **Counseling women and couples on family planning: a randomized study in Jordan**. *Studies in family planning* 2016, **47**(3):222-238.
28. Lemani C, Tang JH, Kopp D, Phiri B, Kumvula C, Chikosi L, Mwale M, Rosenberg NE: **Contraceptive uptake after training community health workers in couples counseling: A cluster randomized trial**. *PloS one* 2017, **12**(4):e0175879.
29. Gillespie D, Bradley H, Woldegiorgis M, Kidanu A, Karklins S: **Integrating family planning into Ethiopian voluntary testing and counselling programmes**. *Bulletin of the World Health Organization* 2009, **87**:866-870.
30. Lee J, Papic M, Baldauf E, Updike G, Schwarz EB: **A checklist approach to caring for women seeking pregnancy testing: effects on contraceptive knowledge and use**. *Contraception* 2015, **91**(2):143-149.
31. Yassin AS, Cordwell D: **Does dedicated pre-abortion contraception counselling help to improve post-abortion contraception uptake?** *BMJ Sexual & Reproductive Health* 2005, **31**(2):115-116.
32. Ali M, Tran NT, Kabra R, Kiare J: **Strengthening contraceptive counselling: gaps in knowledge and implementation research**. In.: British Medical Journal Publishing Group; 2021.
33. Ali M, Tran NT: **Defining counselling in contraceptive information and services: outcomes from an expert think tank**. In.: British Medical Journal Publishing Group; 2021.
34. National Institute of Population Studies: **Pakistan Demographic and Health Survey 2017–18. Available at: https://dhsprogram.com/pubs/pdf/FR290/FR290.pdf (2019). Accessed 1 April 2021.**
35. National Institute of Population Studies: **Pakistan Special Demographic and Health Survey 2019. Available at: https://dhsprogram.com/publications/publication-SR267-Summary-Reports-Key-Findings.cfm. Accessed 1 April 2021**
36. National Population Commission (NPC) [Nigeria] and ICF. 2019. **Nigeria Demographic and Health Survey 2018**. Abuja, Nigeria, and Rockville, Maryland, USA: NPC and ICF
37. De Silva MJ, Breuer E, Lee L, Asher L, Chowdhary N, Lund C, Patel V: **Theory of Change: a theory-driven approach to enhance the Medical Research Council's framework for complex interventions**. *Trials* 2014, **15**:267.

For peer review only - http://bmjopen.bmj.com/site/about/guidelines.xhtml

38. World Health Organization: **Everybody's business--strengthening health systems to improve health outcomes: WHO's framework for action**. Geneva: World Health Organization; 2007. https://www.who.int/healthsystems/strategy/everybodys_business.pdf. Accessed 15 May 2020.
39. World Health Organization: **Framework for ensuring human rights in the provision of contraceptive information and services**. In. Edited by World Health Organization. Geneva: World Health Organization,; 2014.
40. Committee on Economic Social and Cultural Rights (22nd session): **General comment No 14: The right to the highest attainable standard of health–Article 12 of the International Covenant on Economic, Social and Cultural Rights (CESCR)**. *Geneva: United Nations Economic and Social Council* 2000.
41. Macaulay AC, Commanda LE, Freeman WL, Gibson N, McCabe ML, Robbins CM, Twohig PL: **Participatory research maximises community and lay involvement**. *Bmj* 1999, **319**(7212):774-778.
42. Craig P, Dieppe P, Macintyre S, Michie S, Nazareth I, Petticrew M: **Developing and evaluating complex interventions: new guidance. Medical Research Council, 2008**. In.; 2010.
43. Dehlendorf C, Fox E, Silverstein IA, Hoffman A, Pérez MPC, Holt K, Reed R, Hessler D: **Development of the Person-Centered Contraceptive Counseling scale (PCCC), a short form of the Interpersonal Quality of Family Planning care scale**. *Contraception* 2021.
44. Zwarenstein M, Treweek S, Gagnier JJ, Altman DG, Tunis S, Haynes B, Oxman AD, Moher D: **Improving the reporting of pragmatic trials: an extension of the CONSORT statement**. *Bmj* 2008, **337**:a2390.
45. Weijer C, Grimshaw JM, Eccles MP, McRae AD, White A, Brehaut JC, Taljaard M: **The Ottawa Statement on the Ethical Design and Conduct of Cluster Randomized Trials**. *PLoS Med* 2012, **9**(11):e1001346.
46. Salimi Y, Shahandeh K, Malekafzali H, Loori N, Kheiltash A, Jamshidi E, Frouzan AS, Majdzadeh R: **Is Community-based Participatory Research (CBPR) Useful? A Systematic Review on Papers in a Decade**. *International Journal of Preventive Medicine* 2012, **3**(6):386-393.
47. Jagosh J, Macaulay AC, Pluye P, Salsberg J, Bush PL, Henderson J, Sirett E, Wong G, Cargo M, Herbert CP: **Uncovering the benefits of participatory research: implications of a realist review for health research and practice**. *The Milbank Quarterly* 2012, **90**(2):311-346.

For peer review only - http://bmjopen.bmj.com/site/about/guidelines.xhtml

Figures, tables, additional files

Figure 1. Overall study model.

Figure 2. Flow diagram of the cluster randomized control trial in each study country.

Figure 3. Schedule of enrolment, interventions, and assessments for the cluster randomized controlled trial phase in each study country (SPIRIT template).

For peer review only

For peer review only - http://bmjopen.bmj.com/site/about/guidelines.xhtml

Figures

Figure 1. Overall study model

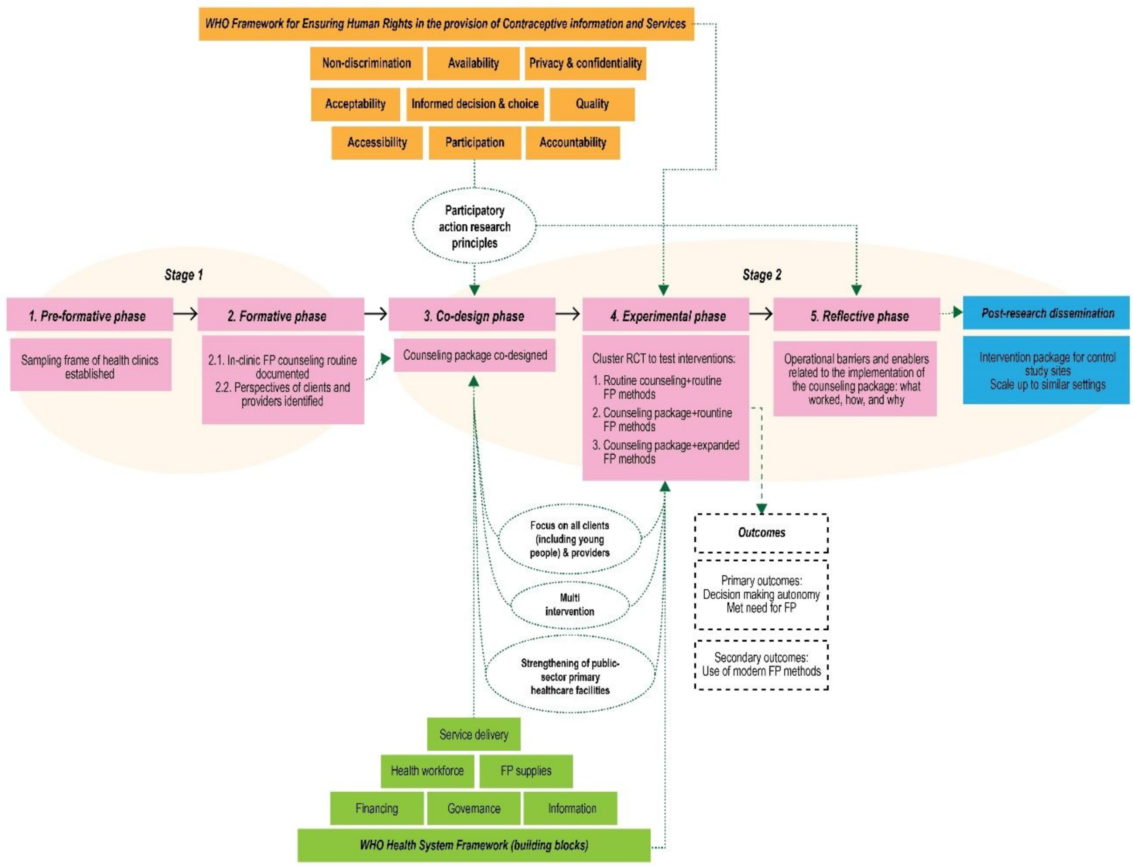

Figure 2. Flow diagram of the cluster randomized controlled trial (separately) in Pakistan and Nigeria.

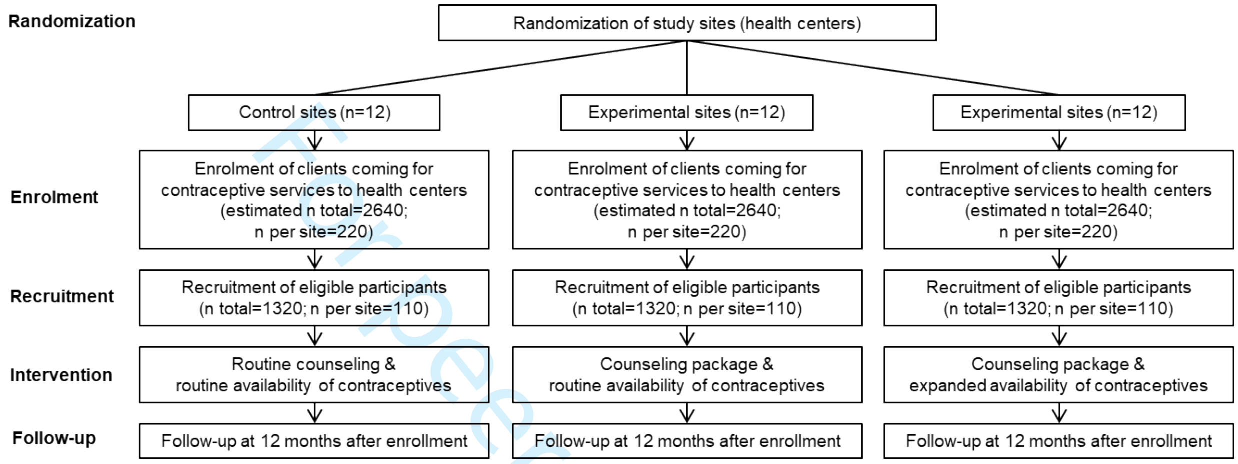

For peer review only

Figure 3. Schedule of enrolment, interventions, and assessments (SPIRIT template) for Pakistan and Nigeria (each country will use the same sampling framework)

	**Enrolment**	**Allocation**	**Post-allocation**			
**TIMEPOINT**	t_{-1} *Initial study family planning visit*	t_0 *Initial study family planning visit*	t_1 *After initial study visit*	t_2 *After each follow-up visit*	t_3 …	t_4 *12 months*
**ENROLMENT:**						
Eligibility screen (estimate)	7920					
Informed consent	3960					
Allocation		3960	X	X	X	X
**INTERVENTIONS & CONTROL:**						
Counseling package & routine availability of contraceptives		1320	◆—	—	—	—◆
Counseling package & expanded availability of contraceptives		1320	◆—	—	—	—◆
Control: routine counseling & routine availability of contraceptives		1320	◆—	—	—	—◆
**ASSESSMENTS:**						
**Baseline variables:** Met need for family planning; Modern contraceptive use		X				
**Primary outcomes:** Decision-making autonomy; Met need for family planning			X	X	X	X
**Secondary outcomes:** Modern contraceptive use			X	X	X	X

For peer review only

BMJ Open

Strengthening contraceptive counselling services to empower clients and meet their needs: protocol for a 2-stage, multi-phase complex intervention in Pakistan and Nigeria

Journal:	*BMJ Open*
Manuscript ID	bmjopen-2023-081967.R1
Article Type:	Protocol
Date Submitted by the Author:	15-Mar-2024

Complete List of Authors:	Tran, Nguyen Toan; University of Technology Sydney,<br>Ali, Moazzam; World Health Organization, Reproductive Health and Research<br>Azmat, Syed Khurram ; Jinnah Sindh Medical University, AAPNA - Institute of Public Health; Marie Stopes Society Pakistan, Technical Services<br>Seuc, Armando; World Health Organization, Department of Reproductive Health and Research<br>Olaolorun, Funmilola; University of Ibadan College of Medicine, Community Medicine<br>Awan, Muhammad Ali ; AAPNA Institute of Public Health (AIPH), Jinnah Sindh, Medical University, Rafiqui H.J, Iqbal Shaheed Rd, Karachi, Sindh 75510, Pakistan<br>Morhason-Bello, Imran; University of Ibadan College of Medicine, Department of Obstetrics and Gynaecology, Faculty of Clinical Sciences, College of Medicine/University College Hospital, University of Ibadan<br>Thom, Ellen Mpangananji ; World Health Organization Country Office in Islammabad Pakistan<br>Martin , Josepth ; World Health Organization Country Office in Abuja, Nigeria<br>Abubakar, Hanifah Datti ; Mohammed Abdullahi Wase Teaching Hospital, Nasarawa<br>Uzma, Qudsia ; World Health Organization Country Office in Islamabad Pakistan<br>Kiarie, James; World Health Organization, Department of Reproductive Health and Research

**Primary Subject Heading**:	Reproductive medicine
Secondary Subject Heading:	Health services research
Keywords:	Clinical Trial, Decision Making, Health Services, Public health < INFECTIOUS DISEASES

SCHOLARONE™
Manuscripts

Strengthening contraceptive counselling services to empower clients and meet their needs: protocol for a 2-stage, multi-phase complex intervention in Pakistan and Nigeria.

Tran Nguyen Toan[1,2,3] nguyentoan.tran@uts.edu.au
Moazzam Ali[1*] alimoa@who.int
Syed Khurram Azmat[4] khurramazmat@gmail.com
Armando Seuc[1] armandoseuc@infomed.sld.cu
Funmilola M. Olaolorun PI[5] fmolaolorun@gmail.com
Muhammad Ali Awan[4] alighous@gmail.com
Imran Morhason-Bello[6] imranmorhasonbello@gmail.com
Ellen Mpangananji Thom[7] thome@who.int
Josepth Martin[8] mjoseph@who.int
Hanifah Datti Abubakar[9] hanniedee83@gmail.com
Qudsia Uzma[7] uzmaq@who.int
James Kiarie[1] kiariej@who.int

*Corresponding author: alimoa@who.int

[1] Department of Reproductive Health Research, World Health Organization, Avenue Appia 20, 1211 Genève 27, Switzerland.

[2] Faculty of Medicine, University of Geneva, Rue Michel-Servet 1, 1206 Genève, Switzerland

[3]Australian Centre for Public and Population Health Research, Faculty of Health, University of Technology, PO Box 123, Sydney, NSW 2007, Australia

[4] APPNA Institute of Public Health (AIPH), Jinnah Sindh, Medical University, Rafiqui H.J, Iqbal Shaheed Rd, Karachi, Sindh 75510, Pakistan

[5] Department of Community Medicine, College of Medicine, University of Ibadan, Nigeria

[6]Department of Obstetrics and Gynaecology, College of Medicine, University of Ibadan, Nigeria

For peer review only - http://bmjopen.bmj.com/site/about/guidelines.xhtml

For peer review only

[7] World Health Organization Country Office in Islamabad Pakistan

[8] World Health Organization Country Office in Abuja, Nigeria

[9] Mohammed Abdullahi Wase Teaching Hospital, Nasarawa, Kano, Nigeria

Abstract

Background: High-quality contraceptive counseling can accelerate global efforts to reduce the unmet need for and suboptimal use of modern contraceptives. This study aims to identify a package of interventions designed to strengthen in Pakistan and Nigeria and determine their effectiveness in increasing client-level decision-making, autonomy and meeting of contraceptive needs.

Methods: A multi-site, two-stage and five-phase intervention design will start with a pre-formative, formative, design, experimental and reflective phase. The Pre-formative phase will map potential study sites and establish the sampling frame. The two-part formative phase will first use participatory approaches to identify clients' perspectives, including young couples and providers, to ensure research contextualization and address each interest group's needs and priorities followed by clinical observations of client-provider encounters to document routine care. The design workshop in the third phase will result in the development of a package of contraceptive counseling interventions. In the fourth experimental phase, a multi-intervention, three-arm, single-blinded, parallel cluster randomized-controlled trial will compare routine care (arm 1) with the contraceptive counseling package (arm 2) and the same package combined with wider method availability (arm 3). The study aims to enrol a total of 7920 participants. The reflective phase aims to identify implementation barriers and enablers. The outcomes are clients' level of decision-making autonomy and use of modern contraceptives.

Ethics and dissemination: Ethical approval for this study was obtained from the WHO Scientific and Ethics Review Committee (Protocol ID Pakistan: ERC 006232 and Nigeria ERC: 006523),). Each study site is required and has obtained the necessary ethical and regulatory approvals that are required in each specific country. Findings will be presented at local, national and international conferences and disseminated by peer-review publications.

Trial registration: Registered in ClinicalTrials.gov (NCT06081842, 31 October 2023).

Keywords

Health services research, counseling, contraception, family planning, empowerment, autonomy, qualitative research, cluster randomized controlled trial.

For peer review only

Strengths and limitations of this study

- The main strength of this study was the mixed methodology approach for example, our research will be using a qualitative study design followed by randomized controlled trial to assess the effectiveness of family planning counselling package.
- Inclusion of stakeholders at the start of research will enhance understanding of service provision dynamics and support efforts towards identifying strategies to enhance positive male involvement and engagement in women's sexual reproductive health and rights.
- The reflective phase will help identify operational barriers and enablers related to the new counselling package implementation and to optimize its utilization.
- The study will investigate the effect on outcomes i.e., clients' level of decision-making autonomy and use of modern contraceptives. Also the study will be implemented in a limited geography of the target countries; therefore, caution should be taken before generalising the findings.

Background

Overlooking women's and couples' need for quality contraceptive counseling constitutes a missed opportunity in family planning programs and services to ensure that every woman, man, and adolescent can enjoy the health, social, and economic benefits of modern contraception. Access to family planning is a human right enshrined in the United Nations' Sustainable Development Goal 3 on health and wellbeing and, specifically, its target 3.7: *ensure universal access to sexual and reproductive health-care services, including for family planning* [1]. Around 164 million women of reproductive age, have an unmet need for modern contraceptive methods despite increased contraceptive use over the past decades [2]. Satisfying the unmet need for contraception in low- and middle-income countries and offering all pregnant women and their newborns the standard care recommended by the World Health Organization (WHO) would result in dramatic reductions of unintended pregnancies by 68%, unsafe abortions by 72%, maternal deaths by 62%, and neonatal deaths by 69% [3]. Adequate contraceptive coverage among users can be improved by addressing suboptimal use and high discontinuation rates [4].

Effective contraceptive counseling can help individuals choose a method that meets their needs and preferences, manage any side effects, and continue using their preferred method or switch to an alternative one [5]. Contraceptive counseling could improve the effective use of modern contraception and reduce unmet need according to a systematic review that synthesized the evidence on the comparative effectiveness of different contraceptive counseling strategies [6]. The quality of contraceptive counseling in low-resource settings is often low. In Senegal, for example, only 18% of providers counseled their clients on the three-item Method Information Index (method use, side-effects, and timing of follow-up visits) [7]. Interventions targeting women requesting or initiating a chosen method, including structured counseling on side-effects, tend to show positive effects on contraceptive continuation [8-11]. Counseling interventions targeting family planning service users [12-16], abortion service users [17-19], postpartum women [20-25], and those reached by community-based interventions [26-28] have inconsistent effects on

For peer review only - http://bmjopen.bmj.com/site/about/guidelines.xhtml

contraceptive behavior and satisfaction outcomes. In contrast, increased contraceptive use often follows interventions introducing systematic contraceptive counseling among women attending non-family planning outpatient services [29-31]. However, these interventions often include expanded contraceptive method provision, and the observed changes cannot be attributed to counseling alone. High-quality evidence is generally limited, and no clear consensus exists on how best to deliver contraceptive counseling to meet clients' contraceptive needs and enhance their satisfaction.

Building on this systematic review, the World Health Organization (WHO) organized a geographically diverse think tank in May 2019 in Geneva, Switzerland, to identify research and guidance gaps and other opportunities to improve the quality of contraceptive counseling [32]. Equally important was the crafting of a definition on contraceptive counseling, which was adopted by think tank members [33]: *Contraceptive counseling is defined as the exchange of information on contraceptive methods based on an assessment of the client's needs, preferences and lifestyle to support decision-making as per the client's intentions. This includes the selection, discontinuation or switching of a contraceptive method. The key principles are based on coercion-free and informed choice; neutral, understandable and evidence-based information; collaborative and confidential decision-making process; ensuring respectful care, dignity and choice.* The definition took into consideration the core dimensions articulated in the literature and mirrors the principles that were deliberated by the think tank: a two-way discussion and decision-making process between the client and provider, sharing objective and user-friendly information, and fundamentally grounded in the rights to autonomy, dignity, privacy, respect, and participation, among others. In addition, person-centeredness is a critical component of quality in family planning [5]. The 4-item person-centered contraceptive counselling scale (PCCC) is a valid and reliable measure of person-centered contraceptive counseling that reflects patients' perspectives on contraceptive counseling [34]. For example, integrating Dehlendorf's 4-item validated tool: PCCC: person-centered contraceptive counselling scale: Think about your visit. How do you think the member of the health care team did? Please rate them on each of the following by circling a number (the scale will use – Poor, Fair, Good, Very good and Excellent). Hence, to measure decision-making autonomy, the following considerations need to factor:

- Respecting me as a person
- Letting me say what mattered to me about my birth control method
- Taking my preferences about my birth control seriously
- Giving me enough information to make the best decision about my birth control method

Furthermore, the think tank members agreed on a prioritized set of guidance, knowledge, and implementation research gaps [32]. Such gaps include, for example, understanding or identifying clients' experiences with and expectations of counseling in a variety of global settings; the types of settings different populations, particularly adolescents, unmarried women, migrants, gender non-conforming people, and religious/ethnic minorities, receive most of their contraceptive counseling, and the quality of counseling across these settings; the fluidity/ambivalence of pregnancy intentions, risk perception (pregnancy and sexually transmitted infections), and women's understanding of their bodies; the independent effect of counseling from method

For peer review only - http://bmjopen.bmj.com/site/about/guidelines.xhtml

provision; the core elements of high-quality of contraceptive counseling and how these differ by context and what provider characteristics affect counseling quality; the minimum skills and competencies for contraceptive counselors; the contents of a comprehensive package of best practices for FP counseling for different populations.

We have reviewed and used the currently available resources noted above such as Person-centered care and effective contraceptive counseling approaches, which focuses on persons' values and preferences, and are considered to improve the quality of care in family planning. These approaches are likely to promote greater patient autonomy, trust, and satisfaction with services. Majority of the research has been based on small, qualitative studies, this study, using mixed methods approach, will benefit from existing resources and have also highlight the rights based approach in the counselling package to understand and document its implementation and effectiveness in the context of two countries.

Study overall objective and hypothesis

Owing to these gaps combined with the socio-cultural challenges related to accessing contraception, we propose a multi-site, two-stage and 5-phase study with two main objectives. The objective is to identify and test the effectiveness of a package of contraceptive counseling interventions on improving clients' informed decision-making autonomy as well as meeting their need for family planning. The outcomes are clients' level of decision-making autonomy and use of modern contraceptives.

The research rests on the working hypotheses that the package will strengthen existing contraceptive counseling services; account for the views of key actors, including clients and providers; be in line with national health policies and WHO global standards; and take into consideration the scarcity of public resources.

Study countries: Pakistan and Nigeria

Pakistan

The study will be implemented in Pakistan in partnership with the Ministry of National Health Services, Regulations and Coordination. The overall demographic, family planning, and reproductive health indicators based on the 2017-18 Demographic and Health Survey [35] note that rural settings fared worse than urban settings. The total fertility rate was 3.6 children per woman, modern contraceptive prevalance rate was 25% , overall unmet need for family planning was 17% and demand for family planning satisfied by a modern method 49%. In terms of family planning knowledge, more than 98% of urban and rural women had heard about any type of modern methods—before marriage, only 15% of women did. Regarding men's attitudes toward contraceptive use, 27% believed that family planning use is a woman's business, while 16% thought it may encourage women to be promiscuous [36].

For peer review only - http://bmjopen.bmj.com/site/about/guidelines.xhtml

Nigeria

The study will also be implemented in Nigeria by College of Medicine, University of Ibadan. The study will be conducted in two study sites: Lagos (southern part of Nigeria) and Kano (northern part of Nigeria) states. These states were selected for the following reasons: (1) Data from the Performance Monitoring for Action (PMA) program are available for both states from annual surveys over the past three years, including information on the method information index plus and other family planning indicators (pmadata.org); (2) The team has a good network in both locations and will be able to readily collaborate with the State Ministries of Health; (3) Safety and security are relatively good with no serious threats in recent times.

The latest demographic, family planning, and reproductive health indicators based on the 2018 Demographic and Health Survey in Nigeria showed that in general, rural settings fared worse than urban settings [37]. The total fertility rate was 5.3 children per woman; overall unmet need for family planning was 18.9%; demand for family planning satisfied by a modern method 33.9% and modern contraceptive prevalance rate was 12%. In terms of family planning knowledge, 93.9% of currently married women and 98.3% of sexually active unmarried women had heard about any type of modern contraceptive method.

Methods

Framework and perspectives

Theory of change

To ensure that the contraceptive counseling intervention package that will emerge from this trial strengthens existing family planning services and is effective, safe, and culturally appropriate, this health services research is informed by the theory of change framework as well as perspectives on human rights and the health system. Following the research findings, the long-term vision is to support the health system in developing a sustainable and scalable contraceptive counselling intervention package for Nigeria and Pakistan. Although the exact package has not yet been developed, based on the requirement set by the respective ministry of health/population in Pakistan and Nigeria, the researchers are willing to contribute to the development of provider training, post-training mentorship and the provision of IEC materials to enable the implementation of the intervention package.

The staged and phased approach adopted in this study, with the different specific objectives, levels of activities, and intermediate and final results (see Figure 1), is underpinned by the theory of change [38].

For peer review only

For peer review only - http://bmjopen.bmj.com/site/about/guidelines.xhtml

FIGURE 1 HERE

Health system perspective

First and related to the research model outlined in Figure 1, the study will rely on the WHO Health System Framework with its six system building blocks and link research and programmatic considerations for contraceptive counseling [39]. The six system building blocks are leadership/governance, health care financing, health workforce, medical products and technologies, information and research, and service delivery [39].

Human rights perspective

The research will use the WHO Framework for Ensuring Human Rights in the Provision of Contraceptive Information and Services [40]. The human rights framework comprises nine standards that interweave with the dimensions of quality of care: non-discrimination, availability, accessibility, acceptability, quality, informed-decision making, protection of privacy and confidentiality, participation, and accountability. The research team would ensure a person-oriented approach where no women or teenage girls are discriminated against (for example, in the provision of family planning information and services) because of their age, marital status, HIV status, minority status, or other medical or social factors [33].

With regard to the participation standard, the research will ensure active and informed participation of individuals in decision-making that affects them, including on matters related to their health [41]. Due to the complex social, cultural, and religious challenges and opportunities surrounding contraceptive use, our research design will assume that the following participatory action research (or participatory research) principles will be essential to meet the study objectives as well as gain insights into the implementation process. [42].

Study phases and timelines

The research entails a complex multi-site, two-stage and 5-phase (Figure 1): STAGE 1 is formative and will comprise of a pre-formative (phase 1) and a formative (phase 2). While the STAGE 2 is experimental and will include intervention design (phase 3), experimental (phase 4), and reflective (phase 5). The proposed methods of each phase are not intrinsically complex—the overall intervention is complex as it features a *number and difficulty of behaviors required by those delivering or receiving the intervention* and involves a *number of groups or organizational levels targeted by the intervention*, among others [43]. The design follows the recommendations from the Medical Research Council (2008/2010) to develop a complex intervention, determine its feasibility, and facilitate its implementation and evaluation [43]. Therefore, the phases are interconnected and have specific objectives, methodologies, and results.

STAGE 1: The specific objective of the pre-formative phase is to establish a sampling frame of potential study sites by mapping primary health centers according to pre-established

For peer review only - http://bmjopen.bmj.com/site/about/guidelines.xhtml

criteria (both public and private facilities). Health centers are eligible if they (1) provide family planning services; (2) offer at least between three and four modern contraceptive methods, including a barrier method such as condom, and a short-term method such as pills or injectables, a long-term method such as implants, intra-uterine device, and referrals for permanent methods to clients; (3) have no stock-outs of all contraceptive methods during the preceding three months as per the national policy; (4) have on average at least 25 family planning encounters per week or 100 family planning encounters per months at a selected high-performing facility (each of public and private sector) and have on average at least 12 FP encounters per week or 48 FP encounters per months at a selected low-performing facility (each of public and private sector); (5) are not recipients of technical support for family planning (capacity building, supplies) from other organizations at the time of recruitment and commit not to receive any such support during the whole study; and (6) are willing to participate.. Based on this sampling frame, four health centers (public and private sector) will be allocated to the formative phase while the others will be randomized to each arm of the experimental phase. The specific objectives of the ***formative phase*** are to (1) document the in-clinic contraceptive counseling routine using observation checklists and (2) seek through qualitative research the perspectives of clients, including young people, providers, and other stakeholders to ensure adequate contextualization of the research and address needs and priorities of each interest group regarding contraceptive counseling barriers and enablers.

STAGE 2: The specific objective of the ***intervention design phase*** is to co-design, based on the results of the formative phase and through a participatory and consensus-building approach engaging clients, providers, and other main actors, a package of contraceptive counseling to be implemented during the experimental phase. To be integrated into the final package, interventions will need to consider current human resources and clinical capacity, costs, acceptability, feasibility, scalability, and other field considerations.

The specific objective of the ***experimental phase*** is to determine, through a cluster randomized controlled trial (RCT) design, the effect of the contraceptive counseling package on clients' level of decision-making autonomy and demand satisfied for family planning. The three-armed cluster RCT comprise (1) a control arm with contraceptive counseling and care and a range of family planning methods that are available at field level per the national policy and guidelines; (2) an experimental arm implementing the contraceptive counseling package but still with a range of family planning methods that are available (upto 4 methods) per the national policy and guidelines at field level; and (3) another experimental arm with the contraceptive counseling package but with an expanded range of family planning methods (more than 4 methods) that are recommended by national policy and guidance.

The specific objective of the ***reflective phase*** is to identify operational barriers and enablers related to the package implementation using qualitative research. Once the package of interventions has been identified—and resources permitting, we might consider conducting a cost-effectiveness analysis to determine the counseling package cost-effectiveness.

The qualitative research and cluster RCT are detailed in the following sections.

Timelines

For peer review only - http://bmjopen.bmj.com/site/about/guidelines.xhtml

The study be tenataively initiated in November 2023 and it will be completed by November 2025. The analysis and report will be drafted by June 2026.

Qualitative research

General design

For the qualitative components of the study (in the formative and reflective phases), we will conduct in-depth interviews (IDIs) in Pakistan and Nigeria with members of the community (women , adolescent males and females, partners, people with influence), health staff (service providers, managers, Community Health Workers - CHWs), development partners, donor agencies, and national policymakers. The formative phase will involve four (4) conveniently sampled health centers (public and private) and their respective coverage areas in each country. These sites will be selected from the sampling frame determined in the pre-formative phase.

For the identification of contraceptive counselling barriers and enablers, separate IDIs of six to ten (6-10) participants will be conducted for the following categories of individuals (1) women who have used family planning services at the health center in the previous 12 months–: married and unmarried adolescent females (≤ 19 years), married and unmarried women 19-39 years, married and unmarried women ≥ 40 years [The study will include Women of Reproductive Age (WRA) - irrespective of their marital status and age group (including migrants refugee people, ethnic and religious minorities, women or men with disabilities, women or men with HIV etc. are inclusive] ; (2) married and unmarried men who are partners or husbands of women who have used family planning services at the health center in the previous 12 months, and married and unmarried adolescent males (<19 years); and (3) family planning service providers.

About the recruitment of men/male participants, the women participants will be first recruited based on their eligibility and recruitment criteria. If the female participant is selected, she will be asked whether she is using the FP method in consensus with her male partner/spouse or using it covertly. If she uses the FP method with the consensus of her male partner/spouse, we will recruit the accompanying male partners/spouses in the study. If the women participant is using the FP method covertly, then the women will be first asked to provide consent to be invited to study participation. If she does not consent, then men/male participants will be identified by the CHWs at the community level. In both cases, formal consent will be taken from the woman whether she is using the FP method covertly or in consensus with her male partner/spouse.

IDIs will also take place with clinic managers, supervisors, CHWs, policy makers, and other influential individuals/opinion leaders (selected purposively) who have observed, supervised, or monitored family planning services at/or the catchment of the selected public/private health facility. We anticipate that interviews will last between 45-60 mins and will take place in a safe, neutral space of the participant’s choosing (e.g. town hall, office space, on porch outside participant’s home, etc.). Both the recruiter and the participant will decide and agree on the location of the interview and this will be conveyed to the research team. Alternatively, the team will also identify pay-per-hour spaces as an option to present to the participant. The interview guides will cover the following themes related to contraceptive counselling: knowledge, attitudes, and practices; socio-cultural and health systems barriers and catalysts; and quality of care, including autonomy, informed decision-making, and other human rights considerations.

For peer review only - http://bmjopen.bmj.com/site/about/guidelines.xhtml

The qualitative research in the ***reflective phase*** will occur in three health centers in each of the three study arms (nine centers in total). The centers will be purposely sampled to include the lowest, average, and highest performing ones in terms of measured outcomes to enable the broadest possible understanding. In line with the theory of change approach, the qualitative research will consist of an implementation evaluation to analyze barriers and enablers related to the delivery of contraceptive counseling and family planning services, assess intervention fidelity (in the experimental groups), and explore approaches for scaling up. Separate IDIs are planned for service providers and clients (≤ 19 years, 20-39 years, ≥ 40 years). IDIs are planned for clinic managers. The interview guides will cover the following themes related to contraceptive counseling: operational barriers and enablers; quality of care, including decision-making autonomy and other human rights perspectives; and scaling-up considerations.

Selection criteria and recruitment approach: A standard process of selection and consent-taking will be undertaken at each study site comprising potential study participants for each of the above categories of individuals (including vulnerable populations). A recruitment approach will follow, and the main actors who will facilitate the recruitment process of the potential target respondents for this research are a) selected service providers, b) Community Health Workers – working in the catchment area of the selected service providers, c) community-based opinion leaders, i.e., male, and female schoolteachers, social activist/ NGO workers. The country PI/Co-PI will train all of them on the study's main objectives, purpose, issues in ethics and the importance of consent.

Qualitative data management and analysis

At the country level, the study will engage a team of reseachers with relevant experience in qualitative methods and in working in local languages.

The interviews will occur in a location guaranteeing privacy. We anticipate that interviews will last between 45-60 mins and will take place in a safe, neutral space of the participant's choosing (e.g. town hall, office space, on porch outside participant's home, etc.). The interviews will be audiotaped after obtaining informed conent from participants. Research assistants with experience in transcription will transcribe and translate the audio records into English. Accuracy checks will be done by comparing transcripts with audio files.

Qualitative research management Softwares such as QSR NVivo 12 or Atlas.ti , , will be used to perform a thematic analysis by establishing a basic codebook describing all the nodes and using it to code data. The codebook will be enriched with new emerging nodes during the coding process. Data will be coded by research assistants competent in NVivo. To ensure the quality of data coding, inter-rater reliability will be tested by computing the Cohen's Kappa coefficient with a Kappa coefficient of 0.8 and above as an acceptable concordance threshold.

The formative phase results will be presented and discussed during the intervention design workshop with key members of the research team, selected service providers, and clients with a view to informing the bottom-up design of a contextually sensitive package of contraceptive counseling interventions. The views of each workshop participant will be considered when examining the potential effectiveness of possible interventions, acceptability to

For peer review only - http://bmjopen.bmj.com/site/about/guidelines.xhtml

women and providers, feasibility, the potential for integration into the overall family planning programs, sustainability, and scalability.

Cluster randomized controlled trial

The research will be a multi-intervention, three-arm, single-blinded, parallel, cluster RCT done in selected primary health centers (clusters) in Pakistan and in Nigeria (see **Figure 2** for the cluster RCT flow diagram, and **Figure 3** for the schedule of enrolment, interventions, and assessments (SPIRIT template). Centers will be randomly allocated to the three study arms in matched ratios (1:1:1) on the basis of the number of monthly family planning encounters, the number of available contraceptive types, the ratio of health workers per population in the clinic coverage zone, the location in urban or rural settings, and selected district-level variables that may have an influence on the study outcomes, including the unmet need for family planning, the level of literacy among women, and household income terciles. Only data analysts will be masked/blinded to trial-arm allocation. Service providers skilled in family planning services will implement the clinical components of the contraceptive counseling package.

FIGURE 2 HERE

FIGURE 3 HERE

Outcome measures

Quantitative indicators will be captured in case-report forms (CRFs) developed and piloted at the country level before finalization. The proposed outcomes to be assessed after each visit, at mid-point (6 months after enrollment), and at the study end (12 months after enrollment) are oriented toward clients and consist of levels of clients' decision-making autonomy and demand satisfied for family planning.

To determine their level of decision-making autonomy, clients will be asked to answer questions about the different domains contained in the counseling definition (see Background). These domains comprise client-provider collaboration, client's needs and preferences, the quality of information, freedom from coercion, confidentiality, and dignity. The Person-Centered Contraceptive Counseling scale (PCCC), developed and tested in publicly-funded clinics in California will also be used [44]. The four PCCC questions fall under the domains of respect, eliciting—and giving serious consideration to—client's preferences, and information quality and it will be assessed during the intervention phase as per described by UNFPA to measure decision-making autonomy SDG 5.6.1 [34].

The study proposes nonetheless the another outcome, while acknowledging its limitations: modern contraceptive prevalence after the first visit and at 6 and 12 months after enrollment.

For peer review only - http://bmjopen.bmj.com/site/about/guidelines.xhtml

Eligibility criteria for clusters (health centers) and participants

The study will be conducted in selected primary health centers. The eligibility criteria of health centers were described above under the objective of the pre-formative phase. A woman will be eligible to participate in the study if

(1) she comes to the family planning clinic with the intention to (i) use contraception for the first time in her life (new user), or (ii) switch from a contraceptive method to another one (switching user), or (iii) resume a method after not using any in the prior three months (lapse user), or (iv) discontinue a modern method (discontinuing user);

(2) she is not coming for the resupply of a currently used method, such as pills or injectables;

(3) she has the intention to continue her follow-up at the health center during the 12-month study follow-up;

(4) she does not participate in another study; and

(5) she provides informed consent.

Exclusion Criteria: Not willing to participate in the complete duration of the trial.

Interventions

The health centers of the first experimental group will implement the intervention package, and those of the second experimental group will implement the intervention package in addition to an expanded range of family planning methods that are recommended by national guidance. The control group will implement usual care. Depending on the nature of the final package, some interventions may be implemented before participant recruitment, such as the capacity building of providers.

Randomization

In each country, the 36 sites will be matched on a 1:1:1 ratio according to the above-described criteria. Within each of the 12 trios, study sites will be randomly assigned to each of the three study arms. This randomization is performed 12 times (once for each trio). No restriction in the randomization process is required and masking cannot be done except for data analysts. All consecutive and eligible participants will be included in the clusters until the sample size of 7920 is achieved.

For peer review only - http://bmjopen.bmj.com/site/about/guidelines.xhtml

Data source and collection

Data on key outcome measures as well as quantitative process indicators will be collected on paper-based/electronic CRFs. The WHO team in Geneva will develop the CRFs with inputs from the country research teams. We will test advanced drafts with an appropriate sample of service providers and mock clients from the formative phase sites before finalizing the forms. Each health center of the cluster RCT will have a research assistant who will be trained to adhere to the study manual and standard operating procedures for data management which are common to both study countries and have been developed by WHO. The information reported on the CRFs will be checked for accuracy and completeness several times at different levels by field coordinators and data managers.

Data management

As for other multicenter studies conducted and sponsored by the UNDP/UNFPA/UNICEF/WHO/World Bank Special Programme of Research, Development and Research Training in Human Reproduction (HRP) at WHO in Geneva, data entry and data quality monitoring will be carried out using a web-based data management system. It will be developed by HRP's Biostatistics and Data Management team using OpenClinica (version 3.16), an electronic data capture software for clinical research that includes programmed edit checks for data quality, completeness, and consistency reduces delays in data queries and problem resolution and allows the dataset to be easily available. The OpenClinica system is a highly secure, open-source, and trusted web-based software solution that is fully compliant with Good Clinical Practice (GCP) and regulatory guidelines and HRP/WHO Standard Operating Procedures for clinical trial management.

The country research teams in Pakistan and Nigeria will enter the data locally and have it reviewed by the WHO team in Geneva. We will train the country teams in data entry and data management through OpenClinica system. Country research teams will be responsible for verifying the data, uploading it onto shared files, and entering the data into the OpenClinica system. Double data entry will be done to minimize errors. The system can produce automatic data queries (missing values, outliers, inconsistencies, and other errors). Data quality will be monitored by the WHO team. Questions regarding data inconsistencies or missing values will be submitted to sites via standardized forms and solved on a continuous basis. To ensure data integrity and participant confidentiality, data transmission will be encrypted. Only approved users will be given access to the data, which will be password protected.

Statistical analysis

We will prepare a comprehensive statistical analysis plan. Participants will form the unit of analysis and the clustered nature of the data will be accounted for. All analyses will be by intention to treat. For continuous variables, we will compute means, standard deviations, minimum and maximum values, and for categorical variables, we will compute frequencies and percentages. We will look at the distribution of variables and see if there are any outliers as part of data quality control and descriptive analysis. Descriptive statistics will be tabulated for individual clusters and aggregated across clusters. Within each of the three groups/arms of the study (two experimental and one control), outcome differences between final (12 months after

For peer review only - http://bmjopen.bmj.com/site/about/guidelines.xhtml

enrolment) and intermediate (6 months after enrolment) outcome measurements will be obtained, and these changes will be compared between the control group and each of the two experimental groups. For categorical outcomes, chi-square tests for two independent proportions will be used, and for continuous variables, t-student tests for two independent means will be used. We will use IBM SPSS Statistics 25, R 3.6.2, STATA 16.1, or SAS 9.4 statistical packages for the analysis. The findings will be presented using the CONSORT Statement checklist from 2010 and its extension for cluster studies [45].

Patient and public involvement

Patients or the public were not involved in the design, or conduct, or reporting, or dissemination plans of this research.

Ethics and regulatory aspects

We followed the guidelines in The Ottawa Statement on the Ethical Design and Conduct of Cluster Randomized Trials while designing this experiment [46]. The Research Project Review Panel (RP2) of the HRP peer-reviewed the protocol, and RP2 and the WHO Research Ethics Review Committee, Geneva, Switzerland (Pakistan: ERC 006232 and Nigeria ERC: 006523), approved it and so did the IRB of Research and Development Solutions (IRB No:00010843) in Pakistan. While the Ministry of Health, Kano State of Nigeria (NHREC 17/03/2018) and Lagos Research Ethics Committee in Nigeria (LREC/06/10/2180) provided ethical approvals for the Nigeria study .

This research is grounded in human rights principles, specifically participation in the form of participatory action research. The existing literature shows promise that our proposed approach may be successful. For example, a synthesis of the evidence on community-based participatory research in different settings indicates that collaboration among community partners, researchers, and organizations led to community-level action that enhanced health and wellbeing while minimizing health disparities [47]. In the process, it also strengthened the capacity of the community in terms of research and leadership skills. Another review found that participatory action research could ensure that research is appropriate culturally and logistically; enhance recruitment capacity; generate professional capacity and competence in stakeholder groups; result in productive conflicts followed by useful negotiation; increase the quality of outputs and outcomes over time; increase the sustainability of project goals beyond funded time frames and during gaps in external funding; and create system changes and new unanticipated projects and activities [48].

Dissemination

The findings from the analysis of this study will be disseminated in a variety of ways. At country level, the results will be presented to policy makers, researchers, managers through policy briefs and workshops in collaboration with the WHO office. The results will also be disseminated among the communities/participants through online platforms/text messages, which will be provided to the participants by the data collectors/health workers. In addition, the results will also be presented to the scientific and funding community in collaboration with WHO

For peer review only - http://bmjopen.bmj.com/site/about/guidelines.xhtml

country and regional offices, by communication and manuscript publications in peer-reviewed international journals.

Discussion

Ensuring access to contraception is fundamental to human rights and contributes to improved health outcomes, as recognised in Sustainable Development Goal 3.7 (universal access to sexual and reproductive healthcare services).

Quality contraceptive information and services reinforce people's freedom to determine the number and spacing of their children and offer a range of potential benefits encompassing women's empowerment, economic development, education and improved health outcomes, including maternal and child health.

Contraceptive counselling can help clients choose a method meeting their needs and preferences, manage side effects, and support method continuation or switching. High-quality counselling therefore has a high potential to strengthen efforts to reduce unmet need for contraception.

The results and insights gleaned from this study will provide a better understanding of the counselling strategies to improve contraceptive use, increased satisfaction and decrease discontuination of the contraceptive methods leading to achievement of global targets in reprorucitve health.

Declarations

Ethics approval and consent to participate

The research protocol was approved by the Research Project Review Panel (RP2) of the UNDP/UNFPA/UNICEF/WHO/World Bank Special Programme of Research, Development and Research Training in Human Reproduction (HRP) at WHO in Geneva, and the WHO Research Ethics Review Committee, Geneva, Switzerland. The study was approved by the ethics review committee in Pakistan and Nigeria. All adult participants will provide written consent. Non-adult participants will give their informed assent and their respective parents or guardians will provide their informed consent.

Consent for publication:

Not applicable.

Availability of data and materials

Not applicable.

For peer review only - http://bmjopen.bmj.com/site/about/guidelines.xhtml

Competing interests

The authors declare that they have no competing interests.

Patient and public involvement Patients and/or the public were not involved in the design, or conduct, or reporting, or dissemination plans of this research.

Funding

The project site in Pakistan has been supported by the UNDP-UNICEF-WHO-World Bank Special Programme of Research, Development and Research Training in Human Reproduction (HRP); a co-sponsored programme executed by the World Health Organisation (WHO). The project in Nigeria generously received funding from United States Agency for International Development (USAID grant No. GHA-G-00-09-00003). The funders will take no role in subjects' recruitment, data collection, analyses, or interpretation, and will take no part in the decision to publish.

Authors' contributions

MA, TNT and AS designed the initial study concept, the study protocol, and study instruments. SKA,MA, MAA and FMO provided revisions to multiple versions of the study protocol based on the feedback from WHO-ERC. SKA, MA, FMO, MAA, EMT, JM, QU, JK, HDA and IMB contributed to the final study protocol and study instruments. TNT wrote the first draft of the manuscript with the contributions of MA, AS, and KJ. While, SKA and MA developed the final draft of the manuscript. All authors read and approved the final manuscript.

Acknowledgements

The authors are grateful to Dr. Onikepe Owolabi of the Guttmacher Institute and Dr. Nathalie Roos of the Karolinska Institute for their technical support and insights.

ORCID:

Moazzam Ali http://orcid.org/0000-0001-6949-8976

Syed Khurram Azmat https://orcid.org/0000-0003-0158-8734

References

1. Howden-Chapman P, Siri J, Chisholm E, Chapman R, Doll CN, Capon A: **SDG 3: Ensure healthy lives and promote wellbeing for all at all ages**. *A guide to SDG interactions: from science to implementation Paris, France: International Council for Science* 2017:81-126.

For peer review only - http://bmjopen.bmj.com/site/about/guidelines.xhtml

2. United Nations Department of Economic and Social Affairs, Population Division (2022). World Family Planning 2022: Meeting the changing needs for family planning: Contraceptive use by age and method.UN DESA/POP/2022/TR/NO. 4

3. Sully E, Biddlecom A, Darroch J, Ashford L, Lince-Deroche N, Riley T: **Adding It Up: Investing in Sexual and Reproductive Health, 2020**. *New York, NY: Guttmacher Institute*.

4. Polis CB, Bradley SE, Bankole A, Onda T, Croft T, Singh S: **Contraceptive failure rates in the developing world: an analysis of demographic and health survey data in 43 countries**. *New York: Guttmacher Institute* 2016.

5. Dehlendorf C, Krajewski C, Borrero S: **Contraceptive counseling: best practices to ensure quality communication and enable effective contraceptive use**. *Clinical obstetrics and gynecology* 2014, **57**(4):659.

6. Cavallaro FL, Benova L, Owolabi OO, Ali M: **A systematic review of the effectiveness of counselling strategies for modern contraceptive methods: what works and what doesn't?** *BMJ Sexual & Reproductive Health* 2019.

7. Assaf S, Wang W, Mallick L: **Provider Counseling and Knowledge Transfer in Health Facilities of Haiti, Malawi, and Senegal. DHS Analytical Studies No. 60**. *Rockville, Maryland* 2016.

8. De Cetina TEC, Canto P, Luna MO: **Effect of counseling to improve compliance in Mexican women receiving depot-medroxyprogesterone acetate**. *Contraception* 2001, **63**(3):143-146.

9. Modesto W, Bahamondes MV, Bahamondes L: **A randomized clinical trial of the effect of intensive versus non-intensive counselling on discontinuation rates due to bleeding disturbances of three long-acting reversible contraceptives**. *Hum Reprod* 2014, **29**(7):1393-1399.

10. Patel D, Koenig M, Patel A, Rani M: **The impact of improved service quality on IUD continuation: evidence from rural India**. *JOURNAL OF FAMILY WELFARE* 2003, **49**:1-9.

11. Amatya R, Akhter H, McMahan J, Williamson N, Gates D, Ahmed Y: **The effect of husband counseling on NORPLANT® contraceptive acceptability in Bangladesh**. *Contraception* 1994, **50**(3):263-273.

12. Jain AK, RamaRao S, Kim J, Costello M: **Evaluation of an intervention to improve quality of care in family planning programme in the Philippines**. *Journal of biosocial science* 2012, **44**(1):27.

13. Chin-Quee DS, Janowitz B, Otterness C: **Counseling tools alone do not improve method continuation: further evidence from the decision-making tool for family planning clients and providers in Nicaragua**. *Contraception* 2007, **76**(5):377-382.

14. Sanogo D, RamaRao S, Jones H, N'diaye P, M'bow B, Diop CB: **Improving quality of care and use of contraceptives in Senegal**. *African journal of reproductive health* 2003:57-73.

15. Sapkota S, Rajbhandary R, Lohani S: **The impact of balanced counseling on contraceptive method choice and determinants of long acting and reversible contraceptive continuation in Nepal**. *Maternal and child health journal* 2017, **21**(9):1713-1723.

16. Kim YM, Putjuk F, Basuki E, Kols A: **Increasing patient participation in reproductive health consultations: an evaluation of "Smart Patient" coaching in Indonesia**. *Patient education and counseling* 2003, **50**(2):113-122.

17. Zhu JL, Zhang W-H, Cheng Y, Xu J, Xu X, Gibson D, Støvring H, Claeys P, Temmerman M: **Impact of post-abortion family planning services on contraceptive use and abortion rate among young women in China: a cluster randomised trial**. *The European Journal of Contraception & Reproductive Health Care* 2009, **14**(1):46-54.

18. Ferreira ALCG, Souza AI, Pessoa RE, Braga C: **The effectiveness of contraceptive counseling for women in the postabortion period: an intervention study**. *Contraception* 2011, **84**(4):377-383.

19. Smith C, Ngo TD, Gold J, Edwards P, Vannak U, Sokhey L, Machiyama K, Slaymaker E, Warnock R, McCarthy O: **Effect of a mobile phone-based intervention on post-abortion contraception: a**

For peer review only - http://bmjopen.bmj.com/site/about/guidelines.xhtml

randomized controlled trial in Cambodia. *Bulletin of the World Health Organization* 2015, **93**:842-850A.

20. Kaewkiattikun K: **Effects of immediate postpartum contraceptive counseling on long-acting reversible contraceptive use in adolescents**. *Adolescent health, medicine and therapeutics* 2017, **8**:115.
21. Bolam A, Manandhar DS, Shrestha P, Ellis M, Anthony MdL: **The effects of postnatal health education for mothers on infant care and family planning practices in Nepal: a randomised controlled trial**. *Bmj* 1998, **316**(7134):805-811.
22. Adanikin AI, Onwudiegwu U, Loto OM: **Influence of multiple antenatal counselling sessions on modern contraceptive uptake in Nigeria**. *Eur J Contracept Reprod Health Care* 2013, **18**(5):381-387.
23. Vural F, Vural B, Cakiroglu Y: **The effect of combined antenatal and postnatal counselling on postpartum modern contraceptive use: prospective case-control study in Kocaeli, Turkey**. *Journal of clinical and diagnostic research: JCDR* 2016, **10**(4):QC04.
24. Fatima P, Antora AH, Dewan F, Nash S, Sethi M: **Impact of contraceptive counselling training among counsellors participating in the FIGO postpartum intrauterine device initiative in Bangladesh**. *International Journal of Gynecology & Obstetrics* 2018, **143**:49-55.
25. Ndegwa SW, Gichuhi J, Qureshi Z, Lubano K: **The effect of two levels of counselling on acceptance, uptake and early outcomes of post-placental intra-uterine contraceptive device**. *East African medical journal* 2014, **91**(12):449-456.
26. Terefe A, Larson CP: **Modern contraception use in Ethiopia: does involving husbands make a difference?** *American Journal of Public Health* 1993, **83**(11):1567-1571.
27. El-Khoury M, Thornton R, Chatterji M, Kamhawi S, Sloane P, Halassa M: **Counseling women and couples on family planning: a randomized study in Jordan**. *Studies in family planning* 2016, **47**(3):222-238.
28. Lemani C, Tang JH, Kopp D, Phiri B, Kumvula C, Chikosi L, Mwale M, Rosenberg NE: **Contraceptive uptake after training community health workers in couples counseling: A cluster randomized trial**. *PloS one* 2017, **12**(4):e0175879.
29. Gillespie D, Bradley H, Woldegiorgis M, Kidanu A, Karklins S: **Integrating family planning into Ethiopian voluntary testing and counselling programmes**. *Bulletin of the World Health Organization* 2009, **87**:866-870.
30. Lee J, Papic M, Baldauf E, Updike G, Schwarz EB: **A checklist approach to caring for women seeking pregnancy testing: effects on contraceptive knowledge and use**. *Contraception* 2015, **91**(2):143-149.
31. Yassin AS, Cordwell D: **Does dedicated pre-abortion contraception counselling help to improve post-abortion contraception uptake?** *BMJ Sexual & Reproductive Health* 2005, **31**(2):115-116.
32. Ali M, Tran NT, Kabra R, Kiare J: **Strengthening contraceptive counselling: gaps in knowledge and implementation research**. In.: British Medical Journal Publishing Group; 2021.
33. Ali M, Tran NT: **Defining counselling in contraceptive information and services: outcomes from an expert think tank**. In.: British Medical Journal Publishing Group; 2021.
34. UNFPA and Hera (2019). Research on what determines women's ability to decide on their SRHR and the relationship between this and other aspects of their lives. Volumes 1 and 2: Final report, October 2019. https://www.unfpa.org/sites/default/files/resource-pdf/20-033_SDG561-IssueBrief-v4.1.pdf. Accessed on 05 March 2024
35. National Institute of Population Studies: **Pakistan Demographic and Health Survey 2017–18. Available at: https://dhsprogram.com/pubs/pdf/FR290/FR290.pdf (2019). Accessed 1 April 2021.**

For peer review only - http://bmjopen.bmj.com/site/about/guidelines.xhtml

36. National Institute of Population Studies: **Pakistan Special Demographic and Health Survey 2019. Available at: https://dhsprogram.com/publications/publication-SR267-Summary-Reports-Key-Findings.cfm. Accessed 1 April 2021**

37. National Population Commission (NPC) [Nigeria] and ICF. 2019. **Nigeria Demographic and Health Survey 2018**. Abuja, Nigeria, and Rockville, Maryland, USA: NPC and ICF

38. De Silva MJ, Breuer E, Lee L, Asher L, Chowdhary N, Lund C, Patel V: **Theory of Change: a theory-driven approach to enhance the Medical Research Council's framework for complex interventions**. *Trials* 2014, **15**:267.

39. World Health Organization: **Everybody's business--strengthening health systems to improve health outcomes: WHO's framework for action**. Geneva: World Health Organization; 2007. https://www.who.int/healthsystems/strategy/everybodys_business.pdf. Accessed 15 May 2020.

40. World Health Organization: **Framework for ensuring human rights in the provision of contraceptive information and services**. In. Edited by World Health Organization. Geneva: World Health Organization,; 2014.

41. Committee on Economic Social and Cultural Rights (22nd session): **General comment No 14: The right to the highest attainable standard of health–Article 12 of the International Covenant on Economic, Social and Cultural Rights (CESCR)**. *Geneva: United Nations Economic and Social Council* 2000.

42. Macaulay AC, Commanda LE, Freeman WL, Gibson N, McCabe ML, Robbins CM, Twohig PL: **Participatory research maximises community and lay involvement**. *Bmj* 1999, **319**(7212):774-778.

43. Craig P, Dieppe P, Macintyre S, Michie S, Nazareth I, Petticrew M: **Developing and evaluating complex interventions: new guidance. Medical Research Council, 2008**. In.; 2010.

44. Dehlendorf C, Fox E, Silverstein IA, Hoffman A, Pérez MPC, Holt K, Reed R, Hessler D: **Development of the Person-Centered Contraceptive Counseling scale (PCCC), a short form of the Interpersonal Quality of Family Planning care scale**. *Contraception* 2021.

45. Zwarenstein M, Treweek S, Gagnier JJ, Altman DG, Tunis S, Haynes B, Oxman AD, Moher D: **Improving the reporting of pragmatic trials: an extension of the CONSORT statement**. *Bmj* 2008, **337**:a2390.

46. Weijer C, Grimshaw JM, Eccles MP, McRae AD, White A, Brehaut JC, Taljaard M: **The Ottawa Statement on the Ethical Design and Conduct of Cluster Randomized Trials**. *PLoS Med* 2012, **9**(11):e1001346.

47. Salimi Y, Shahandeh K, Malekafzali H, Loori N, Kheiltash A, Jamshidi E, Frouzan AS, Majdzadeh R: **Is Community-based Participatory Research (CBPR) Useful? A Systematic Review on Papers in a Decade**. *International Journal of Preventive Medicine* 2012, **3**(6):386-393.

48. Jagosh J, Macaulay AC, Pluye P, Salsberg J, Bush PL, Henderson J, Sirett E, Wong G, Cargo M, Herbert CP: **Uncovering the benefits of participatory research: implications of a realist review for health research and practice**. *The Milbank Quarterly* 2012, **90**(2):311-346.

For peer review only - http://bmjopen.bmj.com/site/about/guidelines.xhtml

729 **Figures, tables, additional files**

730 **Figure 1**. Overall study model.

731 **Figure 2.** Flow diagram of the cluster randomized control trial in each study country.

732 **Figure 3.** Schedule of enrolment, interventions, and assessments for the cluster randomized
733 controlled trial phase in each study country (SPIRIT template).

734

735

For peer review only

For peer review only - http://bmjopen.bmj.com/site/about/guidelines.xhtml

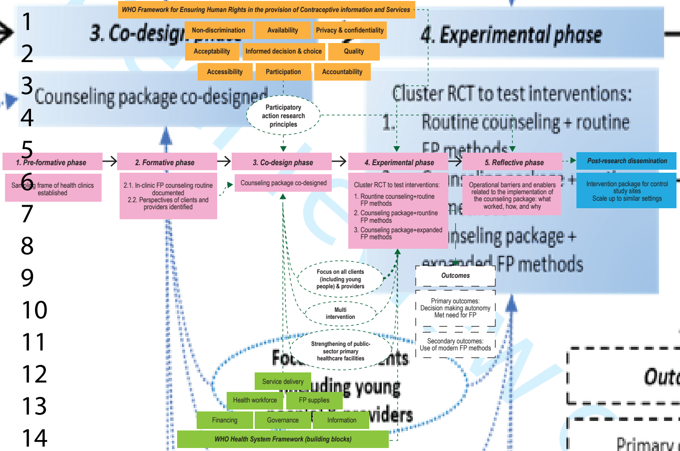

For peer review only - http://bmjopen.bmj.com/site/about/guidelines.xhtml

Figure 2. Flow diagram of the cluster randomized controlled trial (separately) in Pakistan and Nigeria.

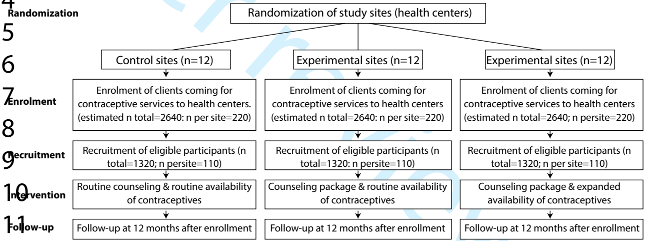

er review only - http://bmjopen.bmj.com/site/about/guideline

Figure 3. Schedule of enrolment, interventions, and assessments (SPIRIT template) for Pakistan and Nigeria (each country will use the same sampling framework)

	Enrolment	Allocation	Post-allocation			
**TIMEPOINT**	***t_{-1}*** *Initial study family planning visit*	***t_0*** *Initial study family planning visit*	***t_1*** *After initial study visit*	***t_2*** *After each follow-up visit*	***t_3*** *…*	***t_4*** *12 months*
**ENROLMENT:**						
Eligibility screen (estimate)	7920					
Informed consent	3960					
Allocation		3960	X	X	X	X
**INTERVENTIONS & CONTROL:**						
Counseling package & routine availability of contraceptives		1320	◆—	—	—	—◆
Counseling package & expanded availability of contraceptives		1320	◆—	—	—	—◆
Control: routine counseling & routine availability of contraceptives		1320	◆—	—	—	—◆
**ASSESSMENTS:**						
**Baseline variables:** Met need for family planning; Modern contraceptive use		X				
**Primary outcomes:** Decision-making autonomy; Met need for family planning			X	X	X	X
**Secondary outcomes:** Modern contraceptive use			X	X	X	X

For peer review only - http://bmjopen.bmj.com/site/about/guidelines.xhtml

STANDARD PROTOCOL ITEMS: RECOMMENDATIONS FOR INTERVENTIONAL TRIALS

SPIRIT 2013 Checklist: Recommended items to address in a clinical trial protocol and related documents*

Section/item	Item No	Description	Addressed on page number
**Administrative information**			
Title	1	Descriptive title identifying the study design, population, interventions, and, if applicable, trial acronym	_1____________
Trial registration	2a	Trial identifier and registry name. If not yet registered, name of intended registry	__3___________
	2b	All items from the World Health Organization Trial Registration Data Set	__3___________
Protocol version	3	Date and version identifier	__3__________
Funding	4	Sources and types of financial, material, and other support	___16________
Roles and responsibilities	5a	Names, affiliations, and roles of protocol contributors	___1, 16______
	5b	Name and contact information for the trial sponsor	___16________
	5c	Role of study sponsor and funders, if any, in study design; collection, management, analysis, and interpretation of data; writing of the report; and the decision to submit the report for publication, including whether they will have ultimate authority over any of these activities	___16__________
	5d	Composition, roles, and responsibilities of the coordinating centre, steering committee, endpoint adjudication committee, data management team, and other individuals or groups overseeing the trial, if applicable (see Item 21a for data monitoring committee)	____16_________

For peer review only

For peer review only - http://bmjopen.bmj.com/site/about/guidelines.xhtml

Introduction

Background and rationale	6a	Description of research question and justification for undertaking the trial, including summary of relevant studies (published and unpublished) examining benefits and harms for each intervention	________4______
	6b	Explanation for choice of comparators	_____7________
Objectives	7	Specific objectives or hypotheses	_____5________
Trial design	8	Description of trial design including type of trial (eg, parallel group, crossover, factorial, single group), allocation ratio, and framework (eg, superiority, equivalence, noninferiority, exploratory)	_____7________

Methods: Participants, interventions, and outcomes

Study setting	9	Description of study settings (eg, community clinic, academic hospital) and list of countries where data will be collected. Reference to where list of study sites can be obtained	________7______
Eligibility criteria	10	Inclusion and exclusion criteria for participants. If applicable, eligibility criteria for study centres and individuals who will perform the interventions (eg, surgeons, psychotherapists)	___9,11______
Interventions	11a	Interventions for each group with sufficient detail to allow replication, including how and when they will be administered	____8__________
	11b	Criteria for discontinuing or modifying allocated interventions for a given trial participant (eg, drug dose change in response to harms, participant request, or improving/worsening disease)	_____11________
	11c	Strategies to improve adherence to intervention protocols, and any procedures for monitoring adherence (eg, drug tablet return, laboratory tests)	_______8_______
	11d	Relevant concomitant care and interventions that are permitted or prohibited during the trial	______________
Outcomes	12	Primary, secondary, and other outcomes, including the specific measurement variable (eg, systolic blood pressure), analysis metric (eg, change from baseline, final value, time to event), method of aggregation (eg, median, proportion), and time point for each outcome. Explanation of the clinical relevance of chosen efficacy and harm outcomes is strongly recommended	____5__________
Participant timeline	13	Time schedule of enrolment, interventions (including any run-ins and washouts), assessments, and visits for participants. A schematic diagram is highly recommended (see Figure)	___Fig 3_____

For peer review only - http://bmjopen.bmj.com/site/about/guidelines.xhtml

Sample size	14	Estimated number of participants needed to achieve study objectives and how it was determined, including clinical and statistical assumptions supporting any sample size calculations	______Fig 3, 12___
Recruitment	15	Strategies for achieving adequate participant enrolment to reach target sample size	______________

Methods: Assignment of interventions (for controlled trials)

Allocation:

Sequence generation	16a	Method of generating the allocation sequence (eg, computer-generated random numbers), and list of any factors for stratification. To reduce predictability of a random sequence, details of any planned restriction (eg, blocking) should be provided in a separate document that is unavailable to those who enrol participants or assign interventions	______________
Allocation concealment mechanism	16b	Mechanism of implementing the allocation sequence (eg, central telephone; sequentially numbered, opaque, sealed envelopes), describing any steps to conceal the sequence until interventions are assigned	______________
Implementation	16c	Who will generate the allocation sequence, who will enrol participants, and who will assign participants to interventions	_____12__________
Blinding (masking)	17a	Who will be blinded after assignment to interventions (eg, trial participants, care providers, outcome assessors, data analysts), and how	__10____________
	17b	If blinded, circumstances under which unblinding is permissible, and procedure for revealing a participant's allocated intervention during the trial	______________

Methods: Data collection, management, and analysis

Data collection methods	18a	Plans for assessment and collection of outcome, baseline, and other trial data, including any related processes to promote data quality (eg, duplicate measurements, training of assessors) and a description of study instruments (eg, questionnaires, laboratory tests) along with their reliability and validity, if known. Reference to where data collection forms can be found, if not in the protocol	_____12__________
	18b	Plans to promote participant retention and complete follow-up, including list of any outcome data to be collected for participants who discontinue or deviate from intervention protocols	____12___________

Data management	19	Plans for data entry, coding, security, and storage, including any related processes to promote data quality (eg, double data entry; range checks for data values). Reference to where details of data management procedures can be found, if not in the protocol	_____13__________
Statistical methods	20a	Statistical methods for analysing primary and secondary outcomes. Reference to where other details of the statistical analysis plan can be found, if not in the protocol	____13___________
	20b	Methods for any additional analyses (eg, subgroup and adjusted analyses)	__13____________
	20c	Definition of analysis population relating to protocol non-adherence (eg, as randomised analysis), and any statistical methods to handle missing data (eg, multiple imputation)	_______________
**Methods: Monitoring**			
Data monitoring	21a	Composition of data monitoring committee (DMC); summary of its role and reporting structure; statement of whether it is independent from the sponsor and competing interests; and reference to where further details about its charter can be found, if not in the protocol. Alternatively, an explanation of why a DMC is not needed	_16_____________
	21b	Description of any interim analyses and stopping guidelines, including who will have access to these interim results and make the final decision to terminate the trial	_______________
Harms	22	Plans for collecting, assessing, reporting, and managing solicited and spontaneously reported adverse events and other unintended effects of trial interventions or trial conduct	_______________
Auditing	23	Frequency and procedures for auditing trial conduct, if any, and whether the process will be independent from investigators and the sponsor	_______________
**Ethics and dissemination**			
Research ethics approval	24	Plans for seeking research ethics committee/institutional review board (REC/IRB) approval	__13____________
Protocol amendments	25	Plans for communicating important protocol modifications (eg, changes to eligibility criteria, outcomes, analyses) to relevant parties (eg, investigators, REC/IRBs, trial participants, trial registries, journals, regulators)	____13___________

For peer review only - http://bmjopen.bmj.com/site/about/guidelines.xhtml

Consent or assent	26a	Who will obtain informed consent or assent from potential trial participants or authorised surrogates, and how (see Item 32)	___9___________
	26b	Additional consent provisions for collection and use of participant data and biological specimens in ancillary studies, if applicable	______________
Confidentiality	27	How personal information about potential and enrolled participants will be collected, shared, and maintained in order to protect confidentiality before, during, and after the trial	_____13________
Declaration of interests	28	Financial and other competing interests for principal investigators for the overall trial and each study site	____16__________
Access to data	29	Statement of who will have access to the final trial dataset, and disclosure of contractual agreements that limit such access for investigators	_____13________
Ancillary and post-trial care	30	Provisions, if any, for ancillary and post-trial care, and for compensation to those who suffer harm from trial participation	______________
Dissemination policy	31a	Plans for investigators and sponsor to communicate trial results to participants, healthcare professionals, the public, and other relevant groups (eg, via publication, reporting in results databases, or other data sharing arrangements), including any publication restrictions	___14___________
	31b	Authorship eligibility guidelines and any intended use of professional writers	______________
	31c	Plans, if any, for granting public access to the full protocol, participant-level dataset, and statistical code	___14___________
**Appendices**			
Informed consent materials	32	Model consent form and other related documentation given to participants and authorised surrogates	______________
Biological specimens	33	Plans for collection, laboratory evaluation, and storage of biological specimens for genetic or molecular analysis in the current trial and for future use in ancillary studies, if applicable	______________

*It is strongly recommended that this checklist be read in conjunction with the SPIRIT 2013 Explanation & Elaboration for important clarification on the items. Amendments to the protocol should be tracked and dated. The SPIRIT checklist is copyrighted by the SPIRIT Group under the Creative Commons "Attribution-NonCommercial-NoDerivs 3.0 Unported" license.

For peer review only - http://bmjopen.bmj.com/site/about/guidelines.xhtml

BMJ Open

Strengthening contraceptive counselling services to empower clients and meet their needs: protocol for a 2-stage, multi-phase complex intervention in Pakistan and Nigeria

Journal:	*BMJ Open*
Manuscript ID	bmjopen-2023-081967.R2
Article Type:	Protocol
Date Submitted by the Author:	07-May-2024
Complete List of Authors:	Tran, Nguyen Toan; University of Technology Sydney,<br>Ali, Moazzam; World Health Organization, Reproductive Health and Research<br>Azmat, Syed Khurram ; Jinnah Sindh Medical University, AAPNA - Institute of Public Health; Marie Stopes Society Pakistan, Technical Services<br>Seuc, Armando; World Health Organization, Department of Reproductive Health and Research<br>Olaolorun, Funmilola; University of Ibadan College of Medicine, Community Medicine<br>Awan, Muhammad Ali ; AAPNA Institute of Public Health (AIPH), Jinnah Sindh, Medical University, Rafiqui H.J, Iqbal Shaheed Rd, Karachi, Sindh 75510, Pakistan<br>Morhason-Bello, Imran; University of Ibadan College of Medicine, Department of Obstetrics and Gynaecology, Faculty of Clinical Sciences, College of Medicine/University College Hospital, University of Ibadan<br>Thom, Ellen Mpangananji ; World Health Organization Country Office in Islammabad Pakistan<br>Martin , Josepth ; World Health Organization Country Office in Abuja, Nigeria<br>Abubakar, Hanifah Datti ; Mohammed Abdullahi Wase Teaching Hospital, Nasarawa<br>Uzma, Qudsia ; World Health Organization Country Office in Islamabad Pakistan<br>Kiarie, James; World Health Organization, Department of Reproductive Health and Research
**Primary Subject Heading**:	Reproductive medicine
Secondary Subject Heading:	Health services research
Keywords:	Clinical Trial, Decision Making, Health Services, Public health < INFECTIOUS DISEASES

Strengthening contraceptive counselling services to empower clients and meet their needs: protocol for a 2-stage, multi-phase complex intervention in Pakistan and Nigeria

Tran Nguyen Toan[1,2,3] nguyentoan.tran@uts.edu.au
Moazzam Ali[1*] alimoa@who.int
Syed Khurram Azmat[4] khurramazmat@gmail.com
Armando Seuc[1] armandoseuc@infomed.sld.cu
Funmilola M. Olaolorun PI[5] fmolaolorun@gmail.com
Muhammad Ali Awan[4] alighous@gmail.com
Imran Morhason-Bello[6] imranmorhasonbello@gmail.com
Ellen Mpangananji Thom[7] thome@who.int
Josepth Martin[8] mjoseph@who.int
Hanifah Datti Abubakar[9] hanniedee83@gmail.com
Qudsia Uzma[7] uzmaq@who.int
James Kiarie[1] kiariej@who.int

*Corresponding author: alimoa@who.int

[1] Department of Reproductive Health Research, World Health Organization, Avenue Appia 20, 1211 Genève 27, Switzerland.

[2] Faculty of Medicine, University of Geneva, Rue Michel-Servet 1, 1206 Genève, Switzerland

[3]Australian Centre for Public and Population Health Research, Faculty of Health, University of Technology, PO Box 123, Sydney, NSW 2007, Australia

[4] APPNA Institute of Public Health (AIPH), Jinnah Sindh, Medical University, Rafiqui H.J, Iqbal Shaheed Rd, Karachi, Sindh 75510, Pakistan

[5] Department of Community Medicine, College of Medicine, University of Ibadan, Nigeria

[6]Department of Obstetrics and Gynaecology, College of Medicine, University of Ibadan, Nigeria

For peer review only - http://bmjopen.bmj.com/site/about/guidelines.xhtml

[7] World Health Organization Country Office in Islamabad Pakistan

[8] World Health Organization Country Office in Abuja, Nigeria

[9] Mohammed Abdullahi Wase Teaching Hospital, Nasarawa, Kano, Nigeria

Abstract

Background: High-quality contraceptive counseling can accelerate global efforts to reduce the unmet need for and suboptimal use of modern contraceptives. This study aims to identify a package of interventions designed to strengthen in Pakistan and Nigeria and determine their effectiveness in increasing client-level decision-making, autonomy and meeting of contraceptive needs.

Methods: A multi-site, two-stage and five-phase intervention design will start with a pre-formative, formative, design, experimental and reflective phase. The Pre-formative phase will map potential study sites and establish the sampling frame. The two-part formative phase will first use participatory approaches to identify clients' perspectives, including young couples and providers, to ensure research contextualization and address each interest group's needs and priorities followed by clinical observations of client-provider encounters to document routine care. The design workshop in the third phase will result in the development of a package of contraceptive counseling interventions. In the fourth experimental phase, a multi-intervention, three-arm, single-blinded, parallel cluster randomized-controlled trial will compare routine care (arm 1) with the contraceptive counseling package (arm 2) and the same package combined with wider method availability (arm 3). The study aims to enrol a total of 7920 participants. The reflective phase aims to identify implementation barriers and enablers. The outcomes are clients' level of decision-making autonomy and use of modern contraceptives.

Ethics and dissemination: Ethical approval for this study was obtained from the WHO Scientific and Ethics Review Committee (Protocol ID Pakistan: ERC 006232 and Nigeria ERC: 006523). Each study site is required and has obtained the necessary ethical and regulatory approvals that are required in each specific country. Findings will be presented at local, national and international conferences and disseminated by peer-review publications.

Trial registration: Registered in ClinicalTrials.gov (NCT06081842, 31 October 2023).

Keywords

Health services research, counseling, contraception, family planning, empowerment, autonomy, qualitative research, cluster randomized controlled trial.

For peer review only - http://bmjopen.bmj.com/site/about/guidelines.xhtml

Strengths and limitations of this study

- The main strength of this study is the mixed methodology approach. The mixed-method study offer valuable insights and can address research questions that quantitative or qualitative methods alone cannot adequately answer; however, the researchers must carefully consider the trade-offs and challenges associated with this approach. Quantitative data can provide statistical proof of results, whereas qualitative data can delve into the underlying mechanisms, participants' experiences, and contextual influences.
- A mixed-method study offers flexibility in adapting to unexpected findings or emerging questions. Researchers can use qualitative data to explore unanticipated outcomes or explore areas identified as important during the quantitative phase.
- Including stakeholders at the start of research will enhance understanding of service provision dynamics and support efforts towards identifying strategies to enhance positive male involvement and engagement in women's sexual reproductive health and rights.
- The study will be implemented in a limited geography of the target countries; therefore, caution should needs to be taken before generalising the findings.

Background

Overlooking women's and couples' need for quality contraceptive counseling constitutes a missed opportunity in family planning programs and services to ensure that every woman, man, and adolescent can enjoy the health, social, and economic benefits of modern contraception. Access to family planning is a human right enshrined in the United Nations' Sustainable Development Goal 3 on health and wellbeing and, specifically, its target 3.7: *ensure universal access to sexual and reproductive health-care services, including for family planning* [1]. Around 164 million women of reproductive age, have an unmet need for modern contraceptive methods despite increased contraceptive use over the past decades [2]. Satisfying the unmet need for contraception in low- and middle-income countries and offering all pregnant women and their newborns the standard care recommended by the World Health Organization (WHO) would result in dramatic reductions of unintended pregnancies by 68%, unsafe abortions by 72%, maternal deaths by 62%, and neonatal deaths by 69% [3]. Adequate contraceptive coverage among users can be improved by addressing suboptimal use and high discontinuation rates [4].

Effective contraceptive counseling can help individuals choose a method that meets their needs and preferences, manage any side effects, and continue using their preferred method or switch to an alternative one [5]. Contraceptive counseling could improve the effective use of modern contraception and reduce unmet need according to a systematic review that synthesized the evidence on the comparative effectiveness of different contraceptive counseling strategies [6]. The quality of contraceptive counseling in low-resource settings is often low. In Senegal, for example, only 18% of providers counseled their clients on the three-item Method Information Index (method use, side-effects, and timing of follow-up visits) [7]. Interventions targeting women requesting or initiating a chosen method, including structured counseling on side-effects, tend to show positive effects on contraceptive continuation [8-11]. Counseling interventions targeting

For peer review only - http://bmjopen.bmj.com/site/about/guidelines.xhtml

family planning service users [12-16], abortion service users [17-19], postpartum women [20-25], and those reached by community-based interventions [26-28] have inconsistent effects on contraceptive behavior and satisfaction outcomes. In contrast, increased contraceptive use often follows interventions introducing systematic contraceptive counseling among women attending non-family planning outpatient services [29-31]. However, these interventions often include expanded contraceptive method provision, and the observed changes cannot be attributed to counseling alone. High-quality evidence is generally limited, and no clear consensus exists on how best to deliver contraceptive counseling to meet clients' contraceptive needs and enhance their satisfaction.

Building on this systematic review, the World Health Organization (WHO) organized a geographically diverse think tank in May 2019 in Geneva, Switzerland, to identify research and guidance gaps and other opportunities to improve the quality of contraceptive counseling [32]. Equally important was the crafting of a definition on contraceptive counseling, which was adopted by think tank members [33]: *Contraceptive counseling is defined as the exchange of information on contraceptive methods based on an assessment of the client's needs, preferences and lifestyle to support decision-making as per the client's intentions. This includes the selection, discontinuation or switching of a contraceptive method. The key principles are based on coercion-free and informed choice; neutral, understandable and evidence-based information; collaborative and confidential decision-making process; ensuring respectful care, dignity and choice.* The definition took into consideration the core dimensions articulated in the literature and mirrors the principles that were deliberated by the think tank: a two-way discussion and decision-making process between the client and provider, sharing objective and user-friendly information, and fundamentally grounded in the rights to autonomy, dignity, privacy, respect, and participation, among others. In addition, person-centeredness is a critical component of quality in family planning [5]. The 4-item person-centered contraceptive counselling scale (PCCC) is a valid and reliable measure of person-centered contraceptive counseling that reflects patients' perspectives on contraceptive counseling [34]. For example, integrating Dehlendorf's 4-item validated tool: PCCC: person-centered contraceptive counselling scale: Think about your visit. How do you think the member of the health care team did? Please rate them on each of the following by circling a number (the scale will use – Poor, Fair, Good, Very good and Excellent). Hence, to measure decision-making autonomy, the following considerations need to factor:

- Respecting me as a person
- Letting me say what mattered to me about my birth control method
- Taking my preferences about my birth control seriously
- Giving me enough information to make the best decision about my birth control method

Furthermore, the think tank members agreed on a prioritized set of guidance, knowledge, and implementation research gaps [32]. Such gaps include, for example, understanding or identifying clients' experiences with and expectations of counseling in a variety of global settings; the types of settings different populations, particularly adolescents, unmarried women, migrants, gender non-conforming people, and religious/ethnic minorities, receive most of their contraceptive counseling, and the quality of counseling across these settings; the fluidity/ambivalence of

For peer review only - http://bmjopen.bmj.com/site/about/guidelines.xhtml

pregnancy intentions, risk perception (pregnancy and sexually transmitted infections), and women's understanding of their bodies; the independent effect of counseling from method provision; the core elements of high-quality of contraceptive counseling and how these differ by context and what provider characteristics affect counseling quality; the minimum skills and competencies for contraceptive counselors; the contents of a comprehensive package of best practices for FP counseling for different populations.

We have reviewed and used the currently available resources noted above such as Person-centered care and effective contraceptive counseling approaches, which focuses on persons' values and preferences, and are considered to improve the quality of care in family planning. These approaches are likely to promote greater patient autonomy, trust, and satisfaction with services. Majority of the research has been based on small, qualitative studies, this study, using mixed methods approach, will benefit from existing resources and have also highlight the rights based approach in the counselling package to understand and document its implementation and effectiveness in the context of two countries.

Study overall objective and hypothesis

Owing to these gaps combined with the socio-cultural challenges related to accessing contraception, we propose a multi-site, two-stage and 5-phase study with two main objectives. The objective is to identify and test the effectiveness of a package of contraceptive counseling interventions on improving clients' informed decision-making autonomy as well as meeting their need for family planning. The outcomes are changes in clients' decision-making autonomy and clients' met need for family planning. Secondary outcome will be change in modern contraceptive prevalence.

The research rests on the working hypotheses that the package will strengthen existing contraceptive counseling services; account for the views of key actors, including clients and providers; be in line with national health policies and WHO global standards; and take into consideration the scarcity of public resources.

Study countries: Pakistan and Nigeria

Pakistan

The study will be implemented in Pakistan in partnership with the Ministry of National Health Services, Regulations and Coordination.. The overall demographic, family planning, and reproductive health indicators based on the 2017-18 Demographic and Health Survey [35] note that rural settings fared worse than urban settings. The total fertility rate was 3.6 children per woman, modern contraceptive prevalance rate was 25% , overall unmet need for family planning was 17% and demand for family planning satisfied by a modern method 49%. In terms of family planning knowledge, more than 98% of urban and rural women had heard about any type of modern methods—before marriage, only 15% of women did. Regarding men's attitudes toward

For peer review only - http://bmjopen.bmj.com/site/about/guidelines.xhtml

contraceptive use, 27% believed that family planning use is a woman's business, while 16% thought it may encourage women to be promiscuous [36].

Nigeria

The study will also be implemented in Nigeria by College of Medicine, University of Ibadan. The study will be conducted in two study sites: Lagos (southern part of Nigeria) and Kano (northern part of Nigeria) states. These states were selected for the following reasons: (1) Data from the Performance Monitoring for Action (PMA) program are available for both states from annual surveys over the past three years, including information on the method information index plus and other family planning indicators (pmadata.org); (2) The team has a good network in both locations and will be able to readily collaborate with the State Ministries of Health; (3) Safety and security are relatively good with no serious threats in recent times.

The latest demographic, family planning, and reproductive health indicators based on the 2018 Demographic and Health Survey in Nigeria in 2019 showed that in general, rural settings fared worse than urban settings [37]. The total fertility rate was 5.3 children per woman; overall unmet need for family planning was 18.9%; demand for family planning satisfied by a modern method 33.9% and modern contraceptive prevalance rate was 12%. In terms of family planning knowledge, 93.9% of currently married women and 98.3% of sexually active unmarried women had heard about any type of modern contraceptive method.

Methods

Framework and perspectives

Theory of change

To ensure that the contraceptive counseling intervention package that will emerge from this trial strengthens existing family planning services and is effective, safe, and culturally appropriate, this health services research is informed by the theory of change framework as well as perspectives on human rights and the health system. Following the research findings, the long-term vision is to support the health system in developing a sustainable and scalable contraceptive counselling intervention package for Nigeria and Pakistan. Although the exact package has not yet been developed, based on the requirement set by the respective ministry of health/population in Pakistan and Nigeria, the researchers are willing to contribute to the development of provider training, post-training mentorship and the provision of IEC materials to enable the implementation of the intervention package.

The staged and phased approach adopted in this study, with the different specific objectives, levels of activities, and intermediate and final results (see Figure 1), is underpinned by the theory of change [38].

For peer review only - http://bmjopen.bmj.com/site/about/guidelines.xhtml

FIGURE 1 HERE

Health system perspective

First and related to the research model outlined in Figure 1, the study will rely on the WHO Health System Framework with its six system building blocks and link research and programmatic considerations for contraceptive counseling [39]. The six system building blocks are leadership/governance, health care financing, health workforce, medical products and technologies, information and research, and service delivery [39].

Human rights perspective

The research will use the WHO Framework for Ensuring Human Rights in the Provision of Contraceptive Information and Services [40]. The human rights framework comprises nine standards that interweave with the dimensions of quality of care: non-discrimination, availability, accessibility, acceptability, quality, informed-decision making, protection of privacy and confidentiality, participation, and accountability. The research team would ensure a person-oriented approach where no women or teenage girls are discriminated against (for example, in the provision of family planning information and services) because of their age, marital status, HIV status, minority status, or other medical or social factors [33].

With regard to the participation standard, the research will ensure active and informed participation of individuals in decision-making that affects them, including on matters related to their health [41]. Due to the complex social, cultural, and religious challenges and opportunities surrounding contraceptive use, our research design will assume that the following participatory action research (or participatory research) principles will be essential to meet the study objectives as well as gain insights into the implementation process. [42].

Study phases and timelines

The research entails a complex multi-site, two-stage and 5-phase (Figure 1): STAGE 1 is formative and will comprise of a pre-formative (phase 1) and a formative (phase 2). While the STAGE 2 is experimental and will include intervention design (phase 3), experimental (phase 4), and reflective (phase 5). The proposed methods of each phase are not intrinsically complex—the overall intervention is complex as it features a *number and difficulty of behaviors required by those delivering or receiving the intervention* and involves a *number of groups or organizational levels targeted by the intervention*, among others [43]. The design follows the recommendations from the Medical Research Council (2008/2010) to develop a complex intervention, determine its feasibility, and facilitate its implementation and evaluation [43]. Therefore, the phases are interconnected and have specific objectives, methodologies, and results.

STAGE 1: The specific objective of the pre-formative phase is to establish a sampling frame of potential study sites by mapping primary health centers according to pre-established criteria (both public and private facilities). Health centers are eligible if they (1) provide family planning services; (2) offer at least between three and four modern contraceptive methods,

For peer review only - http://bmjopen.bmj.com/site/about/guidelines.xhtml

including a barrier method such as condom, and a short-term method such as pills or injectables, a long-term method such as implants, intra-uterine device, and referrals for permanent methods to clients; (3) have no stock-outs of all contraceptive methods during the preceding three months as per the national policy; (4) have on average at least 25 family planning encounters per week or 100 family planning encounters per months at a selected high-performing facility (each of public and private sector) and have on average at least 12 FP encounters per week or 48 FP encounters per months at a selected low-performing facility (each of public and private sector); (5) are not recipients of technical support for family planning (capacity building, supplies) from other organizations at the time of recruitment and commit not to receive any such support during the whole study; and (6) are willing to participate.. Based on this sampling frame, four health centers (public and private sector) will be allocated to the formative phase while the others will be randomized to each arm of the experimental phase. The specific objectives of the ***formative phase*** are to (1) document the in-clinic contraceptive counseling routine using observation checklists and (2) seek through qualitative research the perspectives of clients, including young people, providers, and other stakeholders to ensure adequate contextualization of the research and address needs and priorities of each interest group regarding contraceptive counseling barriers and enablers.

STAGE 2: The specific objective of the ***intervention design phase*** is to co-design, based on the results of the formative phase and through a participatory and consensus-building approach engaging clients, providers, and other main actors, a package of contraceptive counseling to be implemented during the experimental phase. To be integrated into the final package, interventions will need to consider current human resources and clinical capacity, costs, acceptability, feasibility, scalability, and other field considerations.

The specific objective of the ***experimental phase*** is to determine, through a cluster randomized controlled trial (RCT) design, the effect of the contraceptive counseling package on clients' level of decision-making autonomy and demand satisfied for family planning. The three-armed cluster RCT comprise (1) a control arm with contraceptive counseling and care and a range of family planning methods that are available at field level per the national policy and guidelines; (2) an experimental arm implementing the contraceptive counseling package but still with a range of family planning methods that are available (upto 4 methods) per the national policy and guidelines at field level; and (3) another experimental arm with the contraceptive counseling package but with an expanded range of family planning methods (more than 4 methods) that are recommended by national policy and guidance.

The specific objective of the ***reflective phase*** is to identify operational barriers and enablers related to the package implementation using qualitative research. Once the package of interventions has been identified—and resources permitting, we might consider conducting a cost-effectiveness analysis to determine the counseling package cost-effectiveness.

The qualitative research and cluster RCT are detailed in the following sections.

Timelines

The study be tenataively initiated in November 2023 and it will be completed by November 2025. The analysis and report will be drafted by June 2026.

For peer review only - http://bmjopen.bmj.com/site/about/guidelines.xhtml

Qualitative research

General design

For the qualitative components of the study (in the formative and reflective phases), we will conduct in-depth interviews (IDIs) in Pakistan and Nigeria with members of the community (women , adolescent males and females, partners, people with influence), health staff (service providers, managers, Community Health Workers – CHWs), development partners, donor agencies, and national policymakers. The formative phase will involve four (4) conveniently sampled health centers (public and private) and their respective coverage areas in each country. These sites will be selected from the sampling frame determined in the pre-formative phase.

For the identification of contraceptive counselling barriers and enablers, separate IDIs of six to ten (6-10) participants will be conducted for the following categories of individuals (1) women who have used family planning services at the health center in the previous 12 months–: married and unmarried adolescent females (≤ 19 years), married and unmarried women 19-39 years, married and unmarried women ≥ 40 years [The study will include Women of Reproductive Age (WRA) – irrespective of their marital status and age group (including migrants refugee people, ethnic and religious minorities, women or men with disabilities, women or men with HIV etc. are inclusive] ; (2) married and unmarried men who are partners or husbands of women who have used family planning services at the health center in the previous 12 months, and married and unmarried adolescent males (<19 years); and (3) family planning service providers.

About the recruitment of men/male participants, the women participants will be first recruited based on their eligibility and recruitment criteria. If the female participant is selected, she will be asked whether she is using the FP method in consensus with her male partner/spouse or using it covertly. If she uses the FP method with the consensus of her male partner/spouse, we will recruit the accompanying male partners/spouses in the study. If the women participant is using the FP method covertly, then the women will be first asked to provide consent to be invited to study participation. If she does not consent, then men/male participants will be identified by the CHWs at the community level. In both cases, formal consent will be taken from the woman whether she is using the FP method covertly or in consensus with her male partner/spouse.

IDIs will also take place with clinic managers, supervisors, CHWs, policy makers, and other influential individuals/opinion leaders (selected purposively) who have observed, supervised, or monitored family planning services at/or the catchment of the selected public/private health facility. We anticipate that interviews will last between 45-60 mins and will take place in a safe, neutral space of the participant's choosing (e.g. town hall, office space, on porch outside participant's home, etc.). Both the recruiter and the participant will decide and agree on the location of the interview and this will be conveyed to the research team. Alternatively, the team will also identify pay-per-hour spaces as an option to present to the participant. The interview guides will cover the following themes related to contraceptive counselling: knowledge, attitudes, and practices; socio-cultural and health systems barriers and catalysts; and quality of care, including autonomy, informed decision-making, and other human rights considerations.

The qualitative research in the ***reflective phase*** will occur in three health centers in each of the three study arms (nine centers in total). The centers will be purposely sampled to include the

For peer review only - http://bmjopen.bmj.com/site/about/guidelines.xhtml

lowest, average, and highest performing ones in terms of measured outcomes to enable the broadest possible understanding. In line with the theory of change approach, the qualitative research will consist of an implementation evaluation to analyze barriers and enablers related to the delivery of contraceptive counseling and family planning services, assess intervention fidelity (in the experimental groups), and explore approaches for scaling up. Separate IDIs are planned for service providers and clients (≤ 19 years, 20-39 years, ≥ 40 years). IDIs are planned for clinic managers. The interview guides will cover the following themes related to contraceptive counseling: operational barriers and enablers; quality of care, including decision-making autonomy and other human rights perspectives; and scaling-up considerations.

Selection criteria and recruitment approach: A standard process of selection and consent-taking will be undertaken at each study site comprising potential study participants for each of the above categories of individuals (including vulnerable populations). A recruitment approach will follow, and the main actors who will facilitate the recruitment process of the potential target respondents for this research are a) selected service providers, b) Community Health Workers – working in the catchment area of the selected service providers, c) community-based opinion leaders, i.e., male, and female schoolteachers, social activist/ NGO workers. The country PI/Co-PI will train all of them on the study's main objectives, purpose, issues in ethics and the importance of consent.

Qualitative data management and analysis

At the country level, the study will engage a team of reseachers with relevant experience in qualitative methods and in working in local languages.

The interviews will occur in a location guaranteeing privacy. We anticipate that interviews will last between 45-60 mins and will take place in a safe, neutral space of the participant's choosing (e.g. town hall, office space, on porch outside participant's home, etc.). The interviews will be audiotaped after obtaining informed conent from participants. Research assistants with experience in transcription will transcribe and translate the audio records into English. Accuracy checks will be done by comparing transcripts with audio files.

Qualitative research management Softwares such as QSR Nvivo 12 or Atlas.ti , , will be used to perform a thematic analysis by establishing a basic codebook describing all the nodes and using it to code data. The codebook will be enriched with new emerging nodes during the coding process. Data will be coded by research assistants competent in Nvivo. To ensure the quality of data coding, inter-rater reliability will be tested by computing the Cohen's Kappa coefficient with a Kappa coefficient of 0.8 and above as an acceptable concordance threshold.

The formative phase results will be presented and discussed during the intervention design workshop with key members of the research team, selected service providers, and clients with a view to informing the bottom-up design of a contextually sensitive package of contraceptive counseling interventions. The views of each workshop participant will be considered when examining the potential effectiveness of possible interventions, acceptability to women and providers, feasibility, the potential for integration into the overall family planning programs, sustainability, and scalability.

For peer review only - http://bmjopen.bmj.com/site/about/guidelines.xhtml

Cluster randomized controlled trial

The research will be a multi-intervention, three-arm, single-blinded, parallel, cluster RCT done in selected primary health centers (clusters) in Pakistan and in Nigeria (see **Figure 2** for the cluster RCT flow diagram, and **Figure 3** for the schedule of enrolment, interventions, and assessments (SPIRIT template). Centers will be randomly allocated to the three study arms in matched ratios (1:1:1) on the basis of the number of monthly family planning encounters, the number of available contraceptive types, the ratio of health workers per population in the clinic coverage zone, the location in urban or rural settings, and selected district-level variables that may have an influence on the study outcomes, including the unmet need for family planning, the level of literacy among women, and household income terciles. Only data analysts will be masked/blinded to trial-arm allocation. Service providers skilled in family planning services will implement the clinical components of the contraceptive counseling package.

FIGURE 2 HERE

FIGURE 3 HERE

Outcome measures

Quantitative indicators will be captured in case-report forms (CRFs) developed and piloted at the country level before finalization. The proposed outcomes to be assessed after each visit, at mid-point (6 months after enrollment), and at the study end (12 months after enrollment) are oriented toward clients and consist of levels of clients' decision-making autonomy and demand satisfied for family planning.

To determine their level of decision-making autonomy, clients will be asked to answer questions about the different domains contained in the counseling definition (see Background). These domains comprise client-provider collaboration, client's needs and preferences, the quality of information, freedom from coercion, confidentiality, and dignity. The Person-Centered Contraceptive Counseling scale (PCCC), developed and tested in publicly-funded clinics in California will also be used [43]. The four PCCC questions fall under the domains of respect, eliciting—and giving serious consideration to—client's preferences, and information quality and it will be assessed during the intervention phase as per described by UNFPA to measure decision-making autonomy SDG 5.6.1 [44].

The study proposes nonetheless the another outcome, while acknowledging its limitations: modern contraceptive prevalence after the first visit and at 6 and 12 months after enrollment.

For peer review only - http://bmjopen.bmj.com/site/about/guidelines.xhtml

Eligibility criteria for clusters (health centers) and participants

The study will be conducted in selected primary health centers. The eligibility criteria of health centers were described above under the objective of the pre-formative phase. A woman will be eligible to participate in the study if

(1) she comes to the family planning clinic with the intention to (i) use contraception for the first time in her life (new user), or (ii) switch from a contraceptive method to another one (switching user), or (iii) resume a method after not using any in the prior three months (lapse user), or (iv) discontinue a modern method (discontinuing user);

(2) she is not coming for the resupply of a currently used method, such as pills or injectables;

(3) she has the intention to continue her follow-up at the health center during the 12-month study follow-up;

(4) she does not participate in another study; and

(5) she provides informed consent.

Exclusion Criteria: Not willing to participate in the complete duration of the trial.

Interventions

The health centers of the first experimental group will implement the intervention package, and those of the second experimental group will implement the intervention package in addition to an expanded range of family planning methods that are recommended by national guidance. The control group will implement usual care. Depending on the nature of the final package, some interventions may be implemented before participant recruitment, such as the capacity building of providers.

Randomization

In each country, the 36 sites will be matched on a 1:1:1 ratio according to the above-described criteria. Within each of the 12 trios, study sites will be randomly assigned to each of the three study arms. This randomization is performed 12 times (once for each trio). No restriction in the randomization process is required and masking cannot be done except for data analysts. All consecutive and eligible participants will be included in the clusters until the sample size of 7920 is achieved.

Data source and collection

For peer review only - http://bmjopen.bmj.com/site/about/guidelines.xhtml

Data on key outcome measures as well as quantitative process indicators will be collected on paper-based/electronic CRFs. The WHO team in Geneva will develop the CRFs with inputs from the country research teams. We will test advanced drafts with an appropriate sample of service providers and mock clients from the formative phase sites before finalizing the forms. Each health center of the cluster RCT will have a research assistant who will be trained to adhere to the study manual and standard operating procedures for data management which are common to both study countries and have been developed by WHO. The information reported on the CRFs will be checked for accuracy and completeness several times at different levels by field coordinators and data managers.

Data management

As for other multicenter studies conducted and sponsored by the UNDP/UNFPA/UNICEF/WHO/World Bank Special Programme of Research, Development and Research Training in Human Reproduction (HRP) at WHO in Geneva, data entry and data quality monitoring will be carried out using a web-based data management system. It will be developed by HRP's Biostatistics and Data Management team using OpenClinica (version 3.16), an electronic data capture software for clinical research that includes programmed edit checks for data quality, completeness, and consistency reduces delays in data queries and problem resolution and allows the dataset to be easily available. The OpenClinica system is a highly secure, open-source, and trusted web-based software solution that is fully compliant with Good Clinical Practice (GCP) and regulatory guidelines and HRP/WHO Standard Operating Procedures for clinical trial management.

The country research teams in Pakistan and Nigeria will enter the data locally and have it reviewed by the WHO team in Geneva. We will train the country teams in data entry and data management through OpenClinica system. Country research teams will be responsible for verifying the data, uploading it onto shared files, and entering the data into the OpenClinica system. Double data entry will be done to minimize errors. The system can produce automatic data queries (missing values, outliers, inconsistencies, and other errors). Data quality will be monitored by the WHO team. Questions regarding data inconsistencies or missing values will be submitted to sites via standardized forms and solved on a continuous basis. To ensure data integrity and participant confidentiality, data transmission will be encrypted. Only approved users will be given access to the data, which will be password protected.

Statistical analysis

We will prepare a comprehensive statistical analysis plan. Participants will form the unit of analysis and the clustered nature of the data will be accounted for. All analyses will be by intention to treat. For continuous variables, we will compute means, standard deviations, minimum and maximum values, and for categorical variables, we will compute frequencies and percentages. We will look at the distribution of variables and see if there are any outliers as part of data quality control and descriptive analysis. Descriptive statistics will be tabulated for individual clusters and aggregated across clusters. Within each of the three groups/arms of the study (two experimental and one control), outcome differences between final (12 months after enrolment) and intermediate (6 months after enrolment) outcome measurements will be obtained, and these changes will be compared between the control group and each of the two experimental

For peer review only - http://bmjopen.bmj.com/site/about/guidelines.xhtml

groups. For categorical outcomes, chi-square tests for two independent proportions will be used, and for continuous variables, t-student tests for two independent means will be used. We will use IBM SPSS Statistics 25, R 3.6.2, STATA 16.1, or SAS 9.4 statistical packages for the analysis. The findings will be presented using the CONSORT Statement checklist from 2010 and its extension for cluster studies [45].

Patient and public involvement

Patients or the public were not involved in the design, or conduct, or reporting, or dissemination plans of this research.

Ethics and regulatory aspects

We followed the guidelines in The Ottawa Statement on the Ethical Design and Conduct of Cluster Randomized Trials while designing this experiment [46]. The Research Project Review Panel (RP2) of the HRP peer-reviewed the protocol, and RP2 and the WHO Research Ethics Review Committee, Geneva, Switzerland (Pakistan: ERC 006232 and Nigeria ERC: 006523), approved it and so did the IRB of Research and Development Solutions (IRB No:00010843) in Pakistan. While the Ministry of Health, Kano State of Nigeria (NHREC 17/03/2018) and Lagos Research Ethics Committee in Nigeria (LREC/06/10/2180) provided ethical approvals for the Nigeria study .

This research is grounded in human rights principles, specifically participation in the form of participatory action research. The existing literature shows promise that our proposed approach may be successful. For example, a synthesis of the evidence on community-based participatory research in different settings indicates that collaboration among community partners, researchers, and organizations led to community-level action that enhanced health and wellbeing while minimizing health disparities [47]. In the process, it also strengthened the capacity of the community in terms of research and leadership skills. Another review found that participatory action research could ensure that research is appropriate culturally and logistically; enhance recruitment capacity; generate professional capacity and competence in stakeholder groups; result in productive conflicts followed by useful negotiation; increase the quality of outputs and outcomes over time; increase the sustainability of project goals beyond funded time frames and during gaps in external funding; and create system changes and new unanticipated projects and activities [48].

Dissemination

The findings from the analysis of this study will be disseminated in a variety of ways. At country level, the results will be presented to policy makers, researchers, managers through policy briefs and workshops in collaboration with the WHO office. The results will also be disseminated among the communities/participants through online platforms/text messages, which will be provided to the participants by the data collectors/health workers. In addition, the results will also be presented to the scientific and funding community in collaboration with WHO country and regional offices, by communication and manuscript publications in peer-reviewed international journals.

For peer review only - http://bmjopen.bmj.com/site/about/guidelines.xhtml

Declarations

Ethics approval and consent to participate

The research protocol was approved by the Research Project Review Panel (RP2) of the UNDP/UNFPA/UNICEF/WHO/World Bank Special Programme of Research, Development and Research Training in Human Reproduction (HRP) at WHO in Geneva, and the WHO Research Ethics Review Committee, Geneva, Switzerland. The study was approved by the ethics review committee in Pakistan and Nigeria. All adult participants will provide written consent. Non-adult participants will give their informed assent and their respective parents or guardians will provide their informed consent.

Consent for publication:

Not applicable.

Availability of data and materials

Not applicable.

Competing interests

The authors declare that they have no competing interests.

Patient and public involvement Patients and/or the public were not involved in the design, or conduct, or reporting, or dissemination plans of this research.

Funding

The project site in Pakistan has been supported by the UNDP-UNICEF-WHO-World Bank Special Programme of Research, Development and Research Training in Human Reproduction Programme (HRP grant No. TRIMS: A66026); a co-sponsored programme executed by the World Health Organisation (WHO). The project in Nigeria generously received funding from United States Agency for International Development (USAID grant No. GHA-G-00-09-00003). The funders will take no role in subjects' recruitment, data collection, analyses, or interpretation, and will take no part in the decision to publish.

Author's contributions

MA, TNT, and AS designed the initial study concept, the study protocol, and study instruments. SKA,MA, MAA and FMO provided revisions to multiple versions of the study protocol based on the feedback from WHO-ERC. SKA, MA, FMO, MAA, EMT, JM, QU, JK,HDA and IMB

For peer review only

For peer review only - http://bmjopen.bmj.com/site/about/guidelines.xhtml

contributed to the final study protocol and study instruments. TNT wrote the first draft of the manuscript with the contributions of MA, AS, and KJ. While, SKA and MA developed the final draft of the manuscript. All authors read and approved the final manuscript.

Acknowledgements

The authors are grateful to Dr. Onikepe Owolabi of the Guttmacher Institute and Dr. Nathalie Roos of the Karolinska Institute for their technical support and insights.

ORCID:

Moazzam Ali http://orcid.org/0000-0001-6949-8976

Syed Khurram Azmat https://orcid.org/0000-0003-0158-8734

References

1. Howden-Chapman P, Siri J, Chisholm E, Chapman R, Doll CN, Capon A: **SDG 3: Ensure healthy lives and promote wellbeing for all at all ages**. *A guide to SDG interactions: from science to implementation Paris, France: International Council for Science* 2017:81-126.
2. United Nations Department of Economic and Social Affairs, Population Division (2022). World Family Planning 2022: Meeting the changing needs for family planning: Contraceptive use by age and method.UN DESA/POP/2022/TR/NO. 4
3. Sully E, Biddlecom A, Darroch J, Ashford L, Lince-Deroche N, Riley T: **Adding It Up: Investing in Sexual and Reproductive Health, 2020**. *New York, NY: Guttmacher Institute*.
4. Polis CB, Bradley SE, Bankole A, Onda T, Croft T, Singh S: **Contraceptive failure rates in the developing world: an analysis of demographic and health survey data in 43 countries**. *New York: Guttmacher Institute* 2016.
5. Dehlendorf C, Krajewski C, Borrero S: **Contraceptive counseling: best practices to ensure quality communication and enable effective contraceptive use**. *Clinical obstetrics and gynecology* 2014, **57**(4):659.
6. Cavallaro FL, Benova L, Owolabi OO, Ali M: **A systematic review of the effectiveness of counselling strategies for modern contraceptive methods: what works and what doesn't?** *BMJ Sexual & Reproductive Health* 2019.
7. Assaf S, Wang W, Mallick L: **Provider Counseling and Knowledge Transfer in Health Facilities of Haiti, Malawi, and Senegal. DHS Analytical Studies No. 60**. *Rockville, Maryland* 2016.
8. De Cetina TEC, Canto P, Luna MO: **Effect of counseling to improve compliance in Mexican women receiving depot-medroxyprogesterone acetate**. *Contraception* 2001, **63**(3):143-146.
9. Modesto W, Bahamondes MV, Bahamondes L: **A randomized clinical trial of the effect of intensive versus non-intensive counselling on discontinuation rates due to bleeding disturbances of three long-acting reversible contraceptives**. *Hum Reprod* 2014, **29**(7):1393-1399.

For peer review only - http://bmjopen.bmj.com/site/about/guidelines.xhtml

10. Patel D, Koenig M, Patel A, Rani M: **The impact of improved service quality on IUD continuation: evidence from rural India**. *JOURNAL OF FAMILY WELFARE* 2003, **49**:1-9.
11. Amatya R, Akhter H, McMahan J, Williamson N, Gates D, Ahmed Y: **The effect of husband counseling on NORPLANT® contraceptive acceptability in Bangladesh**. *Contraception* 1994, **50**(3):263-273.
12. Jain AK, RamaRao S, Kim J, Costello M: **Evaluation of an intervention to improve quality of care in family planning programme in the Philippines**. *Journal of biosocial science* 2012, **44**(1):27.
13. Chin-Quee DS, Janowitz B, Otterness C: **Counseling tools alone do not improve method continuation: further evidence from the decision-making tool for family planning clients and providers in Nicaragua**. *Contraception* 2007, **76**(5):377-382.
14. Sanogo D, RamaRao S, Jones H, N'diaye P, M'bow B, Diop CB: **Improving quality of care and use of contraceptives in Senegal**. *African journal of reproductive health* 2003:57-73.
15. Sapkota S, Rajbhandary R, Lohani S: **The impact of balanced counseling on contraceptive method choice and determinants of long acting and reversible contraceptive continuation in Nepal**. *Maternal and child health journal* 2017, **21**(9):1713-1723.
16. Kim YM, Putjuk F, Basuki E, Kols A: **Increasing patient participation in reproductive health consultations: an evaluation of "Smart Patient" coaching in Indonesia**. *Patient education and counseling* 2003, **50**(2):113-122.
17. Zhu JL, Zhang W-H, Cheng Y, Xu J, Xu X, Gibson D, Støvring H, Claeys P, Temmerman M: **Impact of post-abortion family planning services on contraceptive use and abortion rate among young women in China: a cluster randomised trial**. *The European Journal of Contraception & Reproductive Health Care* 2009, **14**(1):46-54.
18. Ferreira ALCG, Souza AI, Pessoa RE, Braga C: **The effectiveness of contraceptive counseling for women in the postabortion period: an intervention study**. *Contraception* 2011, **84**(4):377-383.
19. Smith C, Ngo TD, Gold J, Edwards P, Vannak U, Sokhey L, Machiyama K, Slaymaker E, Warnock R, McCarthy O: **Effect of a mobile phone-based intervention on post-abortion contraception: a randomized controlled trial in Cambodia**. *Bulletin of the World Health Organization* 2015, **93**:842-850A.
20. Kaewkiattikun K: **Effects of immediate postpartum contraceptive counseling on long-acting reversible contraceptive use in adolescents**. *Adolescent health, medicine and therapeutics* 2017, **8**:115.
21. Bolam A, Manandhar DS, Shrestha P, Ellis M, Anthony MdL: **The effects of postnatal health education for mothers on infant care and family planning practices in Nepal: a randomised controlled trial**. *Bmj* 1998, **316**(7134):805-811.
22. Adanikin AI, Onwudiegwu U, Loto OM: **Influence of multiple antenatal counselling sessions on modern contraceptive uptake in Nigeria**. *Eur J Contracept Reprod Health Care* 2013, **18**(5):381-387.
23. Vural F, Vural B, Cakiroglu Y: **The effect of combined antenatal and postnatal counselling on postpartum modern contraceptive use: prospective case-control study in Kocaeli, Turkey**. *Journal of clinical and diagnostic research: JCDR* 2016, **10**(4):QC04.
24. Fatima P, Antora AH, Dewan F, Nash S, Sethi M: **Impact of contraceptive counselling training among counsellors participating in the FIGO postpartum intrauterine device initiative in Bangladesh**. *International Journal of Gynecology & Obstetrics* 2018, **143**:49-55.
25. Ndegwa SW, Gichuhi J, Qureshi Z, Lubano K: **The effect of two levels of counselling on acceptance, uptake and early outcomes of post-placental intra-uterine contraceptive device**. *East African medical journal* 2014, **91**(12):449-456.
26. Terefe A, Larson CP: **Modern contraception use in Ethiopia: does involving husbands make a difference?** *American Journal of Public Health* 1993, **83**(11):1567-1571.

For peer review only - http://bmjopen.bmj.com/site/about/guidelines.xhtml

27. El-Khoury M, Thornton R, Chatterji M, Kamhawi S, Sloane P, Halassa M: **Counseling women and couples on family planning: a randomized study in Jordan**. *Studies in family planning* 2016, **47**(3):222-238.

28. Lemani C, Tang JH, Kopp D, Phiri B, Kumvula C, Chikosi L, Mwale M, Rosenberg NE: **Contraceptive uptake after training community health workers in couples counseling: A cluster randomized trial**. *PloS one* 2017, **12**(4):e0175879.

29. Gillespie D, Bradley H, Woldegiorgis M, Kidanu A, Karklins S: **Integrating family planning into Ethiopian voluntary testing and counselling programmes**. *Bulletin of the World Health Organization* 2009, **87**:866-870.

30. Lee J, Papic M, Baldauf E, Updike G, Schwarz EB: **A checklist approach to caring for women seeking pregnancy testing: effects on contraceptive knowledge and use**. *Contraception* 2015, **91**(2):143-149.

31. Yassin AS, Cordwell D: **Does dedicated pre-abortion contraception counselling help to improve post-abortion contraception uptake?** *BMJ Sexual & Reproductive Health* 2005, **31**(2):115-116.

32. Ali M, Tran NT, Kabra R, Kiare J: **Strengthening contraceptive counselling: gaps in knowledge and implementation research**. In.: British Medical Journal Publishing Group; 2021.

33. Ali M, Tran NT: **Defining counselling in contraceptive information and services: outcomes from an expert think tank**. In.: British Medical Journal Publishing Group; 2021.

34. Dehlendorf C, Fox E, Silverstein IA, Hoffman A, Pérez MPC, Holt K, Reed R, Hessler D: Development of the Person-Centered Contraceptive Counseling scale (PCCC), a short form of the Interpersonal Quality of Family Planning care scale. Contraception 2021.

35. National Institute of Population Studies: **Pakistan Demographic and Health Survey 2017–18. Available at: https://dhsprogram.com/pubs/pdf/FR290/FR290.pdf (2019). Accessed 1 April 2021.**

36. National Institute of Population Studies: **Pakistan Special Demographic and Health Survey 2019. Available at: https://dhsprogram.com/publications/publication-SR267-Summary-Reports-Key-Findings.cfm. Accessed 1 April 2021**

37. National Population Commission (NPC) [Nigeria] and ICF. 2019. **Nigeria Demographic and Health Survey 2018**. Abuja, Nigeria, and Rockville, Maryland, USA: NPC and ICF

38. De Silva MJ, Breuer E, Lee L, Asher L, Chowdhary N, Lund C, Patel V: **Theory of Change: a theory-driven approach to enhance the Medical Research Council's framework for complex interventions**. *Trials* 2014, **15**:267.

39. World Health Organization: **Everybody's business--strengthening health systems to improve health outcomes: WHO's framework for action**. Geneva: World Health Organization; 2007. https://www.who.int/healthsystems/strategy/everybodys_business.pdf. Accessed 15 May 2020.

40. World Health Organization: **Framework for ensuring human rights in the provision of contraceptive information and services**. In. Edited by World Health Organization. Geneva: World Health Organization,; 2014.

41. Committee on Economic Social and Cultural Rights (22nd session): **General comment No 14: The right to the highest attainable standard of health–Article 12 of the International Covenant on Economic, Social and Cultural Rights (CESCR)**. *Geneva: United Nations Economic and Social Council* 2000.

42. Macaulay AC, Commanda LE, Freeman WL, Gibson N, McCabe ML, Robbins CM, Twohig PL: **Participatory research maximises community and lay involvement**. *Bmj* 1999, **319**(7212):774-778.

43. Craig P, Dieppe P, Macintyre S, Michie S, Nazareth I, Petticrew M: **Developing and evaluating complex interventions: new guidance. Medical Research Council, 2008**. In.; 2010.

For peer review only - http://bmjopen.bmj.com/site/about/guidelines.xhtml

44. UNFPA and Hera (2019). Research on what determines women's ability to decide on their SRHR and the relationship between this and other aspects of their lives. Volumes 1 and 2: Final report, October 2019. https://www.unfpa.org/sites/default/files/resource-pdf/20-033_SDG561-IssueBrief-v4.1.pdf. Accessed on 05 March 2024

45. Zwarenstein M, Treweek S, Gagnier JJ, Altman DG, Tunis S, Haynes B, Oxman AD, Moher D: **Improving the reporting of pragmatic trials: an extension of the CONSORT statement**. *Bmj* 2008, **337**:a2390.

46. Weijer C, Grimshaw JM, Eccles MP, McRae AD, White A, Brehaut JC, Taljaard M: **The Ottawa Statement on the Ethical Design and Conduct of Cluster Randomized Trials**. *PLoS Med* 2012, **9**(11):e1001346.

47. Salimi Y, Shahandeh K, Malekafzali H, Loori N, Kheiltash A, Jamshidi E, Frouzan AS, Majdzadeh R: **Is Community-based Participatory Research (CBPR) Useful? A Systematic Review on Papers in a Decade**. *International Journal of Preventive Medicine* 2012, **3**(6):386-393.

48. Jagosh J, Macaulay AC, Pluye P, Salsberg J, Bush PL, Henderson J, Sirett E, Wong G, Cargo M, Herbert CP: **Uncovering the benefits of participatory research: implications of a realist review for health research and practice**. *The Milbank Quarterly* 2012, **90**(2):311-346.

For peer review only

For peer review only - http://bmjopen.bmj.com/site/about/guidelines.xhtml

717 **Figures, tables, additional files**

718 **Figure 1**. Overall study model.

719 **Figure 2.** Flow diagram of the cluster randomized control trial in each study country.

720 **Figure 3.** Schedule of enrolment, interventions, and assessments for the cluster randomized
721 controlled trial phase in each study country (SPIRIT template).

722

723

For peer review only

For peer review only - http://bmjopen.bmj.com/site/about/guidelines.xhtml

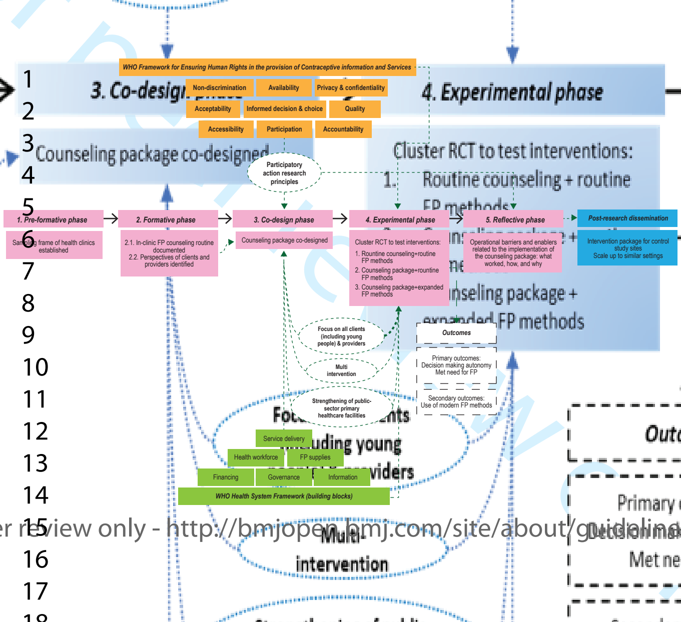

For peer review only - http://bmjopen.bmj.com/site/about/guidelines.xhtml

Figure 2. Flow diagram of the cluster randomized controlled trial (separately) in Pakistan and Nigeria.

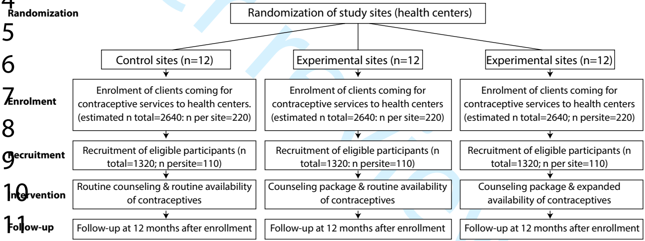

er review only - http://bmjopen.bmj.com/site/about/guideline

Figure 3. Schedule of enrolment, interventions, and assessments (SPIRIT template) for Pakistan and Nigeria (each country will use the same sampling framework)

	**Enrolment**	**Allocation**	**Post-allocation**			
**TIMEPOINT**	t_{-1} *Initial study family planning visit*	t_0 *Initial study family planning visit*	t_1 *After initial study visit*	t_2 *After each follow-up visit*	t_3 *…*	t_4 *12 months*
**ENROLMENT:**						
Eligibility screen (estimate)	7920					
Informed consent	3960					
Allocation		3960	X	X	X	X
**INTERVENTIONS & CONTROL:**						
Counseling package & routine availability of contraceptives		1320	◆	—	—	◆
Counseling package & expanded availability of contraceptives		1320	◆	—	—	◆
Control: routine counseling & routine availability of contraceptives		1320	◆	—	—	◆
**ASSESSMENTS:**						
**Baseline variables:** Met need for family planning; Modern contraceptive use		X				
**Primary outcomes:** Decision-making autonomy; Met need for family planning			X	X	X	X
**Secondary outcomes:** Modern contraceptive use			X	X	X	X

For peer review only - http://bmjopen.bmj.com/site/about/guidelines.xhtml

SPIRIT

STANDARD PROTOCOL ITEMS: RECOMMENDATIONS FOR INTERVENTIONAL TRIALS

SPIRIT 2013 Checklist: Recommended items to address in a clinical trial protocol and related documents*

Section/item	Item No	Description	Addressed on page number
**Administrative information**			
Title	1	Descriptive title identifying the study design, population, interventions, and, if applicable, trial acronym	_1____________
Trial registration	2a	Trial identifier and registry name. If not yet registered, name of intended registry	__2___________
	2b	All items from the World Health Organization Trial Registration Data Set	__2___________
Protocol version	3	Date and version identifier	__2__________
Funding	4	Sources and types of financial, material, and other support	___15________
Roles and responsibilities	5a	Names, affiliations, and roles of protocol contributors	___1, 15______
	5b	Name and contact information for the trial sponsor	___15________
	5c	Role of study sponsor and funders, if any, in study design; collection, management, analysis, and interpretation of data; writing of the report; and the decision to submit the report for publication, including whether they will have ultimate authority over any of these activities	___15__________
	5d	Composition, roles, and responsibilities of the coordinating centre, steering committee, endpoint adjudication committee, data management team, and other individuals or groups overseeing the trial, if applicable (see Item 21a for data monitoring committee)	____15_________

For peer review only - http://bmjopen.bmj.com/site/about/guidelines.xhtml

Introduction

Background and rationale	6a	Description of research question and justification for undertaking the trial, including summary of relevant studies (published and unpublished) examining benefits and harms for each intervention	_____4______
	6b	Explanation for choice of comparators	_____7________
Objectives	7	Specific objectives or hypotheses	_____4________
Trial design	8	Description of trial design including type of trial (eg, parallel group, crossover, factorial, single group), allocation ratio, and framework (eg, superiority, equivalence, noninferiority, exploratory)	_____7________

Methods: Participants, interventions, and outcomes

Study setting	9	Description of study settings (eg, community clinic, academic hospital) and list of countries where data will be collected. Reference to where list of study sites can be obtained	________7______
Eligibility criteria	10	Inclusion and exclusion criteria for participants. If applicable, eligibility criteria for study centres and individuals who will perform the interventions (eg, surgeons, psychotherapists)	___9,11______
Interventions	11a	Interventions for each group with sufficient detail to allow replication, including how and when they will be administered	____7,8_________
	11b	Criteria for discontinuing or modifying allocated interventions for a given trial participant (eg, drug dose change in response to harms, participant request, or improving/worsening disease)	_____11________
	11c	Strategies to improve adherence to intervention protocols, and any procedures for monitoring adherence (eg, drug tablet return, laboratory tests)	_______8_______
	11d	Relevant concomitant care and interventions that are permitted or prohibited during the trial	_____8________
Outcomes	12	Primary, secondary, and other outcomes, including the specific measurement variable (eg, systolic blood pressure), analysis metric (eg, change from baseline, final value, time to event), method of aggregation (eg, median, proportion), and time point for each outcome. Explanation of the clinical relevance of chosen efficacy and harm outcomes is strongly recommended	____4__________
Participant timeline	13	Time schedule of enrolment, interventions (including any run-ins and washouts), assessments, and visits for participants. A schematic diagram is highly recommended (see Figure)	___Fig 3_____

For peer review only - http://bmjopen.bmj.com/site/about/guidelines.xhtml

Sample size	14	Estimated number of participants needed to achieve study objectives and how it was determined, including clinical and statistical assumptions supporting any sample size calculations	___Fig 3, 11___
Recruitment	15	Strategies for achieving adequate participant enrolment to reach target sample size	_____11______
**Methods: Assignment of interventions (for controlled trials)**			
Allocation:			
Sequence generation	16a	Method of generating the allocation sequence (eg, computer-generated random numbers), and list of any factors for stratification. To reduce predictability of a random sequence, details of any planned restriction (eg, blocking) should be provided in a separate document that is unavailable to those who enrol participants or assign interventions	____11_________
Allocation concealment mechanism	16b	Mechanism of implementing the allocation sequence (eg, central telephone; sequentially numbered, opaque, sealed envelopes), describing any steps to conceal the sequence until interventions are assigned	____11_________
Implementation	16c	Who will generate the allocation sequence, who will enrol participants, and who will assign participants to interventions	____12_________
Blinding (masking)	17a	Who will be blinded after assignment to interventions (eg, trial participants, care providers, outcome assessors, data analysts), and how	__11___________
	17b	If blinded, circumstances under which unblinding is permissible, and procedure for revealing a participant's allocated intervention during the trial	____11_________
**Methods: Data collection, management, and analysis**			
Data collection methods	18a	Plans for assessment and collection of outcome, baseline, and other trial data, including any related processes to promote data quality (eg, duplicate measurements, training of assessors) and a description of study instruments (eg, questionnaires, laboratory tests) along with their reliability and validity, if known. Reference to where data collection forms can be found, if not in the protocol	____12_________
	18b	Plans to promote participant retention and complete follow-up, including list of any outcome data to be collected for participants who discontinue or deviate from intervention protocols	___12__________

Data management	19	Plans for data entry, coding, security, and storage, including any related processes to promote data quality (eg, double data entry; range checks for data values). Reference to where details of data management procedures can be found, if not in the protocol	____12_________
Statistical methods	20a	Statistical methods for analysing primary and secondary outcomes. Reference to where other details of the statistical analysis plan can be found, if not in the protocol	___12__________
	20b	Methods for any additional analyses (eg, subgroup and adjusted analyses)	__12________
	20c	Definition of analysis population relating to protocol non-adherence (eg, as randomised analysis), and any statistical methods to handle missing data (eg, multiple imputation)	_12____________
**Methods: Monitoring**			
Data monitoring	21a	Composition of data monitoring committee (DMC); summary of its role and reporting structure; statement of whether it is independent from the sponsor and competing interests; and reference to where further details about its charter can be found, if not in the protocol. Alternatively, an explanation of why a DMC is not needed	_13__________
	21b	Description of any interim analyses and stopping guidelines, including who will have access to these interim results and make the final decision to terminate the trial	___12__________
Harms	22	Plans for collecting, assessing, reporting, and managing solicited and spontaneously reported adverse events and other unintended effects of trial interventions or trial conduct	___12__________
Auditing	23	Frequency and procedures for auditing trial conduct, if any, and whether the process will be independent from investigators and the sponsor	___12__________
**Ethics and dissemination**			
Research ethics approval	24	Plans for seeking research ethics committee/institutional review board (REC/IRB) approval	__13___________
Protocol amendments	25	Plans for communicating important protocol modifications (eg, changes to eligibility criteria, outcomes, analyses) to relevant parties (eg, investigators, REC/IRBs, trial participants, trial registries, journals, regulators)	___13__________

For peer review only - http://bmjopen.bmj.com/site/about/guidelines.xhtml

Consent or assent	26a	Who will obtain informed consent or assent from potential trial participants or authorised surrogates, and how (see Item 32)	___9, 13______
	26b	Additional consent provisions for collection and use of participant data and biological specimens in ancillary studies, if applicable	__14_________
Confidentiality	27	How personal information about potential and enrolled participants will be collected, shared, and maintained in order to protect confidentiality before, during, and after the trial	______13,14____
Declaration of interests	28	Financial and other competing interests for principal investigators for the overall trial and each study site	_____15__________
Access to data	29	Statement of who will have access to the final trial dataset, and disclosure of contractual agreements that limit such access for investigators	______13_________
Ancillary and post-trial care	30	Provisions, if any, for ancillary and post-trial care, and for compensation to those who suffer harm from trial participation	_____13__________
Dissemination policy	31a	Plans for investigators and sponsor to communicate trial results to participants, healthcare professionals, the public, and other relevant groups (eg, via publication, reporting in results databases, or other data sharing arrangements), including any publication restrictions	___14___________
	31b	Authorship eligibility guidelines and any intended use of professional writers	_____14__________
	31c	Plans, if any, for granting public access to the full protocol, participant-level dataset, and statistical code	___14___________
**Appendices**			
Informed consent materials	32	Model consent form and other related documentation given to participants and authorised surrogates	__Submitted__
Biological specimens	33	Plans for collection, laboratory evaluation, and storage of biological specimens for genetic or molecular analysis in the current trial and for future use in ancillary studies, if applicable	__NA________

*It is strongly recommended that this checklist be read in conjunction with the SPIRIT 2013 Explanation & Elaboration for important clarification on the items. Amendments to the protocol should be tracked and dated. The SPIRIT checklist is copyrighted by the SPIRIT Group under the Creative Commons "Attribution-NonCommercial-NoDerivs 3.0 Unported" license.

For peer review only - http://bmjopen.bmj.com/site/about/guidelines.xhtml

BMJ Open

Strengthening contraceptive counselling services to empower clients and meet their needs: protocol for a 2-stage, multi-phase complex intervention in Pakistan and Nigeria

Journal:	*BMJ Open*
Manuscript ID	bmjopen-2023-081967.R3
Article Type:	Protocol
Date Submitted by the Author:	12-May-2024
Complete List of Authors:	Tran, Nguyen Toan; University of Technology Sydney,<br>Ali, Moazzam; World Health Organization, Reproductive Health and Research<br>Azmat, Syed Khurram ; Jinnah Sindh Medical University, AAPNA - Institute of Public Health; Marie Stopes Society Pakistan, Technical Services<br>Seuc, Armando; World Health Organization, Department of Reproductive Health and Research<br>Olaolorun, Funmilola; University of Ibadan College of Medicine, Community Medicine<br>Awan, Muhammad Ali ; AAPNA Institute of Public Health (AIPH), Jinnah Sindh, Medical University, Rafiqui H.J, Iqbal Shaheed Rd, Karachi, Sindh 75510, Pakistan<br>Morhason-Bello, Imran; University of Ibadan College of Medicine, Department of Obstetrics and Gynaecology, Faculty of Clinical Sciences, College of Medicine/University College Hospital, University of Ibadan<br>Thom, Ellen Mpangananji ; World Health Organization Country Office in Islammabad Pakistan<br>Martin , Josepth ; World Health Organization Country Office in Abuja, Nigeria<br>Abubakar, Hanifah Datti ; Mohammed Abdullahi Wase Teaching Hospital, Nasarawa<br>Uzma, Qudsia ; World Health Organization Country Office in Islamabad Pakistan<br>Kiarie, James; World Health Organization, Department of Reproductive Health and Research
**Primary Subject Heading**:	Reproductive medicine
Secondary Subject Heading:	Health services research
Keywords:	Clinical Trial, Decision Making, Health Services, Public health < INFECTIOUS DISEASES

Strengthening contraceptive counselling services to empower clients and meet their needs: protocol for a 2-stage, multi-phase complex intervention in Pakistan and Nigeria

Tran Nguyen Toan[1,2,3] nguyentoan.tran@uts.edu.au

Moazzam Ali[1*] alimoa@who.int

Syed Khurram Azmat[4] khurramazmat@gmail.com

Armando Seuc[1] armandoseuc@infomed.sld.cu

Funmilola M. Olaolorun PI[5] fmolaolorun@gmail.com

Muhammad Ali Awan[4] alighous@gmail.com

Imran Morhason-Bello[6] imranmorhasonbello@gmail.com

Ellen Mpangananji Thom[7] thome@who.int

Joseph Martin[8] mjoseph@who.int

Hanifah Datti Abubakar[9] hanniedee83@gmail.com

Qudsia Uzma[7] uzmaq@who.int

James Kiarie[1] kiariej@who.int

*Corresponding author: alimoa@who.int

[1] Department of Reproductive Health Research, World Health Organization, Avenue Appia 20, 1211 Genève 27, Switzerland.

[2] Faculty of Medicine, University of Geneva, Rue Michel-Servet 1, 1206 Genève, Switzerland

[3]Australian Centre for Public and Population Health Research, Faculty of Health, University of Technology, PO Box 123, Sydney, NSW 2007, Australia

[4] APPNA Institute of Public Health (AIPH), Jinnah Sindh, Medical University, Rafiqui H.J, Iqbal Shaheed Rd, Karachi, Sindh 75510, Pakistan

[5] Department of Community Medicine, College of Medicine, University of Ibadan, Nigeria

[6]Department of Obstetrics and Gynaecology, College of Medicine, University of Ibadan, Nigeria

For peer review only - http://bmjopen.bmj.com/site/about/guidelines.xhtml

[7] World Health Organization Country Office in Islamabad Pakistan

[8] World Health Organization Country Office in Abuja, Nigeria

[9] Mohammed Abdullahi Wase Teaching Hospital, Nasarawa, Kano, Nigeria

Abstract

Background: High-quality contraceptive counseling can accelerate global efforts to reduce the unmet need for and suboptimal use of modern contraceptives. This study aims to identify a package of interventions designed to strengthen in Pakistan and Nigeria and determine their effectiveness in increasing client-level decision-making, autonomy and meeting of contraceptive needs.

Methods: A multi-site, two-stage and five-phase intervention design will start with a pre-formative, formative, design, experimental and reflective phase. The Pre-formative phase will map potential study sites and establish the sampling frame. The two-part formative phase will first use participatory approaches to identify clients' perspectives, including young couples and providers, to ensure research contextualization and address each interest group's needs and priorities followed by clinical observations of client-provider encounters to document routine care. The design workshop in the third phase will result in the development of a package of contraceptive counseling interventions. In the fourth experimental phase, a multi-intervention, three-arm, single-blinded, parallel cluster randomized-controlled trial will compare routine care (arm 1) with the contraceptive counseling package (arm 2) and the same package combined with wider method availability (arm 3). The study aims to enrol a total of 7920 participants. The reflective phase aims to identify implementation barriers and enablers. The outcomes are clients' level of decision-making autonomy and use of modern contraceptives.

Ethics and dissemination: Ethical approval for this study was obtained from the WHO Scientific and Ethics Review Committee (Protocol ID Pakistan: ERC 006232 and Nigeria ERC: 006523). Each study site is required and has obtained the necessary ethical and regulatory approvals that are required in each specific country. Findings will be presented at local, national and international conferences and disseminated by peer-review publications.

Trial registration: Registered in ClinicalTrials.gov (NCT06081842, 31 October 2023).

Keywords

Counseling, contraception, family planning, empowerment, autonomy, qualitative research, cluster randomized controlled trial, health services research.

For peer review only - http://bmjopen.bmj.com/site/about/guidelines.xhtml

Strengths and limitations of this study

- A mixed-method study design offers valuable insights and can address research questions that quantitative or qualitative methods alone cannot adequately answer.
- Multi-center research offers several benefits compared to studies done at a single center such as access to a larger diverse sample, the ability to pool resources and foster connections and collaborations among researchers.
- The study will be implemented in a limited geography of the target countries; therefore, caution needs to be taken before generalising the findings.

Background

Overlooking women's and couples' need for quality contraceptive counseling constitutes a missed opportunity in family planning programs and services to ensure equitable access to the health, social, and economic benefits of modern contraception for women, men, and adolescents. Access to family planning is a human right enshrined in the United Nations' Sustainable Development Goal 3 on health and wellbeing and, specifically, its target 3.7: *ensure universal access to sexual and reproductive health-care services, including for family planning* [1]. Around 164 million women of reproductive age, have an unmet need for modern contraceptive methods despite increased contraceptive use over the past decades [2]. Satisfying the unmet need for contraception in low- and middle-income countries and offering all pregnant women and their newborns the standard care recommended by the World Health Organization (WHO) would result in dramatic reductions of unintended pregnancies by 68%, unsafe abortions by 72%, maternal deaths by 62%, and neonatal deaths by 69% [3]. Adequate contraceptive coverage among users can be improved by addressing suboptimal use and high discontinuation rates [4].

Effective contraceptive counseling can help individuals choose a method that meets their needs and preferences, manage any side effects, and continue using their preferred method or switch to an alternative one [5]. Contraceptive counseling could improve the effective use of modern contraception and reduce unmet need according to a systematic review that synthesized the evidence on the comparative effectiveness of different contraceptive counseling strategies [6]. The quality of contraceptive counseling in low-resource settings is often low. In Senegal, for example, only 18% of providers counseled their clients on the three-item Method Information Index (method use, side-effects, and timing of follow-up visits) [7]. Interventions targeting women requesting or initiating a chosen method, including structured counseling on side-effects, tend to show positive effects on contraceptive continuation [8-11]. Counseling interventions targeting family planning service users [12-16], abortion service users [17-19], postpartum women [20-25], and those reached by community-based interventions [26-28] have inconsistent effects on contraceptive behavior and satisfaction outcomes. In contrast, increased contraceptive use often follows interventions introducing systematic contraceptive counseling among women attending non-family planning outpatient services [29-31]. However, these interventions often include expanded contraceptive method provision, and the observed changes cannot be attributed to counseling alone. High-quality evidence is generally limited, and no clear consensus exists on how best to deliver contraceptive counseling to meet clients' contraceptive needs and enhance their satisfaction.

For peer review only - http://bmjopen.bmj.com/site/about/guidelines.xhtml

Building on this systematic review, the World Health Organization (WHO) organized a geographically diverse think tank in May 2019 in Geneva, Switzerland, to identify research and guidance gaps and other opportunities to improve the quality of contraceptive counseling [32]. Equally important was the crafting of a definition on contraceptive counseling, which was adopted by think tank members [33]: *Contraceptive counseling is defined as the exchange of information on contraceptive methods based on an assessment of the client's needs, preferences and lifestyle to support decision-making as per the client's intentions. This includes the selection, discontinuation or switching of a contraceptive method. The key principles are based on coercion-free and informed choice; neutral, understandable and evidence-based information; collaborative and confidential decision-making process; ensuring respectful care, dignity and choice.* The definition took into consideration the core dimensions articulated in the literature and mirrors the principles that were deliberated by the think tank: a two-way discussion and decision-making process between the client and provider, sharing objective and user-friendly information, and fundamentally grounded in the rights to autonomy, dignity, privacy, respect, and participation, among others. In addition, person-centeredness is a critical component of quality in family planning [5]. The 4-item person-centered contraceptive counselling scale (PCCC) is a valid and reliable measure of person-centered contraceptive counseling that reflects patients' perspectives on contraceptive counseling [34]. For example, integrating Dehlendorf's 4-item validated tool: PCCC: person-centered contraceptive counselling scale: Think about your visit. How do you think the member of the health care team did? Please rate them on each of the following by circling a number (the scale will use – Poor, Fair, Good, Very good and Excellent). Hence, to measure decision-making autonomy, the following considerations need to factor:

- Respecting me as a person
- Letting me say what mattered to me about my birth control method
- Taking my preferences about my birth control seriously
- Giving me enough information to make the best decision about my birth control method

Furthermore, the think tank members agreed on a prioritized set of guidance, knowledge, and implementation research gaps [32]. Such gaps include, for example, understanding or identifying clients' experiences with and expectations of counseling in a variety of global settings; the types of settings different populations, particularly adolescents, unmarried women, migrants, gender non-conforming people, and religious/ethnic minorities, receive most of their contraceptive counseling, and the quality of counseling across these settings; the fluidity/ambivalence of pregnancy intentions, risk perception (pregnancy and sexually transmitted infections), and women's understanding of their bodies; the independent effect of counseling from method provision; the core elements of high-quality of contraceptive counseling and how these differ by context and what provider characteristics affect counseling quality; the minimum skills and competencies for contraceptive counselors; the contents of a comprehensive package of best practices for FP counseling for different populations.

We have reviewed and used the currently available resources noted above such as Person-centered care and effective contraceptive counseling approaches, which focuses on persons' values and preferences, and are considered to improve the quality of care in family planning. These

For peer review only - http://bmjopen.bmj.com/site/about/guidelines.xhtml

approaches are likely to promote greater patient autonomy, trust, and satisfaction with services. Majority of the research has been based on small, qualitative studies, this study, using mixed methods approach, will benefit from existing resources and have also highlight the rights based approach in the counselling package to understand and document its implementation and effectiveness in the context of two countries.

Study overall objective and hypothesis

Owing to these gaps combined with the socio-cultural challenges related to accessing contraception, we propose a multi-site, two-stage and 5-phase study with two main objectives. The objective is to identify and test the effectiveness of a contraceptive counseling intervention bundle on improving clients' informed decision-making autonomy as well as meeting their need for family planning. The outcomes are changes in clients' decision-making autonomy and clients' met need for family planning. Secondary outcome will be change in modern contraceptive prevalence.

The research rests on the working hypotheses that the package will strengthen existing contraceptive counseling services; incorporate perspectives of key stakeholders such as clients and providers; align with national health policies and WHO global standards; and address challenges related to limited public resources.

Study countries: Pakistan and Nigeria

Pakistan

The study will be implemented in Pakistan in partnership with the Ministry of National Health Services, Regulations and Coordination.. The overall demographic, family planning, and reproductive health indicators based on the 2017-18 Demographic and Health Survey [35] note that rural settings fared worse than urban settings. The total fertility rate was 3.6 children per woman, modern contraceptive prevalance rate was 25% , overall unmet need for family planning was 17% and demand for family planning satisfied by a modern method 49%. In terms of family planning knowledge, more than 98% of urban and rural women had heard about any type of modern methods—before marriage, only 15% of women did. Regarding men's attitudes toward contraceptive use, 27% believed that family planning use is a woman's business, while 16% thought it may encourage women to be promiscuous [36].

Nigeria

The study will also be implemented in Nigeria by College of Medicine, University of Ibadan. The study will be conducted in two study sites: Lagos (southern part of Nigeria) and Kano (northern part of Nigeria) states. These states were selected for the following reasons: (1) Data from the Performance Monitoring for Action (PMA) program are available for both states from annual surveys over the past three years, including information on the method information index plus and other family planning indicators (pmadata.org); (2) The team has

For peer review only - http://bmjopen.bmj.com/site/about/guidelines.xhtml

a good network in both locations and will be able to readily collaborate with the State Ministries of Health; (3) Safety and security are relatively good with no serious threats in recent times.

The latest demographic, family planning, and reproductive health indicators based on the 2018 Demographic and Health Survey in Nigeria in 2019 showed that in general, rural settings fared worse than urban settings [37]. The total fertility rate was 5.3 children per woman; overall unmet need for family planning was 18.9%; demand for family planning satisfied by a modern method 33.9% and modern contraceptive prevalance rate was 12%. In terms of family planning knowledge, 93.9% of currently married women and 98.3% of sexually active unmarried women had heard about any type of modern contraceptive method.

Methods

Framework and perspectives

Theory of change

This health services research is guided by the theory of change framework and incorporates perspectives on human rights and the health system to develop a contraceptive counseling intervention package that strengthens existing family planning services and ensures effectiveness, safety, and cultural appropriateness. Following the research findings, the long-term vision is to support the health system in developing a sustainable and scalable contraceptive counselling intervention package for Nigeria and Pakistan. Although the exact package has not yet been developed, based on the requirement set by the respective ministry of health/population in Pakistan and Nigeria, the researchers are willing to contribute to the development of provider training, post-training mentorship and the provision of IEC materials to enable the implementation of the intervention bundle.

The theory of change underpins the staged and phased strategy utilized in this trial, which includes key objectives, activities, and intermediate and final results (refer to Figure 1). [38].

For peer review only

For peer review only - http://bmjopen.bmj.com/site/about/guidelines.xhtml

FIGURE 1 HERE

Health system perspective

First and related to the research model outlined in Figure 1, the study will rely on the WHO Health System Framework with its six system building blocks and link research and programmatic considerations for contraceptive counseling [39]. The six foundational components of health systems are leadership/governance, health care financing, health workforce, medical products and technologies, information and research, and service delivery [39].

Human rights perspective

The research will use the WHO Framework for Ensuring Human Rights in the Provision of Contraceptive Information and Services [40]. The human rights framework comprises nine standards that interweave with the dimensions of quality of care: quality, acceptability, availability, accessibility, non-discrimination, participation, accountability, informed-decision making, and privacy and confidentiality. The research team would ensure a person-oriented approach where no teenage girls or women are discriminated against (for example, in receiving family planning information and services) because of their marital status, age, HIV status, minority status, or other social or medical factors [33].

With regard to the participation standard, the research will ensure active and informed participation of individuals in decision-making that affects them, including on matters related to their health [41]. Due to the complex social, cultural, and religious challenges and opportunities surrounding contraceptive use, our research design will assume that the following participatory action research (or participatory research) principles will be essential to meet the study objectives as well as gain insights into the implementation process. [42].

Study phases and timelines

The research entails a complex multi-site, two-stage and 5-phase (Figure 1): STAGE 1 is formative and will comprise of a pre-formative (phase 1) and a formative (phase 2). While the STAGE 2 is experimental and will include intervention design (phase 3), experimental (phase 4), and reflective (phase 5). The methods proposed for each phase are not inherently complex; however, the intervention in its globality is complex due to the number and difficulty of behaviors required by both the intervention deliverers and recipients, and the involvement of multiple organizational levels or groups targeted by the intervention, among other factors [43]. The design adheres to recommendations from the Medical Research Council (2008/2010) for developing a complex intervention, assessing its feasibility, and facilitating its implementation and evaluation. [43]. As such, the phases are interlinked and have key objectives, methodologies, and outcomes.

STAGE 1: the pre-formative phase aims to establish a sampling frame of potential study sites by mapping primary health centers based on set criteria. Eligibility criteria for health

For peer review only - http://bmjopen.bmj.com/site/about/guidelines.xhtml

centers include: (1) provision of family planning services; (2) availability of at least three to four modern contraceptive methods, including a barrier method (e.g., condom) and short- and long-term methods (e.g., pills, injectables, implants, intra-uterine devices); (3) absence of contraceptive stock-outs over the preceding three months in alignment with national policy; (4) a minimum average of 25 family planning encounters per week (or 100 per month) at selected high-performing facilities (both public and private), and at least 12 encounters per week (or 48 per month) at selected low-performing facilities (again, both public and private); (5) non-receipt of family planning technical support (e.g., capacity building, supplies) from other organizations at recruitment, with a commitment to refrain from such support during the study; and (6) willingness to participate in the study. Based on this sampling frame, four health centers (public and private sector) will be allocated to the formative phase while the others will be randomized to each arm of the experimental phase. The specific objectives of the ***formative phase*** are to (1) document the in-clinic contraceptive counseling routine using observation checklists and (2) seek through qualitative research the perspectives of clients, including young people, providers, and other stakeholders to ensure adequate contextualization of the research and address needs and priorities of each interest group regarding contraceptive counseling barriers and enablers.

STAGE 2: The specific objective of the ***intervention design phase*** is to co-design, based on the results of the formative phase and through a participatory and consensus-building approach engaging clients, providers, and other main actors, a package of contraceptive counseling to be implemented during the experimental phase. To be integrated into the final package, interventions will need to consider current human resources and clinical capacity, costs, acceptability, feasibility, scalability, and other field considerations.

The objective of the ***experimental phase*** is to assess, using a cluster randomized controlled trial (RCT) design, the impact of the contraceptive counseling bundle on clients' level of decision-making autonomy and demand satisfied for family planning. The three-armed cluster RCT comprise (1) a control arm with contraceptive counseling and care and a range of family planning methods that are available at field level per the national policy and guidelines; (2) an experimental arm implementing the contraceptive counseling package but still with a range of family planning methods that are available (upto 4 methods) per the national policy and guidelines at field level; and (3) another experimental arm with the contraceptive counseling package but with an expanded range of family planning methods (more than 4 methods) that are recommended by national policy and guidance.

The ***reflective phase*** objective is to determine operational barriers and catalysts related to the package implementation using qualitative research. Once the package of interventions has been identified—and resources permitting, we might consider conducting a cost-effectiveness analysis to determine the counseling package cost-effectiveness.

The qualitative research and cluster RCT are detailed in the following sections.

Timelines

The study be tenataively initiated in November 2023 and it will be completed by November 2025. The analysis and report will be drafted by June 2026.

For peer review only - http://bmjopen.bmj.com/site/about/guidelines.xhtml

Qualitative research

General design

For the qualitative research components of the study during the formative and reflective phases, we will conduct in-depth interviews (IDIs) in Pakistan and Nigeria with various community members (including women, adolescent males and females, partners, and influential individuals), health personnel (such as clinicians, managers, and Community Health Workers – CHWs), development partners, donor agencies, and national policymakers. The formative phase will involve selecting four (4) health centers (both public and private) and their respective coverage areas in each country using a convenient sampling approach. These sites will be chosen from the sampling frame established during the pre-formative phase.

To identify barriers and facilitators related to contraceptive counseling, separate IDIs will be conducted with groups of six to ten participants from the following categories: (1) women who have used family planning services at the health center within the past 12 months: married and unmarried adolescent females (≤ 19 years), married and unmarried women 19-39 years, married and unmarried women ≥ 40 years [The study will include Women of Reproductive Age (WRA) – irrespective of their marital status and age group (including migrants refugee people, ethnic and religious minorities, women with disabilities, women or men with HIV etc. are inclusive] ; (2) married and unmarried men who are husbands or partners of clients who have used family planning services at the clinic within the past 12 months, and married and unmarried adolescent males (<19 years); and (3) family planning service providers.

About the recruitment of men/male participants, the women participants will be first recruited based on their eligibility and recruitment criteria. If the female participant is selected, she will be asked whether she is using the FP method in consensus with her male partner/spouse or using it covertly. If she uses the FP method with the consensus of her male partner/spouse, we will recruit the accompanying male partners/spouses in the study. If the women participant is using the FP method covertly, then the women will be first asked to provide consent to be invited to study participation. If she does not consent, then men/male participants will be identified by the CHWs at the community level. In both cases, formal consent will be taken from the woman whether she is using the FP method covertly or in consensus with her male partner/spouse.

IDIs will also take place with clinic managers, supervisors, CHWs, policy makers, and other influential individuals/opinion leaders (selected purposively) who have observed, supervised, or monitored family planning services at/or the catchment of the selected public/private health facility. We anticipate that interviews will last between 45-60 mins and will take place in a safe, neutral space of the participant's choosing (e.g. town hall, office space, on porch outside participant's home, etc.). Both the recruiter and the participant will decide and agree on the location of the interview and this will be conveyed to the research team. Alternatively, the team will also identify pay-per-hour spaces as an option to present to the participant. The interview guides will address several key themes pertinent to contraceptive counseling, including: practices, attitudes, and knowledge regarding contraception; health systems and socio-cultural challenges and enablers affecting contraceptive use; and quality of care, encompassing autonomy, informed decision-making, and other human rights considerations in contraceptive services.

For peer review only - http://bmjopen.bmj.com/site/about/guidelines.xhtml

These themes will guide the exploration of participants' perspectives and experiences related to contraceptive counseling within the broader context of reproductive health services.

The qualitative research in the ***reflective phase*** will occur in three health centers in each of the three study arms (nine centers in total). The centers will be purposely sampled to include the lowest, average, and highest performing ones in terms of measured outcomes to enable the broadest possible understanding. Aligned with the theory of change framework, the qualitative research will conduct an implementation evaluation to examine challenges and facilitators associated with the provision of contraceptive counseling and family planning services, assess fidelity to the intervention within experimental groups, and explore strategies for potential scalability. IDIs will be conducted separately with service providers, clients across different age groups (≤ 19 years, 20-39 years, ≥ 40 years), and clinic managers. The interview guides will address the following topics related to contraceptive counseling: quality of care including autonomy in decision-making and other human rights perspectives, operational challenges and facilitators, and considerations for scaling up the intervention

Selection criteria and recruitment approach: A standard process of selection and consent-taking will be undertaken at each study site comprising potential study participants for each of the above categories of individuals (including vulnerable populations). A recruitment approach will follow, and the main actors who will facilitate the recruitment process of the potential target respondents for this research are a) selected service providers, b) Community Health Workers – working in the catchment area of the selected service providers, c) community-based opinion leaders, i.e., male, and female schoolteachers, social activist/ NGO workers. The country PI/Co-PI will train all of them on the study's main objectives, purpose, issues in ethics and the importance of consent.

Qualitative data management and analysis

At the country level, the study will engage a team of reseachers with expertise in qualitative methods and local dialects.

The interviews will occur in a private location guaranteeing confidentiality. We anticipate that interviews will last between 45-60 mins and will take place in a safe, neutral space of the participant's choosing (e.g. town hall, office space, on porch outside participant's home, etc.). The interviews will be recorded with the participants' informed consent. Research assistants proficient in transcription will transcribe and translate the audio recordings into English. Accuracy checks will be conducted by comparing the transcripts with the original audio files.

To conduct thematic analysis, qualitative research management software like QSR NVivo 12 or Atlas.ti will be utilized. This involves creating a primary codebook to outline all nodes and using it to code the collected data. As analysis progresses, new nodes will be incorporated into the codebook to capture emerging themes and concepts identified during coding. The data will be coded by research assistants skilled in using Nvivo. To ensure coding quality, inter-rater

For peer review only - http://bmjopen.bmj.com/site/about/guidelines.xhtml

reliability will be assessed by calculating the Cohen's Kappa coefficient, with a threshold of 0.8 or higher considered acceptable for concordance.

During the intervention design workshop, the findings from the formative phase will be shared and discussed with key research team members, clinicans, and clients. The aim is to inform the development of a contextually sensitive package of contraceptive counseling interventions from a bottom-up approach. Each workshop participant's perspectives will be taken into account when assessing the potential effectiveness, feasibility, acceptability to women and providers, integration into family planning programs, sustainability, and scalability of potential interventions.

Cluster randomized controlled trial

The research will be a multi-intervention, three-arm, single-blinded, parallel, cluster RCT done in selected primary health centers (clusters) in Pakistan and in Nigeria (see **Figure 2** for the cluster RCT flow diagram, and **Figure 3** for the schedule of enrolment, interventions, and assessments (SPIRIT template). Centers will be randomly allocated to the three study arms in matched ratios (1:1:1) on the basis of the number of monthly family planning encounters, the number of available contraceptive types, the ratio of health workers per population in the clinic coverage zone, the location in urban or rural settings, and selected district-level variables that may have an influence on the study outcomes, including the unmet need for family planning, the level of literacy among women, and household income terciles. Only data analysts will be masked/blinded to trial-arm allocation. Service providers skilled in family planning services will implement the clinical components of the contraceptive counseling package.

FIGURE 2 HERE

FIGURE 3 HERE

Outcome measures

Quantitative indicators will be captured in case-report forms (CRFs) developed and piloted at the country level before finalization. The proposed outcomes to be assessed after each visit, at mid-point (6 months after enrollment), and at the study end (12 months after enrollment) are oriented toward clients and consist of levels of clients' decision-making autonomy and demand satisfied for family planning.

To determine their level of decision-making autonomy, clients will be asked to answer questions about the different domains contained in the counseling definition (see Background). These domains comprise client-provider collaboration, client's needs and preferences, the quality of information, freedom from coercion, confidentiality, and dignity. The Person-Centered Contraceptive Counseling scale (PCCC), developed and tested in publicly-funded clinics in

For peer review only - http://bmjopen.bmj.com/site/about/guidelines.xhtml

California will also be used [43]. The four PCCC questions fall under the domains of respect, eliciting—and giving serious consideration to—client's preferences, and information quality and it will be assessed during the intervention phase as per described by UNFPA to measure decision-making autonomy SDG 5.6.1 [44].

The study proposes nonetheless the another outcome, while acknowledging its limitations: modern contraceptive prevalence after the first visit and at 6 and 12 months after enrollment.

Eligibility criteria for clusters (health centers) and participants

The trial will take place in specific primary health centers that meet the eligibility criteria outlined in the pre-formative phase objectives. A woman can participate in the study if

(1) she comes to the family planning clinic with the intention to (i) use contraception for the first time in her life (new user), or (ii) switch from a contraceptive method to another one (switching user), or (iii) resume a method after not using any in the prior three months (lapse user), or (iv) discontinue a modern method (discontinuing user);

(2) she is not coming for the resupply of a currently used method, such as pills or injectables;

(3) she has the intention to continue her follow-up at the health center during the 12-month study follow-up;

(4) she does not participate in another study; and

(5) she provides informed consent.

Exclusion Criteria: Not willing to participate in the complete duration of the trial.

Interventions

The primary health centers assigned to the first experimental group will implement the intervention package. Meanwhile, those in the second experimental group will implement the intervention package along with an expanded range of family planning methods recommended by national guidelines. The control group will continue with usual care practices.

Depending on the specific components of the intervention package, certain interventions, such as provider capacity building, may be implemented prior to participant recruitment to ensure readiness and effectiveness of the interventions during the study period.

For peer review only - http://bmjopen.bmj.com/site/about/guidelines.xhtml

Randomization

In each country, a total of 36 sites will be selected and matched into 12 trios based on the specified criteria. Within each trio, the study sites will be randomly assigned to one of three study arms at a 1:1:1 ratio. This randomization process will be repeated 12 times, once for each trio. The randomization process will not have any restrictions, and blinding (masking) of participants and providers cannot be implemented except for data analysts who will handle the study data independently. The study clusters will include all consecutive and eligible participants until the total sample size of 7920 participants is reached.

Data source and collection

Data pertaining to key outcome measures and quantitative process indicators will be gathered using either paper-based or electronic CRFs. The CRFs will be developed by the WHO team in Geneva in collaboration with the country research teams. To refine the forms, advanced drafts will undergo testing with a suitable sample of service providers and simulated clients from the formative phase sites. Each health center participating in the RCT will be assigned a research assistant who will receive training on adhering to the study manual and standard operating procedures for data management, developed by the WHO and consistent across both study countries. To ensure data quality, the accuracy and completeness of information recorded on the CRFs will undergo rigorous checks at multiple levels by field coordinators and data managers. This systematic approach will help maintain data integrity throughout the study period**.**

Data management

In line with protocols used in other multicenter studies sponsored by the UNDP/UNFPA/UNICEF/WHO/World Bank Special Programme of Research, Development and Research Training in Human Reproduction (HRP) at WHO in Geneva, a web-based data management system with be used for data entry and quality monitoring. HRP's Biostatistics and Data Management team will develop this system using OpenClinica (version 3.16), an electronic data capture software designed for clinical research. OpenClinica includes programmed edit checks to ensure data quality, completeness, and consistency, which helps in reducing delays associated with data queries and problem resolution, ensuring easy accessibility of the dataset. The OpenClinica system is a secure, open-source, and reputable web-based software solution that fully adheres to Good Clinical Practice (GCP) and regulatory guidelines, as well as the HRP/WHO Standard Operating Procedures for clinical trial management

The country research teams in Pakistan and Nigeria will enter the data locally and have it reviewed by the WHO team in Geneva. We will provide training to the country teams on data entry and management using OpenClinica. The country research teams will be tasked with data verification, uploading data to shared files, and entering it into the OpenClinica system. To enhance accuracy, double data entry will be performed. The system is equipped with automated data query features to identify missing values, outliers, inconsistencies, and other errors. The WHO team will oversee data quality monitoring. Any questions regarding data inconsistencies or missing values will be addressed through standardized forms sent to the sites, with continuous

For peer review only - http://bmjopen.bmj.com/site/about/guidelines.xhtml

resolution of issues as they arise. To ensure data integrity and participant confidentiality, data transmission will be encrypted. Only approved users will be given access to the data, which will be password protected.

Statistical analysis

We will prepare a comprehensive statistical analysis plan. Participants will form the unit of analysis and the clustered nature of the data will be accounted for. All analyses will be by intention to treat. For continuous variables, we will compute means, standard deviations, minimum and maximum values, and for categorical variables, we will compute frequencies and percentages. We will look at the distribution of variables and see if there are any outliers as part of data quality control and descriptive analysis. Descriptive statistics will be tabulated for individual clusters and aggregated across clusters. Within each of the three groups/arms of the study (two experimental and one control), outcome differences between final (12 months after enrolment) and intermediate (6 months after enrolment) outcome measurements will be obtained, and these changes will be compared between the control group and each of the two experimental groups. For categorical outcomes, chi-square tests for two independent proportions will be used, and for continuous variables, t-student tests for two independent means will be used. We will use IBM SPSS Statistics 25, R 3.6.2, STATA 16.1, or SAS 9.4 statistical packages for the analysis. The findings will be presented using the CONSORT Statement checklist from 2010 and its extension for cluster studies [45].

Patient and public involvement

Patients or the public were not involved in the design, or conduct, or reporting, or dissemination plans of this research.

Ethics and regulatory aspects

We adhered to The Ottawa Statement on the Ethical Design and Conduct of Cluster Randomized Trials when designing this experiment [46]. The Research Project Review Panel (RP2) of the HRP peer-reviewed the protocol, and RP2 and the WHO Research Ethics Review Committee, Geneva, Switzerland (Pakistan: ERC 006232 and Nigeria ERC: 006523), approved it and so did the IRB of Research and Development Solutions (IRB No:00010843) in Pakistan. While the Ministry of Health, Kano State of Nigeria (NHREC 17/03/2018) and Lagos Research Ethics Committee in Nigeria (LREC/06/10/2180) provided ethical approvals for the Nigeria study .

This research is grounded in human rights principles, specifically participation in the form of participatory action research. The existing literature suggests promise for the success of our proposed approach. For instance, a synthesis of evidence on community-based participatory research across various settings has shown that collaboration among community partners, researchers, and organizations has led to community-level actions that improve health and well-being while reducing health disparities [47]. This collaborative process has also contributed to strengthening community capacity in research and leadership skills. Another review highlighted that participatory action research can ensure that research is culturally and logistically appropriate, enhance recruitment capacity, foster professional capacity and competence among

For peer review only - http://bmjopen.bmj.com/site/about/guidelines.xhtml

stakeholder groups, facilitate productive conflicts followed by constructive negotiation, improve the quality of outputs and outcomes over time, enhance sustainability of project goals beyond funding periods and during gaps in external funding, and drive system changes and new unforeseen projects and activities [48].

Dissemination

The findings from the analysis of this study will be disseminated in a variety of ways. At country level, the results will be presented to policy makers, researchers, managers through policy briefs and workshops in collaboration with the WHO office. The results will also be disseminated among the communities/participants through online platforms/text messages, which will be provided to the participants by the data collectors/health workers. In addition, the results will also be presented to the scientific and funding community in collaboration with WHO country and regional offices, by communication and manuscript publications in peer-reviewed international journals.

Declarations

Ethics approval and consent to participate

The research protocol received approval from the Research Project Review Panel (RP2) of the UNDP/UNFPA/UNICEF/WHO/World Bank Special Programme of Research, Development and Research Training in Human Reproduction (HRP) at WHO in Geneva, as well as from the WHO Research Ethics Review Committee in Geneva, Switzerland. Ethics approval for the study was also obtained from the ethics review committees in Pakistan and Nigeria. All adult participants will provide written consent to participate in the study. Non-adult participants will provide informed assent, and their guardians or parents will provide informed consent on their behalf.

Consent for publication:

Not applicable.

Availability of data and materials

Not applicable.

Competing interests

The authors declare that they have no competing interests.

Patient and public involvement Patients and/or the public were not involved in the design, or conduct, or reporting, or dissemination plans of this research.

For peer review only

For peer review only - http://bmjopen.bmj.com/site/about/guidelines.xhtml

Funding

The project site in Pakistan has been supported by the UNDP-UNICEF-WHO-World Bank Special Programme of Research, Development and Research Training in Human Reproduction Programme (HRP grant No. TRIMS: A66026); a co-sponsored programme executed by the World Health Organisation (WHO). The project in Nigeria generously received funding from United States Agency for International Development (USAID grant No. GHA-G-00-09-00003). The funders will take no role in subjects' recruitment, data collection, analyses, or interpretation, and will take no part in the decision to publish.

Author's contributions

MA, TNT, and AS designed the initial study concept, the study protocol, and study instruments. SKA,MA, MAA and FMO provided revisions to multiple versions of the study protocol based on the feedback from WHO-ERC. SKA, MA, FMO, MAA, EMT, JM, QU, JK,HDA and IMB contributed to the final study protocol and study instruments. TNT wrote the first draft of the manuscript with the contributions of MA, AS, and KJ. While, SKA and MA developed the final draft of the manuscript. All authors read and approved the final manuscript.

Acknowledgements

The authors are grateful to Dr. Onikepe Owolabi of the Guttmacher Institute and Dr. Nathalie Roos of the Karolinska Institute for their technical support and insights.

ORCID:

Moazzam Ali http://orcid.org/0000-0001-6949-8976

Syed Khurram Azmat https://orcid.org/0000-0003-0158-8734

Tran Nguyen Toan: https://orcid.org/my-orcid?orcid=0000-0001-7134-7878

References

1. Howden-Chapman P, Siri J, Chisholm E, Chapman R, Doll CN, Capon A: **SDG 3: Ensure healthy lives and promote wellbeing for all at all ages**. *A guide to SDG interactions: from science to implementation Paris, France: International Council for Science* 2017:81-126.
2. United Nations Department of Economic and Social Affairs, Population Division (2022). World Family Planning 2022: Meeting the changing needs for family planning: Contraceptive use by age and method.UN DESA/POP/2022/TR/NO. 4
3. Sully E, Biddlecom A, Darroch J, Ashford L, Lince-Deroche N, Riley T: **Adding It Up: Investing in Sexual and Reproductive Health, 2020**. *New York, NY: Guttmacher Institute*.

For peer review only - http://bmjopen.bmj.com/site/about/guidelines.xhtml

4. Polis CB, Bradley SE, Bankole A, Onda T, Croft T, Singh S: **Contraceptive failure rates in the developing world: an analysis of demographic and health survey data in 43 countries**. *New York: Guttmacher Institute* 2016.

5. Dehlendorf C, Krajewski C, Borrero S: **Contraceptive counseling: best practices to ensure quality communication and enable effective contraceptive use**. *Clinical obstetrics and gynecology* 2014, **57**(4):659.

6. Cavallaro FL, Benova L, Owolabi OO, Ali M: **A systematic review of the effectiveness of counselling strategies for modern contraceptive methods: what works and what doesn't?** *BMJ Sexual & Reproductive Health* 2019.

7. Assaf S, Wang W, Mallick L: **Provider Counseling and Knowledge Transfer in Health Facilities of Haiti, Malawi, and Senegal. DHS Analytical Studies No. 60**. *Rockville, Maryland* 2016.

8. De Cetina TEC, Canto P, Luna MO: **Effect of counseling to improve compliance in Mexican women receiving depot-medroxyprogesterone acetate**. *Contraception* 2001, **63**(3):143-146.

9. Modesto W, Bahamondes MV, Bahamondes L: **A randomized clinical trial of the effect of intensive versus non-intensive counselling on discontinuation rates due to bleeding disturbances of three long-acting reversible contraceptives**. *Hum Reprod* 2014, **29**(7):1393-1399.

10. Patel D, Koenig M, Patel A, Rani M: **The impact of improved service quality on IUD continuation: evidence from rural India**. *JOURNAL OF FAMILY WELFARE* 2003, **49**:1-9.

11. Amatya R, Akhter H, McMahan J, Williamson N, Gates D, Ahmed Y: **The effect of husband counseling on NORPLANT® contraceptive acceptability in Bangladesh**. *Contraception* 1994, **50**(3):263-273.

12. Jain AK, RamaRao S, Kim J, Costello M: **Evaluation of an intervention to improve quality of care in family planning programme in the Philippines**. *Journal of biosocial science* 2012, **44**(1):27.

13. Chin-Quee DS, Janowitz B, Otterness C: **Counseling tools alone do not improve method continuation: further evidence from the decision-making tool for family planning clients and providers in Nicaragua**. *Contraception* 2007, **76**(5):377-382.

14. Sanogo D, RamaRao S, Jones H, N'diaye P, M'bow B, Diop CB: **Improving quality of care and use of contraceptives in Senegal**. *African journal of reproductive health* 2003:57-73.

15. Sapkota S, Rajbhandary R, Lohani S: **The impact of balanced counseling on contraceptive method choice and determinants of long acting and reversible contraceptive continuation in Nepal**. *Maternal and child health journal* 2017, **21**(9):1713-1723.

16. Kim YM, Putjuk F, Basuki E, Kols A: **Increasing patient participation in reproductive health consultations: an evaluation of "Smart Patient" coaching in Indonesia**. *Patient education and counseling* 2003, **50**(2):113-122.

17. Zhu JL, Zhang W-H, Cheng Y, Xu J, Xu X, Gibson D, Støvring H, Claeys P, Temmerman M: **Impact of post-abortion family planning services on contraceptive use and abortion rate among young women in China: a cluster randomised trial**. *The European Journal of Contraception & Reproductive Health Care* 2009, **14**(1):46-54.

18. Ferreira ALCG, Souza AI, Pessoa RE, Braga C: **The effectiveness of contraceptive counseling for women in the postabortion period: an intervention study**. *Contraception* 2011, **84**(4):377-383.

19. Smith C, Ngo TD, Gold J, Edwards P, Vannak U, Sokhey L, Machiyama K, Slaymaker E, Warnock R, McCarthy O: **Effect of a mobile phone-based intervention on post-abortion contraception: a randomized controlled trial in Cambodia**. *Bulletin of the World Health Organization* 2015, **93**:842-850A.

20. Kaewkiattikun K: **Effects of immediate postpartum contraceptive counseling on long-acting reversible contraceptive use in adolescents**. *Adolescent health, medicine and therapeutics* 2017, **8**:115.

For peer review only

For peer review only - http://bmjopen.bmj.com/site/about/guidelines.xhtml

21. Bolam A, Manandhar DS, Shrestha P, Ellis M, Anthony MdL: **The effects of postnatal health education for mothers on infant care and family planning practices in Nepal: a randomised controlled trial**. *Bmj* 1998, **316**(7134):805-811.
22. Adanikin AI, Onwudiegwu U, Loto OM: **Influence of multiple antenatal counselling sessions on modern contraceptive uptake in Nigeria**. *Eur J Contracept Reprod Health Care* 2013, **18**(5):381-387.
23. Vural F, Vural B, Cakiroglu Y: **The effect of combined antenatal and postnatal counselling on postpartum modern contraceptive use: prospective case-control study in Kocaeli, Turkey**. *Journal of clinical and diagnostic research: JCDR* 2016, **10**(4):QC04.
24. Fatima P, Antora AH, Dewan F, Nash S, Sethi M: **Impact of contraceptive counselling training among counsellors participating in the FIGO postpartum intrauterine device initiative in Bangladesh**. *International Journal of Gynecology & Obstetrics* 2018, **143**:49-55.
25. Ndegwa SW, Gichuhi J, Qureshi Z, Lubano K: **The effect of two levels of counselling on acceptance, uptake and early outcomes of post-placental intra-uterine contraceptive device**. *East African medical journal* 2014, **91**(12):449-456.
26. Terefe A, Larson CP: **Modern contraception use in Ethiopia: does involving husbands make a difference?** *American Journal of Public Health* 1993, **83**(11):1567-1571.
27. El-Khoury M, Thornton R, Chatterji M, Kamhawi S, Sloane P, Halassa M: **Counseling women and couples on family planning: a randomized study in Jordan**. *Studies in family planning* 2016, **47**(3):222-238.
28. Lemani C, Tang JH, Kopp D, Phiri B, Kumvula C, Chikosi L, Mwale M, Rosenberg NE: **Contraceptive uptake after training community health workers in couples counseling: A cluster randomized trial**. *PloS one* 2017, **12**(4):e0175879.
29. Gillespie D, Bradley H, Woldegiorgis M, Kidanu A, Karklins S: **Integrating family planning into Ethiopian voluntary testing and counselling programmes**. *Bulletin of the World Health Organization* 2009, **87**:866-870.
30. Lee J, Papic M, Baldauf E, Updike G, Schwarz EB: **A checklist approach to caring for women seeking pregnancy testing: effects on contraceptive knowledge and use**. *Contraception* 2015, **91**(2):143-149.
31. Yassin AS, Cordwell D: **Does dedicated pre-abortion contraception counselling help to improve post-abortion contraception uptake?** *BMJ Sexual & Reproductive Health* 2005, **31**(2):115-116.
32. Ali M, Tran NT, Kabra R, Kiare J: **Strengthening contraceptive counselling: gaps in knowledge and implementation research**. In.: British Medical Journal Publishing Group; 2021.
33. Ali M, Tran NT: **Defining counselling in contraceptive information and services: outcomes from an expert think tank**. In.: British Medical Journal Publishing Group; 2021.
34. Dehlendorf C, Fox E, Silverstein IA, Hoffman A, Pérez MPC, Holt K, Reed R, Hessler D: Development of the Person-Centered Contraceptive Counseling scale (PCCC), a short form of the Interpersonal Quality of Family Planning care scale. Contraception 2021.
35. National Institute of Population Studies: **Pakistan Demographic and Health Survey 2017–18. Available at: https://dhsprogram.com/pubs/pdf/FR290/FR290.pdf (2019). Accessed 1 April 2021.**
36. National Institute of Population Studies: **Pakistan Special Demographic and Health Survey 2019. Available at: https://dhsprogram.com/publications/publication-SR267-Summary-Reports-Key-Findings.cfm. Accessed 1 April 2021**
37. National Population Commission (NPC) [Nigeria] and ICF. 2019. **Nigeria Demographic and Health Survey 2018**. Abuja, Nigeria, and Rockville, Maryland, USA: NPC and ICF

For peer review only - http://bmjopen.bmj.com/site/about/guidelines.xhtml

38. De Silva MJ, Breuer E, Lee L, Asher L, Chowdhary N, Lund C, Patel V: **Theory of Change: a theory-driven approach to enhance the Medical Research Council's framework for complex interventions**. *Trials* 2014, **15**:267.

39. World Health Organization: **Everybody's business--strengthening health systems to improve health outcomes: WHO's framework for action**. Geneva: World Health Organization; 2007. https://www.who.int/healthsystems/strategy/everybodys_business.pdf. Accessed 15 May 2020.

40. World Health Organization: **Framework for ensuring human rights in the provision of contraceptive information and services**. In. Edited by World Health Organization. Geneva: World Health Organization,; 2014.

41. Committee on Economic Social and Cultural Rights (22nd session): **General comment No 14: The right to the highest attainable standard of health–Article 12 of the International Covenant on Economic, Social and Cultural Rights (CESCR)**. *Geneva: United Nations Economic and Social Council* 2000.

42. Macaulay AC, Commanda LE, Freeman WL, Gibson N, McCabe ML, Robbins CM, Twohig PL: **Participatory research maximises community and lay involvement**. *Bmj* 1999, **319**(7212):774-778.

43. Craig P, Dieppe P, Macintyre S, Michie S, Nazareth I, Petticrew M: **Developing and evaluating complex interventions: new guidance. Medical Research Council, 2008**. In.; 2010.

44. UNFPA and Hera (2019). Research on what determines women's ability to decide on their SRHR and the relationship between this and other aspects of their lives. Volumes 1 and 2: Final report, October 2019. https://www.unfpa.org/sites/default/files/resource-pdf/20-033_SDG561-IssueBrief-v4.1.pdf. Accessed on 05 March 2024

45. Zwarenstein M, Treweek S, Gagnier JJ, Altman DG, Tunis S, Haynes B, Oxman AD, Moher D: **Improving the reporting of pragmatic trials: an extension of the CONSORT statement**. *Bmj* 2008, **337**:a2390.

46. Weijer C, Grimshaw JM, Eccles MP, McRae AD, White A, Brehaut JC, Taljaard M: **The Ottawa Statement on the Ethical Design and Conduct of Cluster Randomized Trials**. *PLoS Med* 2012, **9**(11):e1001346.

47. Salimi Y, Shahandeh K, Malekafzali H, Loori N, Kheiltash A, Jamshidi E, Frouzan AS, Majdzadeh R: **Is Community-based Participatory Research (CBPR) Useful? A Systematic Review on Papers in a Decade**. *International Journal of Preventive Medicine* 2012, **3**(6):386-393.

48. Jagosh J, Macaulay AC, Pluye P, Salsberg J, Bush PL, Henderson J, Sirett E, Wong G, Cargo M, Herbert CP: **Uncovering the benefits of participatory research: implications of a realist review for health research and practice**. *The Milbank Quarterly* 2012, **90**(2):311-346.

For peer review only - http://bmjopen.bmj.com/site/about/guidelines.xhtml

Figures, tables, additional files

Figure 1. Overall study model.

Figure 2. Flow diagram of the cluster randomized control trial in each study country.

Figure 3. Schedule of enrolment, interventions, and assessments for the cluster randomized controlled trial phase in each study country (SPIRIT template).

For peer review only

For peer review only - http://bmjopen.bmj.com/site/about/guidelines.xhtml

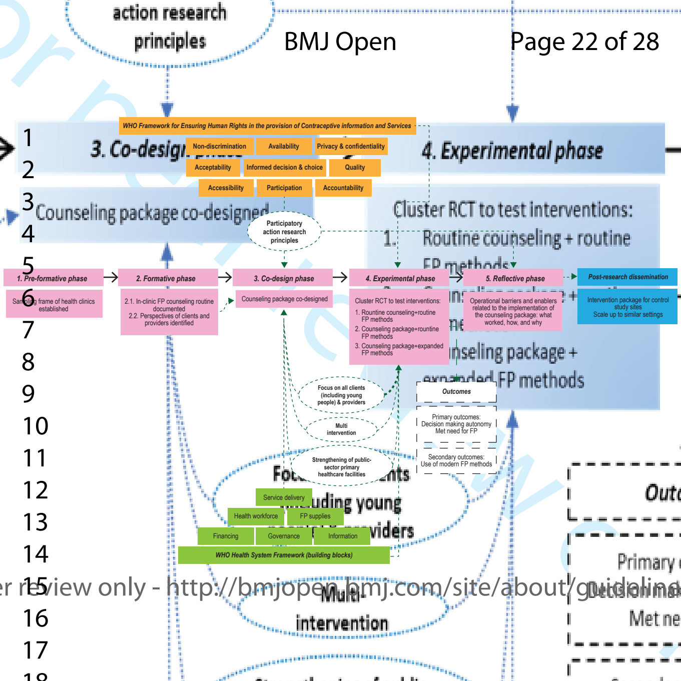

For peer review only - http://bmjopen.bmj.com/site/about/guidelines.xhtml

Figure 2. Flow diagram of the cluster randomized controlled trial (separately) in Pakistan and Nigeria.

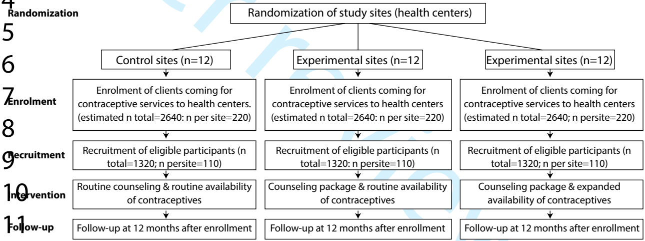

For peer review only - http://bmjopen.bmj.com/site/about/guidelines.xhtml

Figure 3. Schedule of enrolment, interventions, and assessments (SPIRIT template) for Pakistan and Nigeria (each country will use the same sampling framework)

	Enrolment	Allocation	Post-allocation			
**TIMEPOINT**	***t_{-1}*** *Initial study family planning visit*	***t_0*** *Initial study family planning visit*	***t_1*** *After initial study visit*	***t_2*** *After each follow-up visit*	***t_3*** *…*	***t_4*** *12 months*
**ENROLMENT:**						
Eligibility screen (estimate)	7920					
Informed consent	3960					
Allocation		3960	X	X	X	X
**INTERVENTIONS & CONTROL:**						
Counseling package & routine availability of contraceptives		1320	◆	—	—	◆
Counseling package & expanded availability of contraceptives		1320	◆	—	—	◆
Control: routine counseling & routine availability of contraceptives		1320	◆	—	—	◆
**ASSESSMENTS:**						
**Baseline variables:** Met need for family planning Modern contraceptive use		X				
**Primary outcomes:** Decision-making autonomy Met need for family planning			X	X	X	X
**Secondary outcomes:** Modern contraceptive use			X	X	X	X

For peer review only - http://bmjopen.bmj.com/site/about/guidelines.xhtml

SPIRIT

STANDARD PROTOCOL ITEMS: RECOMMENDATIONS FOR INTERVENTIONAL TRIALS

SPIRIT 2013 Checklist: Recommended items to address in a clinical trial protocol and related documents*

Section/item	Item No	Description	Addressed on page number
**Administrative information**			
Title	1	Descriptive title identifying the study design, population, interventions, and, if applicable, trial acronym	_1____________
Trial registration	2a	Trial identifier and registry name. If not yet registered, name of intended registry	__2___________
	2b	All items from the World Health Organization Trial Registration Data Set	__2___________
Protocol version	3	Date and version identifier	__2__________
Funding	4	Sources and types of financial, material, and other support	___15________
Roles and responsibilities	5a	Names, affiliations, and roles of protocol contributors	___1, 15______
	5b	Name and contact information for the trial sponsor	___15________
	5c	Role of study sponsor and funders, if any, in study design; collection, management, analysis, and interpretation of data; writing of the report; and the decision to submit the report for publication, including whether they will have ultimate authority over any of these activities	___15__________
	5d	Composition, roles, and responsibilities of the coordinating centre, steering committee, endpoint adjudication committee, data management team, and other individuals or groups overseeing the trial, if applicable (see Item 21a for data monitoring committee)	____15_________

For peer review only

Introduction

Background and rationale	6a	Description of research question and justification for undertaking the trial, including summary of relevant studies (published and unpublished) examining benefits and harms for each intervention	_____4______
	6b	Explanation for choice of comparators	_____7________
Objectives	7	Specific objectives or hypotheses	_____4________
Trial design	8	Description of trial design including type of trial (eg, parallel group, crossover, factorial, single group), allocation ratio, and framework (eg, superiority, equivalence, noninferiority, exploratory)	_____7________

Methods: Participants, interventions, and outcomes

Study setting	9	Description of study settings (eg, community clinic, academic hospital) and list of countries where data will be collected. Reference to where list of study sites can be obtained	________7______
Eligibility criteria	10	Inclusion and exclusion criteria for participants. If applicable, eligibility criteria for study centres and individuals who will perform the interventions (eg, surgeons, psychotherapists)	___9,11______
Interventions	11a	Interventions for each group with sufficient detail to allow replication, including how and when they will be administered	____7,8_________
	11b	Criteria for discontinuing or modifying allocated interventions for a given trial participant (eg, drug dose change in response to harms, participant request, or improving/worsening disease)	_____11________
	11c	Strategies to improve adherence to intervention protocols, and any procedures for monitoring adherence (eg, drug tablet return, laboratory tests)	_______8_______
	11d	Relevant concomitant care and interventions that are permitted or prohibited during the trial	_____8________
Outcomes	12	Primary, secondary, and other outcomes, including the specific measurement variable (eg, systolic blood pressure), analysis metric (eg, change from baseline, final value, time to event), method of aggregation (eg, median, proportion), and time point for each outcome. Explanation of the clinical relevance of chosen efficacy and harm outcomes is strongly recommended	____4__________
Participant timeline	13	Time schedule of enrolment, interventions (including any run-ins and washouts), assessments, and visits for participants. A schematic diagram is highly recommended (see Figure)	___Fig 3_____

For peer review only - http://bmjopen.bmj.com/site/about/guidelines.xhtml

Sample size	14	Estimated number of participants needed to achieve study objectives and how it was determined, including clinical and statistical assumptions supporting any sample size calculations	___Fig 3, 11___
Recruitment	15	Strategies for achieving adequate participant enrolment to reach target sample size	_____11______
**Methods: Assignment of interventions (for controlled trials)**			
Allocation:			
Sequence generation	16a	Method of generating the allocation sequence (eg, computer-generated random numbers), and list of any factors for stratification. To reduce predictability of a random sequence, details of any planned restriction (eg, blocking) should be provided in a separate document that is unavailable to those who enrol participants or assign interventions	____11_________
Allocation concealment mechanism	16b	Mechanism of implementing the allocation sequence (eg, central telephone; sequentially numbered, opaque, sealed envelopes), describing any steps to conceal the sequence until interventions are assigned	____11_________
Implementation	16c	Who will generate the allocation sequence, who will enrol participants, and who will assign participants to interventions	____12_________
Blinding (masking)	17a	Who will be blinded after assignment to interventions (eg, trial participants, care providers, outcome assessors, data analysts), and how	__11___________
	17b	If blinded, circumstances under which unblinding is permissible, and procedure for revealing a participant's allocated intervention during the trial	____11_________
**Methods: Data collection, management, and analysis**			
Data collection methods	18a	Plans for assessment and collection of outcome, baseline, and other trial data, including any related processes to promote data quality (eg, duplicate measurements, training of assessors) and a description of study instruments (eg, questionnaires, laboratory tests) along with their reliability and validity, if known. Reference to where data collection forms can be found, if not in the protocol	____12_________
	18b	Plans to promote participant retention and complete follow-up, including list of any outcome data to be collected for participants who discontinue or deviate from intervention protocols	___12__________

Data management	19	Plans for data entry, coding, security, and storage, including any related processes to promote data quality (eg, double data entry; range checks for data values). Reference to where details of data management procedures can be found, if not in the protocol	____12_________
Statistical methods	20a	Statistical methods for analysing primary and secondary outcomes. Reference to where other details of the statistical analysis plan can be found, if not in the protocol	___12__________
	20b	Methods for any additional analyses (eg, subgroup and adjusted analyses)	__12________
	20c	Definition of analysis population relating to protocol non-adherence (eg, as randomised analysis), and any statistical methods to handle missing data (eg, multiple imputation)	_12____________
**Methods: Monitoring**			
Data monitoring	21a	Composition of data monitoring committee (DMC); summary of its role and reporting structure; statement of whether it is independent from the sponsor and competing interests; and reference to where further details about its charter can be found, if not in the protocol. Alternatively, an explanation of why a DMC is not needed	_13_________
	21b	Description of any interim analyses and stopping guidelines, including who will have access to these interim results and make the final decision to terminate the trial	___12__________
Harms	22	Plans for collecting, assessing, reporting, and managing solicited and spontaneously reported adverse events and other unintended effects of trial interventions or trial conduct	___12__________
Auditing	23	Frequency and procedures for auditing trial conduct, if any, and whether the process will be independent from investigators and the sponsor	___12__________
**Ethics and dissemination**			
Research ethics approval	24	Plans for seeking research ethics committee/institutional review board (REC/IRB) approval	__13___________
Protocol amendments	25	Plans for communicating important protocol modifications (eg, changes to eligibility criteria, outcomes, analyses) to relevant parties (eg, investigators, REC/IRBs, trial participants, trial registries, journals, regulators)	___13__________

For peer review only - http://bmjopen.bmj.com/site/about/guidelines.xhtml

Consent or assent	26a	Who will obtain informed consent or assent from potential trial participants or authorised surrogates, and how (see Item 32)	___9, 13______
	26b	Additional consent provisions for collection and use of participant data and biological specimens in ancillary studies, if applicable	__14_________
Confidentiality	27	How personal information about potential and enrolled participants will be collected, shared, and maintained in order to protect confidentiality before, during, and after the trial	______13,14____
Declaration of interests	28	Financial and other competing interests for principal investigators for the overall trial and each study site	_____15__________
Access to data	29	Statement of who will have access to the final trial dataset, and disclosure of contractual agreements that limit such access for investigators	______13_________
Ancillary and post-trial care	30	Provisions, if any, for ancillary and post-trial care, and for compensation to those who suffer harm from trial participation	_____13__________
Dissemination policy	31a	Plans for investigators and sponsor to communicate trial results to participants, healthcare professionals, the public, and other relevant groups (eg, via publication, reporting in results databases, or other data sharing arrangements), including any publication restrictions	___14___________
	31b	Authorship eligibility guidelines and any intended use of professional writers	_____14__________
	31c	Plans, if any, for granting public access to the full protocol, participant-level dataset, and statistical code	___14___________
**Appendices**			
Informed consent materials	32	Model consent form and other related documentation given to participants and authorised surrogates	__Submitted__
Biological specimens	33	Plans for collection, laboratory evaluation, and storage of biological specimens for genetic or molecular analysis in the current trial and for future use in ancillary studies, if applicable	__NA________

*It is strongly recommended that this checklist be read in conjunction with the SPIRIT 2013 Explanation & Elaboration for important clarification on the items. Amendments to the protocol should be tracked and dated. The SPIRIT checklist is copyrighted by the SPIRIT Group under the Creative Commons "Attribution-NonCommercial-NoDerivs 3.0 Unported" license.

For peer review only - http://bmjopen.bmj.com/site/about/guidelines.xhtml
